# Characterization of Peptaibols Produced by a Marine Strain of the Fungus *Trichoderma endophyticum* via Mass Spectrometry, Genome Mining and Phylogeny-Based Prediction

**DOI:** 10.3390/metabo13020221

**Published:** 2023-02-03

**Authors:** Gleucinei S. Castro, Thiago F. Sousa, Gilvan F. da Silva, Rita C. N. Pedroso, Kelly S. Menezes, Marcos A. Soares, Gustavo M. Dias, Aline O. Santos, Michel E. B. Yamagishi, Jéssica V. Faria, Ana H. Januário, Hector H. F. Koolen

**Affiliations:** 1 Metabolomics and Mass Spectrometry Research Group, Amazonas State University (UEA), Manaus 690065-130, Brazil; 2 Graduate Program in Biotechnology, Federal University of Amazonas (UFAM), Manaus 69080-900, Brazil; 3 Embrapa Amazônia Ocidental, Manaus 69010-970, Brazil; 4 Núcleo de Pesquisa em Ciências Exatas e Tecnológicas, Universidade de Franca, Franca 14404-600, Brazil; 5 Instituto de Biociências, Universidade Federal de Mato Grosso (UFMT), Cuiabá 78060-900, Brazil; 6 Centro de Ciências Naturais e Humanas, Universidade Federal do ABC (UFABC), São Bernardo do Campo 09606-070, Brazil; 7 Embrapa Digital, Campinas 13083-970, Brazil; 8 Centro de Estudos Superiores de Tefé, Universidade do Estado do Amazonas (UEA), Tefé 69470-000, Brazil

**Keywords:** de novo sequencing, marine fungus, molecular networking, nonribosomal peptides, synteny analysis

## Abstract

Trichoderma is recognized as a prolific producer of nonribosomal peptides (NRPs) known as peptaibols, which have remarkable biological properties, such as antimicrobial and anticancer activities, as well as the ability to promote systemic resistance in plants against pathogens. In this study, the sequencing of 11-, 14- and 15-res peptaibols produced by a marine strain of *Trichoderma* isolated from the ascidian *Botrylloides giganteus* was performed via liquid chromatography coupled to high-resolution tandem mass spectrometry (LC-MS/MS). Identification, based on multilocus phylogeny, revealed that our isolate belongs to the species *T. endophyticum*, which has never been reported in marine environments. Through genome sequencing and genome mining, 53 biosynthetic gene clusters (BGCs) were identified as being related to bioactive natural products, including two NRP-synthetases: one responsible for the biosynthesis of 11- and 14-res peptaibols, and another for the biosynthesis of 15-res. Substrate prediction, based on phylogeny of the adenylation domains in combination with molecular networking, permitted extensive annotation of the mass spectra related to two new series of 15-res peptaibols, which are referred to herein as “endophytins”. The analyses of synteny revealed that the origin of the 15-module peptaibol synthetase is related to 18, 19 and 20-module peptaibol synthetases, and suggests that the loss of modules may be a mechanism used by *Trichoderma* species for peptaibol diversification. This study demonstrates the importance of combining genome mining techniques, mass spectrometry analysis and molecular networks for the discovery of new natural products.

## 1. Introduction

*Trichoderma* (*Hypocreaceae*) was described by Christian Hendrik Persoon in 1794 [1], and currently represents one of the genera with the largest number of described species in the Fungi kingdom [2], with more than 500 valid species, according to the Index Fungorum (http://www.indexfungorum.org (accessed on 30 December 2022)). Species of the genus *Trichoderma* can inhabit innumerable ecosystems, both terrestrial and aquatic, and can be isolated from the interior of other organisms, such as plants (endophytes), insects, marine organisms and even humans (facultative pathogens), which reveals its high adaptability to different conditions [3,4,5,6,7,8].

The species of the genus *Trichoderma* are commonly used in agriculture to promote plant growth, phosphate solubilization, nitrogen fixation, and the induction of resistance in plants, and as a biological control agent; numerous patents have been filed in this regard. Agricultural products obtained from *Trichoderma asperellum,* such as ECO-T, are marketed worldwide [9,10,11,12,13,14,15,16].

In addition to agricultural applications, *Trichoderma* is also a valuable source of secondary metabolites, and contributes to several carbon skeletons that have the most diverse biological activities [7,17]. Among the classes of metabolites that are produced by species of *Trichoderma*, alkaloids [18], terpenes [19], and peptides [20,21] stand out.

Among the peptides produced by *Trichoderma,* the class of nonribosomal peptides (NRPs), known as peptaibols, can be highlighted for its wide range of biotechnological applications. These peptides consist of chains of 5 to 20 amino acid residues that have high levels of lipophilicity, which are especially due to the presence of noncanonical amino acids, such as *α*-aminoisobutyrate (Aib) and isovaline (Iva), along with alanine (Ala), valine (Val), leucine (Leu) and isoleucine (Ile). As well as these, peptaibols also possess the N-terminal portion acetylated and the C-terminal in the form of an amino alcohol [21,22,23].

Peptaibols have great potential for generating new antimicrobial drugs, since they act by destabilizing the lipid bilayer instead of a specific target, which reduces the possibility of resistance mechanisms [24]. In addition to their antimicrobial potential, there are studies that indicate that peptaibols are promising molecules in several areas of medicine and may lead to the development of drugs against malaria [25] and cancer [26].

Peptaibols are synthesized by mega synthetases called NRPSs (nonribosomal peptide synthetases) of the multimodular type, which are made up of a series of domains. A typical module consists of the presence of condensation, adenylation and thiolation domains. Adenylation domains (A) select and activate aminoacyl substrates, which are then positioned in the phosphopantetheine prosthetic group of a thiolation domain (T). The thiolation domain can then deliver the aminoacyl substrate to the condensation domain (C), where it is coupled with the upstream nascent peptide. In addition to these minimal domains (A-T-C), modified domains within a module may include epimerization (E), formylation, cyclization, oxidation and reduction domains. Another domain typically found in the final module is the thioesterase (TE) domain, which releases the complete peptide [27,28,29].

Recently, several tools have permitted the genome mining and identification of biosynthetic gene clusters (BGCs) related to NRPSs [30,31]. However, although it is possible to make a prediction of the possible nonribosomal peptides (NRPs) based on the adenylation domains, it is still difficult to accurately predict which of these putative NRPs are correct, mainly due to the post-assembly modifications of amino acids carried out by decoration genes present in the BGC [32]. Nevertheless, new tools that incorporate genome and spectral data analysis have enabled the discovery of new bioactive NRPs [33].

Despite being a previously described fungal species, information about the biotechnological potentials and chemical profile of *Trichoderma endophyticum* is scarce in the literature. So far, only one endophytic specimen has been assayed for plant growth promotion, in which it was found that, in four edaphoclimatic locations, *T. endophyticum* displayed a positive impact on promoting the growth of soybean plants without causing any symptoms of phytotoxicity when applied via the seeds [34]. During our research on the metabolite profiles of Brazilian *Trichoderma* strains, we found that a marine isolate was capable of producing compounds with high *m*/*z* and fragmentation patterns related to peptaibols. Therefore, the present study reports the species level identification of this strain as *T. endophyticum*, as well as a series of peptaibols named here as “endophytins” via a combination of genomic analyses and manual annotation of product ion mass spectra with the aid of molecular networking.

## 2. Materials and Methods

### 2.1. Collection and Fungal Acquisition

A sample of the ascidian *Botrylloides giganteus* (50 g) was collected in October 2015 in the municipality of Ilha Bela on the coast of the state of São Paulo, Brazil (23°46′26.95″ S, 45°21′21.26″ W). Initially, the isolation procedure comprised the superficial disinfection of the ascidian with ethanol 70% for 45 s, followed by washing with sterile sea water (3x). Then, 1 cm^2^ square pieces of the ascidian were inoculated in Petri dishes containing a culture medium consisting of malt (30 g/L) and agar (15 g/L) in sterile sea water supplemented with tetracycline (50 mg/L) and chloramphenicol (50 mg/L). After four days, the fungal strain emerged, which was then transferred to other Petri dishes containing the same culture medium and further purified by monosporic cultures. Strain 5A7 initially isolated by A.H.J. was donated to the research group led by H.H.F.K. and later deposited under the code MMSRG85 (SISGEN Register A6BA963).

### 2.2. Fungal Identification

DNA extraction was performed using the CTAB method [35] using mycelium grown in potato dextrose broth for 3 days at 28 °C at 125 rpm. Polymerase chain reactions (PCR) were used for amplification of the internal transcribed spacer (ITS) region, and partial sequences of the elongation factor 1-α (*tef-1α*) and RNA polymerase II (*rpb2*) genes were prepared using the Easytaq kit (Synapse Biotechnology) with three pairs of primers: ITS1 (TCCGTAGGTGAACCTGCGG) and ITS4 (TCCTCCGCTTATTGATATG) [36], fRPB2-5f (GAYGAYMGWGATCAYTTYGG) and fRPB2-7cr (CCCATRGCTbTGTYYY) [37] and EF-1αF (ATGGGTAAGGARGACAAGAC) and EF-1αR (GGARGTACCAGTSATCATGTT) [38].

The PCR condition for amplification of all loci was as follows: initial denaturation at 95 °C for 3 min, 35 denaturation cycles at 95 °C for 45 s, hybridization temperature at 55 °C for 45 s, extension at 72 °C for 1 min, and final extension at 72 °C for 5 min. PCR products were subjected to agarose gel electrophoresis (1.5%) for confirmation of amplicon length using the 1 kb marker (Invitrogen, ThermoFisher, Waltham, MA, USA). The purification of amplicons for sequencing was performed using exoSAP-IT (ThermoFisher; catalog code: 78200.200.UL, Waltham, MA, USA) in accordance with the manufacturer’s recommendations. The sequencing reactions were performed in a volume of 10 µL, containing 2 µL of ultrapure water, 1.5 µL of Big Dye buffer, 0.5 µL of Big Dye Terminator v 3.1 (ThermoFisher), 1 µL of primer and 5 µL of purified PCR product. The cycling conditions were 96 °C for 1 min, followed by 35 cycles at 96 °C for 15 s, 50 °C for 15 s and 60 °C for 4 min. Sequencing was performed using a 3500 Genetic Analyzer sequencer (Thermo Fisher).

### 2.3. Phylogenetic Analyses

The sequences of *tef1-α* and *rpb2* were individually aligned with the MAFFT tool in UGENE software [39]. Alignments were plotted on the IQ-TREE platform [40] and a phylogenetic analysis using maximum likelihood (ML) was performed with the concatenated sequences of *tef1-α* and *rpb2*. Bayesian inference (BI) was performed using CIPRES (www.phylo.org (accessed on 30 December 2022)).

The ML analysis included 1000 replicates (bootstrap) using all sites with the best template selected by IQ-TREE. The BI was based on the model adopted in PAUP*4 and Mrmodeltest2 (v.2) [41]. All the sites were considered, and analysis was carried out for ten million generations, with the first 25% of trees discarded and burned using the MrBayes tool (v. 3.7) [42]. The posterior probability (PP) and the topology of the tree were visualized with the iTOL platform (itol.embl.de). The consensus tree of ML and BI analyses was manually generated from the topology obtained via the iTol platform in the ML analysis with bootstrap values plus PP values generated via Bayesian inference, using the software CorelDraw, 2020 edition [43].

### 2.4. Fungal Fermentation and Extraction

To explore the ability of *T. endophyticum* MMSRG85 to biosynthesize peptaibols, three square plugs of agar (3 cm^2^) containing mycelium and spores of the strain were inoculated into three Erlenmeyer flasks (three plugs per flask) containing 20 g of parboiled rice and 70 mL of a solution containing sodium nitrate (NaNO_3_, 0.3 g/L), monopotassium phosphate (KH_2_PO_4_, 0.1 g/L), magnesium sulfate (MgSO_4_.7H_2_O, 0.05 g/L) and potassium chloride (KCl, 0.05 g/L). Then, the flasks were incubated for a period of 14 days at room temperature (ca. 25 °C). After this period, the fungal material was extracted once by cold maceration with 100 mL of ethyl acetate overnight, and the organic phases of the three flasks were combined, and evaporated under reduced pressure to give the crude extract.

### 2.5. Genome Mining and Prediction of the Peptaibol Modular Assembly

The complete genome was sequenced using the 150 bp paired-end Illumina platform. The “de novo” assembly was performed using the SPAdes [44]. The genome assembly resulted in 40,136,932 bp with 294X coverage and 175 scaffolds with an N50 of 3103595. The longest scaffold presented a size of 7.46 Mpb.

The genome of *T. endophyticum* MMSRG85 was submitted to the FungiSMASH platform (https://fungismash.secondarymetabolites.org/), accessed on 16 December 2022, for the prediction of biosynthetic gene clusters (BGCs). The amino acid sequence of the *Trichoderma virens* TEX1 protein (XP_013953110.1) was used to locate the scaffolds containing the BGCs related to the biosynthesis of peptaibols using the BLASTp tool in Web-Server 2.0.

The BGCs related to the production of peptaibols were re-annotated using the MAKER software available on the Galaxy Australia platform (usegalaxy.org.au) using *Fusarium graminearum* as a reference. The adenylation domains of NRPSs already characterized in the literature were used to generate a dataset (Table S1). For this, the prediction of binding sites of adenylation domains was performed via the PKS/NRPS analysis website (http://nrps.igs.umaryland.edu/ (accessed on 30 December 2022)). The constructed dataset was used for the prediction of substrate affinity through phylogenetic inference using the ML method with 1000 replicates on the IQ-TREE platform. The phylogram was visualized using iTOL and edited with the help of CorelDraw software, version 2020.

The clinker & clustermap pipeline.js was used for synteny analysis. The comparison in the BGCs and similarity searches were based on the tblastx algorithm in clinker and the visualization of the synteny was performed via clustermap.js [45].

### 2.6. LC-MS/MS Analysis

The AcOEt extract of the MMSRG85 strain was analyzed using high-performance liquid chromatography coupled to high resolution tandem mass spectrometry (LC-MS/MS) using a chromatograph (Nexera X2, Shimadzu, Kyoto, Japan) coupled to a spectrometer (microtof II, Bruker Daltonics, Billerica, MA, USA). The data were obtained using an electrospray ionization source operating in positive mode with the following parameters: capillary voltage, 3.5 kV; drying gas temperature, 220 °C; flow, 10 L/min; nebulizer gas at a pressure of 5 bar (N_2_). Separations were performed on a Luna C18 column (Phenomenex, 250 × 4.6 mm, 5 µm) with a final injection volume of 10 µL. The mobile phase consisted of a 0.1% solution of acetic acid (solvent A) methanol (solvent B), in which a gradient ranging from 5% to 100% of B during 65 min, followed by 10 min of 100% B with a flow rate of 1 mL/min, was applied. Calibration of the *m*/*z* measurement was performed using a sodium formate solution (1 mg/mL). An untargeted method with “scan Auto MS/MS” (data dependent analysis) mode was applied for fragmentation, selecting the five most intense precursor ions from each MS scan, with a collision energy gradient from 20 to 65 eV. The results were processed using Software Data Analysis v. 4.2 [46] (Bruker Daltonics).

### 2.7. Molecular Networking

The product ion spectra (MS/MS) of the LC-MS/MS analysis of the extract of the MMSRG85 strain were analyzed and organized in molecular networks using the GNPS platform (http://gnps.ucsd.edu (accessed on 30 December 2022)) [47]. The MS/MS spectra were converted to the mzXML format using the MS-Convert software (v. 3.0.21132) [48] and then uploaded to the GNPS platform. The parameters for generating molecular networks were as follows: precursor ion mass tolerance of 0.05 Da, product ion tolerance of 0.5 Da and fragment ion cutoff line above 10 counts. Molecular networks were generated using at least four combined peaks and with a cosine score of 0.65. Data was visualized using Cytoscape software (v. 3.7.0) [49]. The characterization of the substances was carried out in comparison with the data of the libraries deposited in the GNPS, as well as by manual interpretation of the MS/MS spectra using the DataAnalysis software (v. 4.2) in comparison with the database of the literature. The molecular network used can be accessed on the GNPS website via the following link: https://gnps.ucsd.edu/ProteoSAFe/result.jsp?task=e55a2b672d394d4d829f27a1a8d338d9&view=network_components (accessed on 19 July 2020).

## 3. Results

### 3.1. Fungal Phylogenetic Identificatio

**Figure 1:**
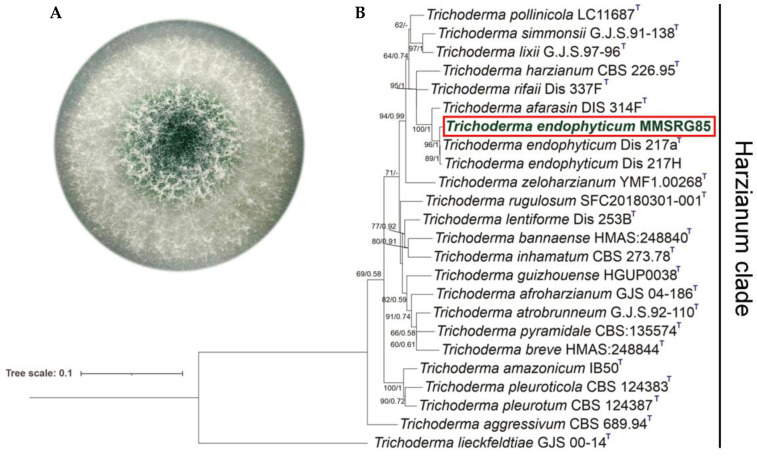
(**A**) Morphology in PDA medium of *T. endophyticum* MMSRG85 strain isolated from ascidian *Botrylloides giganteus*. (**B**) Phylogram generated by the concatenation of the sequences tef1-α and rpb2 of *Trichoderma* species of the clade *Harzianum*. The numbers indicate the support of the branches (ML/PP). The strain analyzed in this study is marked in green bold text and with a red box.

Based on the concatenated alignment of the sequences *tef1-α* (OQ291284) and *rpb2* (OQ291285) of the closest species of the clade *Harzianum*, we found 1583 characters, including the gaps (*tef1-α*: 556 and *rpb2*: 1027). The best-fit evolution model for BI analysis adopted by PAUP*4 was HKY + I+G for *tef1-α* and SYM + G for *rpb2*. For the ML analysis, the best-fitting evolution model was HKY + F + I + G4 for *tef1-α* and TNe + G4 for *rpb2*. The MMSRG85 isolate was grouped with the *Trichoderma endophyticum* Dis217a strain with high PP and bootstrap support (Figure 1). The identity analysis of the sequences *tef1-α* and *rpb2* revealed that the *T. endophyticum* MMSRG85 strain has identity values > 99% for both barcodes.

### 3.2. Genome Mining

**Figure 2:**
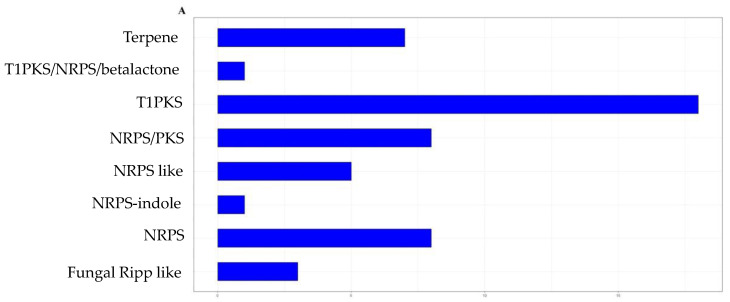

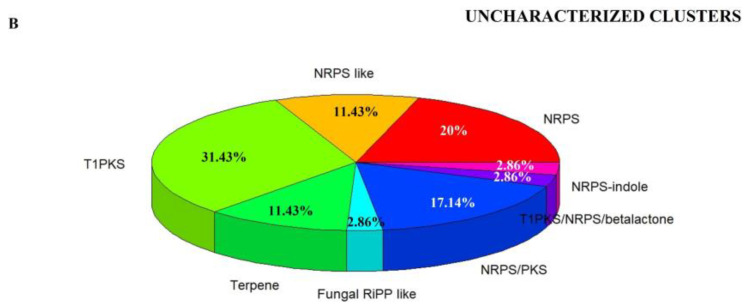
Biosynthetic gene clusters of *Trichoderma endophyticum* MMSR85. (**A**) Identification and number of biosynthetic gene clusters in *Trichoderma endophyticum* MMSRG85 according to their secondary metabolite class: NRPSs (nonribosomal peptide synthetases), NRPS-like (NRPS-like fragment), T1PKS (type I polyketide synthetase), terpene, fungal RiPP-like (fungal, unspecified, ribosomally synthesized and post-translationally modified peptide product (RiPP) cluster), hybrids (refers to BGCs that contain genes that code for more than one type of enzyme-synthesizing molecule of different classes). (**B**) Distribution of BGCs based on similarity with BGCs deposited in the MIBiG (Minimum Information about a Biosynthetic Gene Cluster) database, BGCs with zero similarity indicate that these may be related to biosynthesis of new natural products or are clusters for known molecules whose biosynthesis pathway has not yet been characterized.

Genome mining resulted in the identification of 53 BGCs, with eight related to NRPS biosynthesis, five NRPS-like, 18 for T1PKS, seven for terpenes, three fungal RiPP-like clusters and 12 hybrid BGCs, of which were eight NRPSs/T1PKSs, one T1PKS/NRPS/betalactone, one cluster for NRPS-indole, and two fungal RiPP-like/NRPSs (Figure 2, Table S2). Of these BGCs, seven presented 100% similarity with the clusters deposited in the MIBIG repository, and are related to the biosynthesis of the compounds alternariol, clavaric acid, koraiol, dimethylcoprogen, aculeacin A, naphthopyrone, choline and 1,3,6,8-tetrahydroxynaphthalene. The search for peptaibol synthetases in the genome of *T. endophyticum* strain MMSRG85 resulted in two stronger hits, one located in scaffold 8 and the other in scaffold 19.

### 3.3. Analysis of 14-Module NRPS and Prediction of Peptaibol Assembly

The BGC located in scaffold 8 (BGC 8.3), whose core gene presents 50.2 Kb, encodes an NRPS with a starter presenting the domains of PKS (KS-AT-ACP), indicating that this is a hybrid of PKS-NRPS, and presents 14 modules and contains the minimal domains (A-T-C). Phylogenetic analysis of all adenylation domains present in the PKSs-NRPSs of 14 modules of *T. endophyticum* MMSRG85 were grouped with the peptaibol synthetase NRPS2 domains of *T. virens* Tv29-8 (Figure S1), which produces 14-res (harzianin HC-derivatives) and 11-res (harzianin HB-derivatives) peptaibols. The synteny analysis reveals a high level of identity between the core gene and other BGC 8.3 genes with *T. virens* Tv29-8 [10] (Figure S1).

The phylogram obtained for the 14-module hybrid PKS-NRPS showed that the adenylation domains that accept the proline (Pro) substrate (13, 9 and 5) were grouped into a single monophyletic clade, thus indicating the high specificity of these domains (Figure S2). The assembly position of α-aminoisobutyric acid (Aib) can be inferred in the adenylation domains corresponding to modules 1, 4, 8 and 12, while 3, 7, 10 and 11 show promiscuity for leucine/isoleucine or valine/isovaline due to the similarity of the substrates. The amino acids glycine, glutamine and glutamic acid are predicted for position 2, and are only grouped with the adenylation domain of module 2 of NRPS2 of *T. virens*, which occupies a distinct phylogenetic position in relation to the other adenylation domains that have affinity for these substrates. The predicted amino acid for module 6 is alanine (Ala). The adenylation domain of module 14 was grouped with adenylation domains of the C-terminal portion of the NRPSs (Figure S2).

### 3.4. Analysis of 15-Module NRPS and Prediction of Peptaibol Assembly

**Figure 3:**
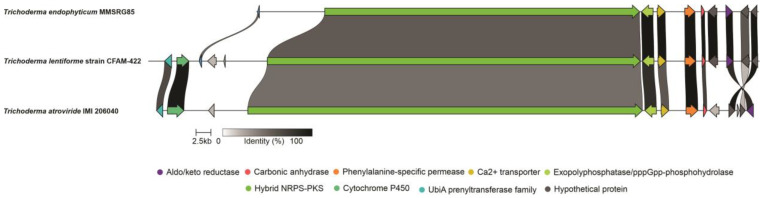
Synteny analysis between BGC 19.1 encoding a PKS-NRPS of 15 modules of *Trichoderma endophyticum* strain MMSRG85 and the BGC containing the TEX1 gene related to the biosynthesis of 18-res peptaibols in *Trichoderma atroviride* IMI 206040 and a putative peptaibol synthetase of *Trichoderma lentiforme*. The synteny analysis shows conserved genes among the BGCs related to the production of peptaibols from *T. endophyticum* strain MMSRG85 (15 modules), *Trichoderma lentiforme* (18 modules) and *Trichoderma atroviride* (19 modules), revealing that the loss or gain of modules in these NRPSs may favor the diversity of the peptaibols produced (Figure S2).

The NRPS related to 15-res peptaibol synthesis is a 52.9 kb PKS-NRPS hybrid located in scaffold 19 (BGC 19.1). The analysis of NRPS domains reveals that there was a loss of the KS domain in the start region of the protein and that it has 15 modules. The synteny with *Trichoderma lentiforme* strain CFAM-422 and *Trichoderma atroviride* IMI 206040 confirms the presence of genes related to the biosynthesis of peptaibols. In addition to the deletion of the KS domain, a different number of modules were observed in *T. lentiforme* and *T. atroviride*, which have 18 and 19 modules, respectively. However, upstream genes remain conserved with high identity (Figure 3).

**Figure 4:**
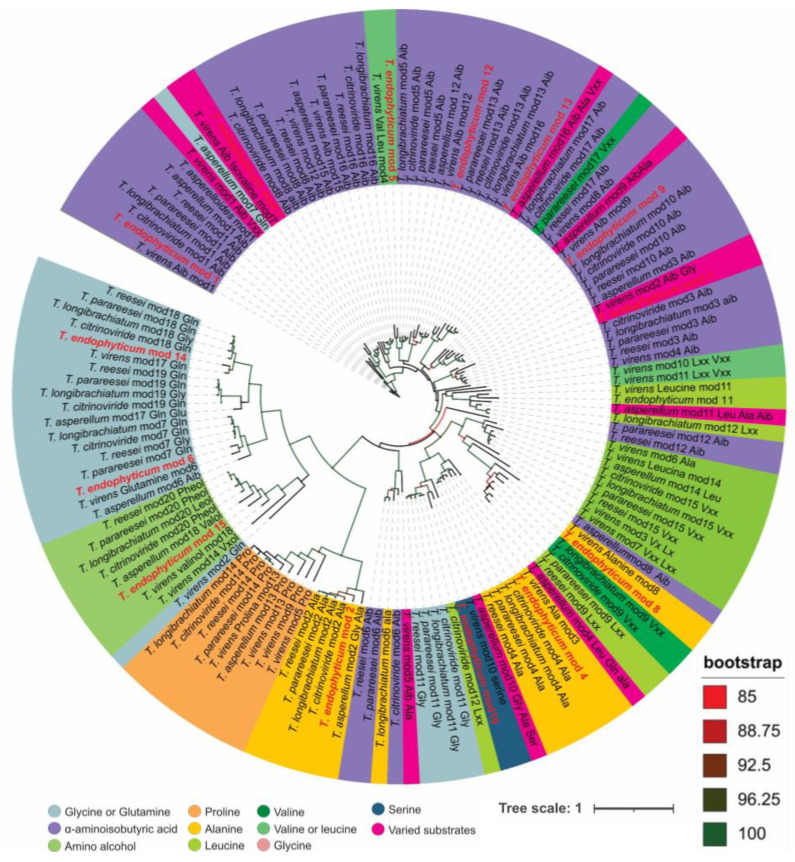
Phylogeny of 15-res NRPS adenylation modules.

The in silico prediction of the amino acid sequence synthesized by the PKSs-NRPSs of 15 modules of *T. endophyticum* strain MMSRG85, which was performed based on the phylogenetic analysis of the adenylation domains, revealed that module 2 of *T. endophyticum* is phylogenetically more related to module 2 of the peptaibol synthetases of 20 modules present in the clade *Longibrachiatum*, which have specificity for the amino acid alanine and which was the only adenylation domain that did not group with sequences of NRPS TEX1 from *T. virens* (Figure 4).

### 3.5. Annotation of the Peptaibols

The adenylation domain of module 8 is most closely related to a domain that has affinity for alanine in *T. virens*, but forms a clade with domains that have affinity for varied substrates such as Aib, leucine/isoleucine, valine/isovaline, and alanine. In silico analysis of the amino acid sequence synthesized by the adenylation domain of module 4 revealed the affinity of the domain for alanine. The domains corresponding to modules 1, 5, 9, 12 and 13 are similar to domains that have affinity for Aib. The adenylation domain of module 10 is grouped with a domain that has affinity for serine, while the domains corresponding to modules 3 and 7 are predicted for varied amino acids, and domains 6 and 14 correspond to glycine, glutamine or glutamic acid. Domain 15 corresponds to the modules of the C-terminal portion of peptaibol synthetases (Figure 4).

**Figure 5:**
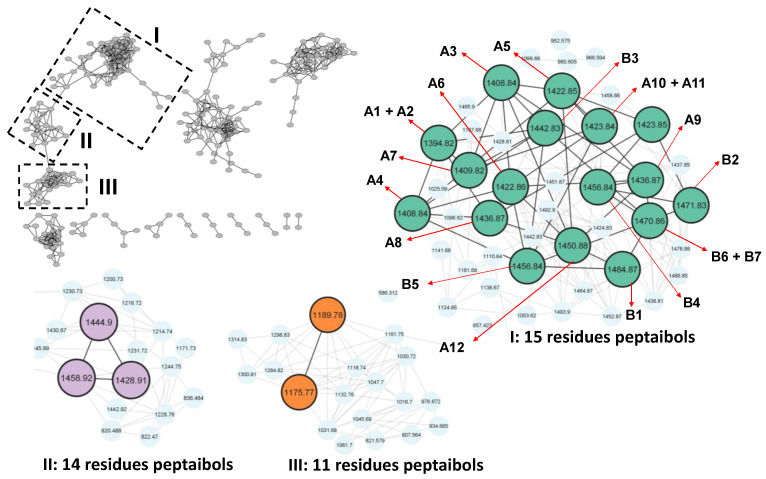
Annotation of the molecular network of peptaibols derived from the ethyl acetate extract. Green nodes: putative sequences of 15-res. Lilac nodes: peptaibols of 14-res. Orange nodes: peptaibols of 11-res.

The high *m*/*z* observed in several peaks of the total ion chromatogram of *T. endophyticum* strain MMSRG85 indicated that, under the evaluated conditions, this species could produce peptides (Figure S3, Figure 5). The biosynthesis of a complex mixture of NRPs of the peptaibol class with 11, 14 and 15-res was confirmed after manual analysis of the MS/MS spectra and annotation using molecular networks.

In the molecular network generated, several connected nodes were observed, of which the largest network (85 nodes) was of interest for the identification of peptaibols, as it presented nodes referring to monocharged ions with *m*/*z* between 1095 and 1493, which is indicative of natural products of high molecular weight. Regarding the comparison with the libraries present on the GNPS platform, the node with *m*/*z* 1444.9 presented a significant hit, which, through spectral comparison, indicated the possible presence of a molecule related to harzianin HC XIII (Figures S4 and S5). However, the manual analysis (Figure S5) of these compounds precluded their identifications, since several peptaibol nomenclatures possess similar sequences to harzianins. Even though this compound could not be fully identified, it served as a seed to investigate the remaining nodes of group II (Figure 5).

The analysis of group I indicated several nodes close to the seeds, but which were not directly marked in any GNPS library or other available tools. Thus, the annotation of the sequences was only possible through the manual interpretation of the MS/MS spectra of the different chromatographic peaks of the LC-MS/MS analysis, which allowed the characterization of 21 peptaibols (compounds 1, 2, 5–8, 10–12, 14–15, 17–20, 22–27) containing 15 amino acid residues, and which are structurally similar to each other.

**Figure 6:**
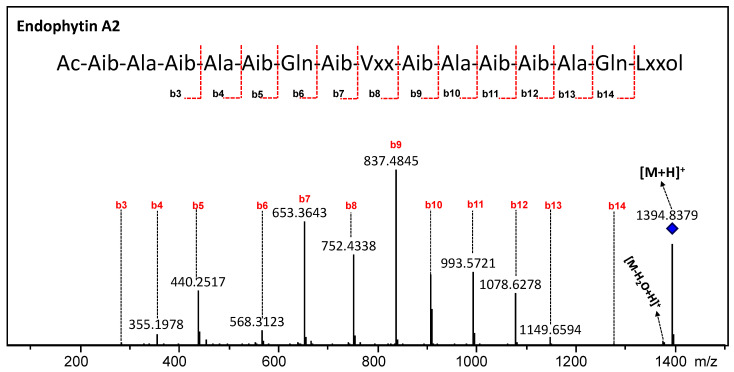
MS/MS sequencing of peptaibol endophytin A2 (diamond). The sequences are given in standard spelling code (Vxx, Val/Iva, Lxx, Leu/Ile and Lxxol represent the C-terminal amino alcohol).

In order to exemplify the identification logic for the amino acid sequences of the unknown peptaibols, the fragmentation of the compound with *m*/*z* 1394.8370 [M+H]^+^, C_63_H_115_N_17_O_18_, −0.07 ppm) was analyzed as a model for the other peptaibols present (Figure 6). In the scanning spectrum of product ions, mostly b-series ions are observed, which comprise ions from b14 to b3, moving in the direction of the C-terminal portion to the N-terminal portion. In the C-terminal portion, the fragments *m*/*z* 1394 → *m*/*z* 1376 (-H_2_O) and *m*/*z* 1394 → *m*/*z* 1277 (b14, −117 u) confirmed that this portion is the amino alcohol leucinol/isoleucinol (Lxxol). Subsequently, characteristic losses of amino acid residues such as glutamine (Gln, −128 u), alanine (Ala, −71 u), Aib (−85 u), valine/isovaline (Vxx, −99 u) and leucine/isoleucine (Lxx, −113 u) were observed (Figure 6). Although a good coverage of fragments was observed in terms of amino acid sequence, fragments b1 and b2 could not be seen in the MS/MS experiments. As a result, we had to proceed with a differentiated interpretation for the N-terminal portion, in which we performed the characterization of the N-terminal moiety through analysis of the b3 fragment (*m*/*z* 284.1553). Considering that the recorded *m*/*z* of product ion can only display the observed *m*/*z* via the combination of two Aib residues, one Ala residue and an acetyl group (42 + 85 + 71 + 85), we still had to establish the order of the amino acids, which was not possible via the product ion spectrum. To solve this question, we combined the information of genome mining with a phylogeny analysis of the adenylation modules of the peptaibol synthetase to ensure the sequence of this peptide portion. When carrying out the analysis of the phylogeny data, it was observed that module 2 plays a central role in the attribution of the sequence of the three amino acid residues of the N-terminal portion. This was possible through the phylogeny because it showed that this is not a promiscuous module, based on the high bootstrap support (>92%), which indicates the incorporation of Ala at amino acid position 2. In this way, positions 1 and 3 can only be filled by Aib according to the recorded *m*/*z* b3 ion. Moreover, this is reinforced by the phylogeny of module 1, which displayed a 100% bootstrap for the incorporation of Aib. Thus, the sequence of compound 2 was determined as Ac-Aib-Ala-Aib-Ala-Aib-Gln-Aib-Vxx-Aib-Ala-Aib-Aib-Ala-Gln-Lxxol, thus named as endophytin A2.

The 21 new sequences of peptaibols identified in this work (Table 1 and Table S3) preserve the motifs Aib-Ala-Aib-Ala (residues 1 to 4) and Aib-Ala-Aib-Aib (residues 9 to 12). These data were confirmed based on the in silico prediction of the adenylation domains of NRPS located in cluster 19.1 of *T. endophyticum* strain MMSRG85 (Figure 4). The peptaibols characterized here are divided into two groups: endophytins A1–A13 that present leucinol/isoleucinol as a C-terminal amino alcohol and endophytins B1–B8, which have the amino alcohol phenylalanilol in the same position. Regarding the differences in amino acid sequences, the A and B series analogs show variations only in residues at positions 5 (Aib, Ala or Vxx), 7 (Aib or Vxx), 8 (Lxx or Vxx), 13 (Aib or Ala) and 14 (Gln or Glu) (Table 1).

## 4. Discussion

After the taxonomic description of *Trichoderma endophyticum* isolated from Neotropical plants, few other studies have reported this species in other environments [50,51,52]. To date, only one study on the application of *T. endophyticum* is reported in the literature and confirms the status of this species as a growth promoter in *Glycine max* [34]. In the present study, *Trichoderma endophyticum* was identified via multilocus phylogenetic analysis using sequence data from the barcodes *tef1-α* and *rpb2* and, to our knowledge, this is the first report of this species in Brazil, as well as the first time it has been isolated from marine environments. In addition, the complete genome of a representative of the species was obtained for the first time, the genome mining of which made it possible to identify the biosynthetic potential of the strain MMSRG85.

More than 50 BGCs were identified in the genome of *T. endophyticum*, and 66% showed no similarity with the BGCs deposited in the MIBiG (Minimum Information about a Biosynthetic Gene Cluster) database, which is a repository for biosynthetic gene clusters of known function [53]. These uncharacterized BGCs may be related to the biosynthesis of new molecules or already known molecules whose biosynthetic pathways have not yet been defined or deposited in the repository (Table S1). 

Among the BGCs with a similarity of 88 to 100% with the BGCs deposited in the MIBIG database, pathways for tricholignan production were identified (BGC 11.2). This molecule has been linked to the ability of iron uptake by plants, and helps in promoting plant growth, suggesting a possible use in agricultural applications [54]. Another BGC was related to clavaric acid (BGC 2.2), which is a triterpene capable of inhibiting the protein farnesyl transferase, and for this reason is a promising anticancer substance that targets the oncogene Ras (a mutated gene in several types of cancer) [55].

In the genome of the isolate MMSRG85, only one of the nine BGCs related to the synthesis of nonribosomal peptides, including NRPS and PKS-NRPS hybrids, showed similarity with the BGCs characterized in the MIBIG repository, which suggests that about 90% of these clusters may be associated with the production of new NRPSs (Table S1). Among the BGCs related to PKS-NRPS hybrids, two were considered to have potential for peptaibol production; cluster 8.3 showed similarity with the BGCs related to the production of harzianins HC, while cluster 19.1 was related to the cluster responsible for producing peptaibols of modules 18, 19 and 20 [56,57]. Interestingly, the peptaibol synthetase of cluster 19.1 has only 15 modules. The synteny analysis using the TEX1 gene of different *Trichoderma* species (Figure 4A) suggests the loss of *T. endophyticum* modules is a probable mechanism of diversification of the core gene for the production of peptaibols of varying sizes.

*T. endophyticum* is a producer of peptaibols of 11-, 14- and 15-residues, of which, peptaibols of 11- and 14-res have been identified as molecules with sequences related to the hypomuricin and harzianin HC series, respectively. These peptaibols are related to the PKS-NRPS hybrid present in cluster 8.3. Some peptaibols of the harzianin HC series, such as harzianin HC I, harzianin HC III, harzianin HC XI, harzianin HC XII, harzianin HC XIV, harzianin HC X and harzianin HC XV, have known antiplasmodium activity [53].

The 15-res peptaibols are produced by the PKSs-NRPSs of cluster 19.1 and represent new peptaibols referred to herein as endophytins. These peptaibols present similarities with pentadecaibins II, III and IV from 15-res biosynthesized using marine *Trichoderma* sp. MMS1255 [58]. However, these peptaibols exhibit sequence patterns that remain different from endophytins, since they have an Aib-Ala-Aib-Ala sequence in the N-terminal portion, while pentadecaibins have four Aib-Gly-Ala-Leu amino acid residues in the N-terminal portion.

The results of this study identified two mechanisms of peptaibol diversification in *T. endophyticum*. The peptaibol synthetase present in cluster 8.3 produced peptaibols of different sizes (11- and 14-res) through the “module skipping” mechanism [10]. On the other hand, the peptaibol synthetase present in cluster 19.1 suffered a loss of modules, but maintained its up- and downstream genes conserved with the clusters of peptaibol synthetases of modules 18, 19 and 20 of *T. lentiforme, T. atroviride* and *T. longibrachiatum*, respectively. The mechanism of “module skipping” has already been reported for the peptaibol synthetases of *Trichoderma virens* and *Trichoderma* sp. RK10-F026 [10,59,60]; while, to our knowledge, the loss of modules revealed by the analysis of synteny has never been reported for peptaibol synthetases.

The in silico prediction of 14- and 15-res peptaibols was performed using a phylogenetic approach combined with manual interpretation of the spectra and molecular networking. This approach permitted the resolution of the N-terminal portion of the endophytins and provided support for the alanine deposition in the *m*/*z* 284.1553 fragment (Figures S1, S2 and S11–S30). Amino acid prediction via phylogenetic analyses using the adenylation domains of peptaibol synthetases has already been used in other works [60,61,62]. The main use of this tool is in the identification of the amino acid position based on the collinearity of the peptide and the NRPS modules. Combined with mass spectrometry (HPLC/MSMS), the deduction helps in identifying masses in which fragmentation of all amino acids does not occur, thus increasing the number of peptaibols identified in a sample [63].

The increase in the number of *Trichoderma* genomes, due to advances in new-generation sequencing (NGS), has allowed an advance in the characterization of new secondary metabolites via genome mining [10,57,60,64,65,66]. However, chemical and functional characterization based on knockout gene or heterologous expression of peptaibols in complete genomes are still scarce and difficult to predict *in silico*. Recently, the characterization of peptaibols linked to their respective peptaibol synthetases was performed in the clade *Longibrachiatum* and in species of the genus *Tolypocladium* [57,67]. Studies like these allow the prediction of peptaibols to move into a new era.

## 5. Conclusions

This is the first study on peptaibols biosynthesized using the species *Trichoderma endophyticum*. Twenty-one new sequences of peptaibols, named here as endophytins A1–A13 and B1–B8, consisting of 15-res were characterized in this work. The unpublished endophytins differ from each other by the exchange of AA in positions 5 (Aib/Vxx), 7 (Aib/Vxx) and 8 (Vxx/Lxx) and were characterized by the lack of the Aib-Pro motif that is widely found in *Trichoderma* species. In addition to these new sequences, other sequences of peptaibols from the harzianin HC class and the hypumuricin class were also characterized. The two main mechanisms used by *Trichoderma* in the diversification of peptaibols, which includes the loss and jumps of modules, were identified in this work. Our findings reinforce the urgency to seek new metabolites from different Brazilian biomes in order to provide new chemical entities that may be useful in medicine and agriculture.

## Figures and Tables

**Table 1:** New endophytins A1–A13 and B1–B8 produced by *T. endophyticum* MMSRG85.

Ac-Aib-Ala-Aib-Ala-AA_1_-Gln-AA_2_-AA_3_-Aib-Ala-Aib-Aib-AA_4_-AA_5_-AA_6_ol									
Peptaibols	Compound ^a^	Exp. *m*/*z* Δ*_m/z_* (in ppm)	Chemical Formula	AA_1_	AA_2_	AA_3_	AA_4_	AA_5_	AA_6_
Endophytins Series A									
Endophytin A1	1	1394.8370 (−0.07)	C_63_H_111_N_17_O_18_	Ala	Aib	Lxx	Ala	Gln	Lxxol
Endophytin A2	2	1394.8322 (−3.51)	C_63_H_111_N_17_O_18_	Aib	Aib	Vxx	Ala	Gln	Lxxol
Endophytin A3	5	1408.8505 (1.56)	C_64_H_113_N_17_O_18_	Aib	Aib	Vxx	Aib	Gln	Lxxol
Endophytin A4	7	1408.8424 (1.06)	C_64_H_113_N_17_O_18_	Aib	Vxx	Vxx	Ala	Gln	Lxxol
Endophytin A5	8	1422.8657 (1.89)	C_65_H_115_N_17_O_18_	Aib	Aib	Lxx	Aib	Gln	Lxxol
Endophytin A6	12	1422.8665 (1.56)	C_64_H_112_N_16_O_19_	Vxx	Aib	Vxx	Ala	Gln	Lxxol
Endophytin	11	1409.8345 (1.33)	C_65_H_115_N_17_O_18_	Aib	Aib	Vxx	Aib	Glu	Lxxol
Endophytin A8	14	1436.8805 (2.43)	C_66_H_117_N_17_O_18_	Aib	Vxx	Lxx	Aib	Gln	Lxxol
Endophytin A9	22	1436.8827 (8.28)	C_65_H_114_N_16_O_19_	Vxx	Vxx	Lxx	Ala	Gln	Lxxol
Endophytin A10	15	1423.8642 (0.07)	C_65_H_114_N_16_O_19_	Vxx	Vxx	Vxx	Ala	Glu	Lxxol
Endophytin A11	19	1423.8525 (0.90)	C_66_H_117_N_17_O_18_	Aib	Vxx	Vxx	Aib	Glu	Lxxol
Endophytin A12	23	1450.8867 (−1.51)	C_67_H_119_N_17_O_18_	Vxx	Vxx	Lxx	Aib	Gln	Lxxol
Endophytin A13	24	1437.8672 (0.55)	C_66_H_116_N_16_O_19_	Vxx	Aib	Lxx	Aib	Glu	Lxxol
Endophytins series B									
Endophytin B1	25	1484.8862 (1.48)	C_70_H_117_N_17_O_18_	Vxx	Vxx	Lxx	Aib	Gln	Pheol
Endophytin B2	26	1471.8369 (3.05)	C_69_H_114_N_16_O_19_	Vxx	Aib	Lxx	Aib	Glu	Pheol
Endophytin B3	6	1442.8390 (6.72)	C_67_H_111_N_17_O_18_	Aib	Aib	Vxx	Aib	Gln	Pheol
Endophytin B4	10	1456.8501 (1.78)	C_68_H_113_N_17_O_18_	Aib	Aib	Lxx	Aib	Gln	Pheol
Endophytin B5	18	1456.8505 (2.37)	C_69_H_115_N_17_O_18_	Vxx	Vxx	Vxx	Ala	Gln	Pheol
Endophytin B6	17	1470.8676 (−1.51)	C_68_H_113_N_17_O_18_	Aib	Vxx	Vxx	Aib	Gln	Pheol
Endophytin B7	20	1470.8673 (0.74)	C_69_H_115_N_17_O_18_	Vxx	Vxx	Vxx	Aib	Gln	Pheol
Endophytin B8	27	1485.8639 (−2.75)	C_70_H_116_N_16_O_19_	Vxx	Vxx	Lxx	Aib	Glu	Pheol

^a^ Numbering based in the elution order of the total ion chromatogram present in the supplementary section.

## Data Availability

The datasets generated during and/or analysed during the current study are available from the corresponding author on reasonable request. Data is not publicly available due to privacy.

## Supplementary Materials

The following supporting information can be downloaded at https://www.mdpi.com/article/10.3390/metabo13020221/s1. Table S1: Dataset of the adenylation domains of *Trichoderma* species used in the prediction of NRPS modules; Table S2: Putative BGCs found in the genome of *T. endophyticum* MMSRG85; Figure S1: Synteny analysis between BGC 8.3 of *Trichoderma endophyticum* strain MMSRG85 and the BGC related to the NRPS2 gene containing an NRPS that performs the biosynthesis of 14mer and 11mer peptaibols in *Trichoderma virens* Tv29-8; Figure S2: Phylogeny of 14-res NRPS adenylation modules; Table S3: LC-MS data of peptaibols annotated in *Trichoderma endophyticum* extract MMSRG85. Identified peptaibol sequences; Figure S3: Total ion chromatogram of the annotated compounds. Numbers indicate compounds annotated in ascending order of retention time (RT); Figure S4: Mirror match of peptaibol harzianin HC XIII (black color spectrum), annotated by means of similarity with the MS/MS spectrum present in the GNPS spectral library (green spectrum); Figure S5: MS (A) and MS/MS (B) spectra of compound 4; Figure S6: MS (A) and MS/MS (B) spectra of compound 13; Figure S7: MS (A) and MS/MS (B) spectra of compound 9; Figure S8: MS (A) and MS/MS (B) spectra of compound 3; Figure S9: MS (A) and MS/MS (B) spectra of compound 16; Figure S10: MS (A) and MS/MS (B) spectra of compound 21; Figure S11: MS (A) and MS/MS (B) spectra of endophytin A1; Figure S12: MS (A) and MS/MS (B) spectra of endophytin A3; Figure S13: MS (A) and MS/MS (B) spectra of endophytin A4; Figure S14: MS (A) and MS/MS (B) spectra of endophytin A5; Figure S15: MS (A) and MS/MS (B) spectra of endophytin A6; Figure S16: MS (A) and MS/MS (B) spectra of endophytin A7; Figure S17: MS (A) and MS/MS (B) spectra of endophytin A8; Figure S18: MS (A) and MS/MS (B) spectra of endophytin A9; Figure S19: MS (A) and MS/MS (B) spectra of endophytin A10; Figure S20: MS (A) and MS/MS (B) spectra of endophytin A11; Figure S21: MS (A) and MS/MS (B) spectra of endophytin A12; Figure S22: MS (A) and MS/MS (B) spectra of endophytin A13; Figure S23: MS (A) and MS/MS (B) spectra of endophytin B1; Figure S24: MS (A) and MS/MS (B) spectra of endophytin B2; Figure S25: MS (A) and MS/MS (B) spectra of endophytin B3; Figure S26: MS (A) and MS/MS (B) spectra of endophytin B4; Figure S27: MS (A) and MS/MS (B) spectra of endophytin B5; Figure S28: MS (A) and MS/MS (B) spectra of endophytin B6; Figure S29: MS (A) and MS/MS (B) spectra of endophytin B7; Figure S30: MS (A) and MS/MS (B) spectra of endophytin B8.
