# Supplementary material for: Characterization of Peptaibols Produced by a Marine Strain of the Fungus Trichoderma endophyticum via Mass Spectrometry, Genome Mining and Phylogeny-Based Prediction
Source: Metabolites. 2023 Feb 3;13(2):221. doi: 10.3390/metabo13020221 (PMC9961477; doi:10.3390/metabo13020221)

Supplementary Information

Characterization of peptaibols produced by a marine strain of the fungus *Trichoderma endophyticum* guided by mass spectrometry, genome mining and phylogeny-based prediction

Gleucinei S. Castro[1″], Thiago F. Sousa[2″], Gilvan F. da Silva[3], Rita C. N. Pedroso[4], Kelly S. Menezes,[1] Marcos A. Soares[5], Gustavo M. Dias[6], Aline O. Santos[1], Michel E. B. Yamagishi[7], Jéssica V. Faria[8], Ana H. Januário[4], Hector H. F. Koolen[1*].

[1]*Grupo de Pesquisas em Metabolômica e Espectrometria de Massas, Universidade do Estado do Amazonas (UEA), 690065-130 Manaus, Brazil*
[2]*Programa de Pós-graduação em Biotecnologia, Universidade Federal do Amazonas (UFAM), Manaus 69080-900, Brazil*
[3]*Embrapa Amazônia Ocidental, 69010-970 Manaus, Brazil*
[4]*Núcleo de Pesquisa em Ciências Exatas e Tecnológicas, Universidade de Franca, 14404-600 Franca, Brazil*
[5]*Instituto de Biociências, Universidade Federal de Mato Grosso (UFMT), 78060-900, Cuiabá, Brazil*
[6]*Centro de Ciências Naturais e Humanas, Universidade Federal do ABC (UFABC), 09606-070 São Bernardo do Campo, Brazil*
[7]*Embrapa Digital, 13083-970 Campinas, Brazil*
[8]*Centro de Estudos Superiores de Tefé, Universidade do Estado do Amazonas (UEA), 69470-000 Tefé, Brazil*
Corresponding author: hkoolen@uea.edu.br ORCID: 0000-0002-0181-348X

Table S1. Dataset of the adenylation domains of *Trichoderma* species used in the prediction of NRPS modules.

>*Trichoderma_endophyticum-mod1*	LEQSLKANGEAPMIVNLCLHDLIERQAENRPNATAIQAWDLELTYLQLDCATNRLAHHLVKSCGVKDQDLVH<br>VCFEKSAWFFVSIIAVNKAGATWVPLDPSHPLQRQQQVVSQTKATLALASASNVDMCSKLVANVVEVSHALDE<br>KLSKTEASSYGPISNVSPRNAAYILFTSGSTGTPKGLVMQHGAVCTSQTAIAKRLGMTSNVRMLQFASFVFDLSIGE<br>IVGPWVAGGCLCVPSEETRMNTGSLVDFINAMKITWAYLTPSFTRTLNPDDIPGLELLLFAGEAVGRDVFEAWFG<br>KVRLINGWGPAETCVFSTLHEWKGLEESPLTIGRPVGGYCWIVDPQDPQRLAPVGTLGEVVIQGPTILREYLADFA<br>KTEASIIRNLPEWVPNRTANHWDRFYKSGDLCRYNADGTIEFGSRKDNQVKIRGLRVELGEIEHHFRESLEGVKQ<br>VAVDVVNGDGGAIIVSYFCFTGETRTAGRNSETNVKDILAPMTSELQSQLNALVGQLSVTLPRYMIPTLFI
>*Trichoderma _endophyticum-mod10*	ELAQKSNGVGPEIINDCIHRIIARQVERRPNAPAIDAWDGQFTYGELDRASNRLAHLLINDYGVNIGDIVHVCFE<br>KSKWYLVAILAINKAGAAWAPFDPANPLQRLQAVAGRTGAKLALASTANISLCEQVVESVVEVSSAFDGELLNS<br>YGISEKGPDVNVTPTDAAYILFTSGSTGMPKGIVMQHRALCTNQTALSRWLGIDHTVRTLQFSSFVFDVSVGEIIQS<br>LMNGACVCVPSDHMRLNSLDTFVRDFNVTWAYLTPSFIRTLKPKNFPSLKMVLLAGEPTPQDVLSTWFGLPNTRI<br>INAWGPAETCVYSTLYEWQSNTDSLSTIGHAVGAYIWVVNVENPQQLAPTGCLGEIVVQGPSLLKEYLADPEKT<br>NAAIMTELPGWAPQRQSPAWSRFYKTGDLGFYDYNGILHYAGRKDTQVKIRGLRVELGEVEYHIQNSLIGIRQVA<br>VDVFKTERGANLVAYFCFSDDTKTPNQNTDAEGKDVFMSIDAELQANLVEMLAQLNSSLPSYMVPTLFI
>*Trichoderma _endophyticum-mod11*	VQKAMEINSEIPEVIDACMHELFEKQVNLSPQAPAIAAWDGNFTYSELNRAANRLAHYLINVYSVKPHDFVHLC<br>FDKSAWYQVSIIAVNKAGAAWVPLDPSHPEQRLRSIVSQTKATLALVSPDNVALCSGLVDNVFEVTSALDDSML<br>ATEETLVSPEVSISSRAVSYALFTSGSTGTPKGFMIEHGALCTNANAIAKRVRHGSNARVLQFAAYVFDYCIVEIIM<br>TLLQGACICVPSEHIRMNSLIDFIRDMNINCLYLTPSFLRTIHPSKVPSVELVFVAGEALPRDVFETWVGRVRFLNG<br>WGPAETVVFSSIHEFTSVDESPLTIGRPVGGFCWIVDPNNPQRLAPIGTLGEVVIQGPTLLREYLGDPQKTQETIING<br>LLEWAPRPDSYNWNRFYKTGDLCFYNTNGMIEFSSRKDTQVKIRGLRVELGEIEYHIQSLLKSIRQIAVDVLKTDN<br>GSNLVAYLCFNDDIRQLHEADINGPFAPFDAEFQETLVGAVALSQSELNKFSIHGVSKRMP
>*Trichoderma _endophyticum-mod12*	DLEQAKRWNVEDPEILDTCIHSLVEKQARIRPEAPAICAWDGQMSYNQLNDAANGLAHHLLKAGVKTNDLVH<br>VCFEKSVWFFVSILAINKVGAAWVPLDPSHPEQRLRQVVGQTLAKLALSSPANALLCSKLVDHVIEVSPGLIDEIFI<br>IYHGLSNPAANVSPDSAAYVLFTSGSTGTPKGFVMQHSAVCTSQAAIAKRLGLTPDVRILQFAAYVFDLSIGEIVA<br>PLINGACVCVPSEETRMNGLKDFILDTNISWAFLTPSFVRTLKPEDVPGLKLLLLAGEAVGRDILDAWFGKVRLV<br>NGWGPAETCVFSTLHEWSSIDESPLTIGRPVGGRCWIVDAEDSNKLTPIGCLGEIVLQGPTLLREYLADPQRSKESII<br>TVLPSWVPKKDSNSQSWSRFYKSGDLCFYNPDGTLEFYSRKDTQVKIRGLRVELGEVRYHIQQALPAARQVAVDV<br>YKGENSTNLVAYFCFSDETRTAAISSDASESLFLPINEDLQSHLATVAGQLRISLPQYMIPTMF
>*Trichoderma _endophyticum-mod13*	DLHRATEFNSEFPEIMDTCIHHLIELRAKEAPDSPAIWAWDGELTYGQLNEAANKLAHYLINKYNVQVEDLIHV<br>CFEKSVWYWVAIFAINKAGAVWVPLDPSHPEQRLRQVTSQTRSKLALISNDTRSLISGIIDRVVEVSPTLYKQINSSL<br>GANDPQIPVSSHNAAYVLFTSGSTGVPKGLVMTHGSLTTSQTAIRKRLGISLSNRTLQFASHVFDLCIGESILQLISG<br>ACMFIPSEHTRMNGLKEFIAEHEINTLWLTPSFVRTLNPDQIPSVTLLVLAGEAVSRDILTTWFGRVRLWSGWGPA<br>ETCVLSSLHEFQSVDESPLTIGRPVGGFCWVVDPTNPDKLAPIGTIGEVVIQGPTILREYLADIERTKATTMYELPA<br>WAPYRDQPSWSRFFKSGDLASYNTDGTLEFSSRKDTQVKIRGLRVELGEIEYHVRNGLEGARQVAVDVFTTDSGT<br>RLIVYFCFSDEMRTANISESDEDIFLPVTEELQTQLSKLISQLNVTLPRYMVPSLFVPC
>*Trichoderma _endophyticum-mod14*	PWDAQQAAQLNSKKPDFIHTCLHDIFAEHASNSPHHEAVYSSEGSLTYGELDHLTDIVATHLSSLNVGPETVVPF<br>CFEKSMWAVVAILAILKAGAAFVPLDPSHPISRRETLVREVNASVLIASTKAITSCAGMADHIVELSSSVISKLASSIT<br>PKVLPKVGPRNTAYVLFTSGSTGKPKGVVMQHGSFSSTTIGYGKVYNLSPLSRIFQFSNYIFDGSLGEIFGPLAFGGT<br>ICIPSDDERLQCAPDFMHKANVNTAMLTPSFVRTFTPDKVPYLKTLVLGGEAASKSILEMWVDRVTLFNGYGPAE<br>ACNYATAHMFKSSAESPRIIGSSFNGGCWIVEPDNHNKLAPIGCIGELVLQGHALARGYLNDKVRTEKSFVSEIGS<br>LPSSLQHEPKRFYLTGDLVRYNADGELEYLGRKDSQVKLRGQRLELGEIEYTITQSLESVHHVAVDVLHGQAGDA<br>LIAFISFTGDATAEWVTDNLRLNLLVSDDAMRSVLGSLRESLEATLPGYMVPSIILPLH
>*Trichoderma _endophyticum-mod15*	WDLQKQLEWNDEIYNSADTTLHDLFSKQAARRPDHEALYSSEGSMTYSELDQLTTQLAVYLSRLGVRPETIVPFC<br>FEKSMWTIVVMLGILKAGGVFIPLDPSHPTSRRQALIDEVCAQFLVVSPTTAPDCQDMVKNTVEFSPSLISSISAIDT<br>DEQLYVTPILNNAAYVLFTSGSTGKPKGVIIEHNAISAALLRQSDAFSIHAGSRMLQFANYVFDACITEIFTSLIVGA<br>TVCVPTEDERMNNTAAFIREARINNLFLTPTFLKTLSPSQIPGVKTILTGGEAPSREIIETWVDEVDLHNAYGPAEG<br>CVASTNYTYSSSITVSATTLGRSFAHGLWVVDPDNHDQLMPIGCVGELLLQGSSLARGYINDEKKTRQSFIEEVKW<br>LPSNVNVGERRFYKTGDLVRYVSDGSIEYLGRKDAQVKIRGQRVELGEIEYHIKSSDTVIEHAVVDITRKDGRESLI<br>AFICFNSQQEAESASKEVHLTELTDGLREMFSDIATSISSALPSHMIPKYFIPVEHM
>*Trichoderma _endophyticum-mod2*	GKREVQHAADASRFRPATESCTHWEIYKQIEANPDAPAFDSWDAKLTYREVGVLASRLAAKLQNLGVGPDVLV<br>PISFPKSTPAMITMVAIQMAGGAIVPLDPAAPLARLQSIVSDTQAKLIVTHPSLQAKIQELGVNLLIVDLSILDQLPN<br>PSSKYVSSGTTPENLYAVLFTSGSTGKPKGIRIPHSSLCSVADAHAADTGVGPGSRVFQFSAYTFDIGILDVLVTLMR<br>GGCVCVPSDHDRLNNLAGAINATQANWVFLTPTVADMLNPADVPCLTTVNLGGEAVSKKTAERWEGYATLN<br>GLYGPAEASICARNSDVNNGSSTNLGFPLSSAFWAVEPNDPSRLVPIGCIGELLIQSPILAYGYLNVDAKVAANWL<br>ENMTYDWLPADGPKRAYLTGDLVRRNPDGTFEYMGRKDNQVKIRGQRVELSEIEYTIQTNLPDIKQVMVDVIN<br>NNEAGLSLIAFLCFSDDTKGSNEGLEDIFLTTTPEMKQQFTTMLSHLHISLPQYMIPTIFVPCKFM
>*Trichoderma _endophyticum-mod4*	FKLSDVSVASSWDLDLAEASNGDGPEIVDDCAHLMIERQTGLTPHAYAVHAWDGTLTYINSHGVKVGDIIHVCF<br>EKSLWYVVSVLAINKAGAAWVPMDPAHPFQRLQQVASQTGAKIALASSIHRPLCSKLLDTVIEVSSALDEQLHSD<br>PDINDIKPAVTVASNDVAYLLFTSGSTGVPKGIIMEHGSLCTSQRDIAKRLDLKSSVRMLQFSSFVFDVSVGEIMLSL<br>MHGGCVCIPSDHDRLNNLAKFIRDTEVSWAFLTPSFARTLRPEHVPSLELIVLAGEPVSQDVFDLWFGKTRLVNG<br>WGPAETCVLSAIHEWKSADESPLTIGRSVGSFAWIVDVENPRRLAPVGCIGEIVMQGPTLLREYLADPAKTRSSAL<br>TSLPNWAPRSNDRNWGRFYKTGDLGIYNPDGTIHYSGRKDTQVKIRGLRVELGEVEHHIRNSLDVIQQIAVDVLR<br>TETGANLVSYICFSSETKTPGPDTDPEGTDIFLSMTESIQNELNIVINKLNILLPRYMIPTYFI
>*Trichoderma _endophyticum-mod3*	EQSLNANNEVPDIIDSCLHELIEQKAIEQPGSLAIVGWDRTFTYAELNEAANRLAHHLTQSFAIKFDELIHVCFEKS<br>AWHFVAILAVNKAGAGWVPLDPSHPEQRLRQIVSQTRAKIVLCSPSNADVCNGLGLSIIEISPFFDQNLIKSGITGT<br>TGPAVKVAPHNIAYVLFTSGSTGTPKGLVMEHRSVCTSQNAISLRLGLTANVRMLQFAAFVFDLSIGEIVAPLISG<br>ASLYIPEENTRLNNLSNFIQDNKINWAFLTPAFARTIRPEDVPALELLLLAGEAVGRDVFERWFGKVRLINGWGP<br>AETCVFSTLHEWQSIDESPLTVGRPVGGFCWVVDPENPQKLAPIGTTGEVVIQGPTLLREYLADSERTKLSILYDLP<br>TWAPRRESQNWNRFYKSGDLCCYNPDGTIEFLTRKDTQIKIRGLRVELGEIEHHLQLALDDICQIAVDVLRGENG<br>SSLVAYFCFNGMSKTADACVASEEKGPFMSIDEALQTRLIAASGELRVALPLYMVPTFFI
>*Trichoderma _endophyticum-mod5*	DLDRSISFNSEISEIVNACVHELIERQAERRPDAVAISAWDAEFTYNQLNCAANRLANYIITSYGIKPNDIIHVCFEK<br>SIWHFVAILAINKAGAAWAPLDPSQPEQRLRRIVDQTRARLILTSPNNCDLCSTLVENVLSLTPNLDRELAETTDS<br>EAPDVVVTPDNAAYILFTSGSTGTPKGFVMEHRSVCTSQTAMIKRLGLTSDVRMLQFASFVFDMSIGEAIPTLISGG<br>CICIPSEETRMNNITQYMRDRQVNWAFFTPSFLRTLAPKDIPTLEVLLLAGEAVPKEILNTWFGKVRLINGWGPAE<br>TCVCSTWHEWKSVDESPLTIGRPVGGFCWIVDPEDPYKLAPVGTLGEVVIQGPTLLREYLADPERTAASCVKAPK<br>WAPQPDSQHWGRFYKSGDLCSYNADGTLEFSSRKDTQIKIRGLRVELGEVEYHIQAALQDVRQIAVDVYNNERG<br>THLVAYICFADEIRVAGVNRDTSSPFFSIDQKLQSRLNALVGELGVTLPRYMIPTLYIPCS
>*Trichoderma _endophyticum-mod7*	IEYAIRQNNEVPEIIESCFHTLVEQQKMIRPDSPAVYSWDGNFTYSQLDQAANRLANHLVTEYGIKNDELIHVCFE<br>KSSWFVVAILAINKAGGAWVPLDPSHPTQRHQQVVGQTKARLALVSRSNISTCVDLVEHVVEVSSTTDEILNKTE<br>LSDRGPDREIPFSNAAYVLFTSGSTGTPKGLVMEHRSFCTAQTAITKRLRLSSSARTLQFASYVFDAAVAEIAATLIT<br>GGCLCIPSDHDRMNTLSEFIRQNGINWAWLTPSLIRTLKPKDVSSLETVILVGEAVTRDIMDVWFGKIRLINGWGP<br>AETCVVSTLHEWQSIDESPLTIGSPVGGFCWIVDPENPYKIAPVGAVGEIVIQGPTILREYLADPVRTSASIVKDLPA<br>WALNRKAKYWNRFYKSGDLGLYNANGTIQFVSRKDTQIKIRGLRVELGEVEHYVKIAFSGVQQVAVDVIRSGIG<br>ASLVAYFCFTDEIRITGTRAGSDDSGPLVQMDDELQGTLTTVIGQLNISLPRYMVPTFFI
>*Trichoderma _endophyticum-mod6*	DLQQAISYNVKEPSMVSACVHELITQQALRDPHHEAIYSNEGTMTYATLDRLSSLLAQYLHGLGVRPESVVPFCF<br>DKSSWAIVAMLAILKAGGVFLPLDPSHPRNRREALIREVGAEIMVVSPSSSVPCENLTSIMVELTIQLLEELSSIYDA<br>FQEIRPKAKPSNAAYVLFTSGSTGKPKGVLMEHSAFATSTLGHGRVYNLSPSSRVFQFSNYIFDGSLGEIFTTLSFGG<br>TVCVPSDDERLQGAPSFMREARVNTAMLTPSFVRTFTPEQVPSLQLLVLGGEPSSKDLLETWCDRLRLVNGYGPA<br>EACNYATTHDFKITDTPRIIGRGFNSACWIVDPTDYNKLTPIGCVGELVIQGNALARGYINDADRTAASFINHLDC<br>LHRSTIPGPYRFYLTGDLVRYNVNGEMEYLGRKDTQVKLRGQRLELGEIEYHVKRSLSNIEHVAVDVVHREAGD<br>ALIAFISFKEKMASSGNILLLNDDLRLALATIMEHLKMSLPGYMVPTSILPVKRMPFITS
>*Trichoderma _endophyticum-mod8*	ALTSKGDMLLGSISLSSRWDFDQALQLNSRTPDAVERCFHEMVDEVASVRKDSLAISGWDKSFTYKEMTETTNRI<br>AQYLVAEHGVKVGDIIHVCFEKSAWFLIATIAINKAGAAWSTLDPSHPTERYQKIISQTVIELTPELDAKLALDSQ<br>WSAERPAVKITPSDAAYILFTSGSTGVPKGVVIEHASLCTSQTSLSEALKFQEDFRVLQFSSYSFDAALFEIGSTFLTG<br>ACLFVPSWDEQMNQLTEYIRKHQITCALLTPTLARTIRPEEVPSIDMLLLGGEAPTRDILDIWFGKVRLINGWGPTE<br>CCVIACLHEWTSVDESPKVIGRPIGGSCWIVDPDDATRMAPSGTVGEIVVQGRNLLREYLSDPVKTAAATVTGLP

>Trichoderma _endophyticum-mod9

QWAPKRDSLHWDRFYLTGDLGFINDSGNIEYCTRKDTQVKIRGQRLELGEIEHHIQAHLEGVRQVAVDIVKSEA
GSTLIAFISFSDAKEPTSLDEKTIGNGIFLPLEGSLQATISSLMGTLGTLMPRYMVPSAFI
LANSMRQNSDIPDIVDSCIHQLIEHQATAQPDALAIVSWDRDFTYRQLNEASNRLAHFLVDKYNVKPEDLIPVCF
EKSAWYFVAITAINKAGAAWVPLDPSHPELRLRQVVSQTRAKLALSSSANSKLCGILVGEVVEVNAELDNKLLAT
EASANAPIVAISPRNAAYVLFTSGSTGTPKGLVMEHGSVCTSQVAIAKRLGLNSKVRILQFAAFVFDLSIGEIIGPLIS
GACICVPSEDTRMSGIVDFINTKAVTWAYLTPSFVRTIKPSEVSQLELLLLAGEAVPRDIFTTWFGKLRLINGWGPA
ETCCFSSLHEWQSAEESPLTVGRPVGSFCWIVDPENPCQLAPTGILGEVVIQGPTILREYLSDKERTDAAVVMSLPE
WAPFREQANWSRFYKSGDLCKYNPDGTIEFSSRKDTQVKIRGLRVELGEVEHAVQIALDGVQQIAVDIFKGDNG
TNLVAYFSFSDETRQIHEEDPSGPFQPMDENLQARLTSVVGELNIALPRYMIPTLFIPCK

>Trichoderma longibrachiatum_mod1_Aib

ESQAKTNPDAPAIHAWDMELSYSQLDRAANRLAHHLVKSCGVKDQDFVHVCFEKSAWFFVSVIAVNKAGATW
VPLDPSHPLQRQQQVVSQTKATLALASPSNVEMCLELVANVVEVSAAMDEKLSKTEESSYGPIRDVSPNNAAYV
LFTSGSTGTPKGLVMQHRAVCTSQTAIAKRLGMVSSVRMLQFASFVFDLSIGEIVGPWVVGGCLCVPSEETRMNN
LVDFINTMQVNWAYLTPSFTRTLNPDDVPGLELLLFAGEAVGRDVFEAWFGKVRLINGWGPAETCVFSTLHEW
KSLEESPLTVGKPVGGYCWIVDPHDPQLLAPVGTLGEVVIQGPTVLREYLADPTKTEASLVRNLPEWTPNRTAAH
WDRFYKSGDLCRYNADGTIEFGSRKDSQVKI

>Trichoderma longibrachiatum_mod10_Aib

EKQAATQPDAPAVISWDRDFTYKQLNEASNRLAHLLVNKYQVKPNDLVHVCFDKCAWHFVAITAINKVGAA
WVPLDPSHPEMRLRQIVSQTKATLALVSSSNATLCSSLVEIVIEVNEKLDNELLAAEGGEYGPSVDFSSRSAAYVLF
TSGSTGVPKGFVMEHASVCTSQVAIARRLGLGPNVRMLQFAAFVFDLSIGEIIGPLVSGACICVPSEHTRMNGIIDF
INEKKVTWAYLTPSFARTIRPSEVPYLELLLLAGEAVPREVFTTWFGSNVRLVNGWGPAETCVFSTLHEWKSADES
PLTVGRPVGGFCWISDPEDPSRLAATGTLGEILIQGPTILREYLSDVERTKLAVVKSLPEWAPFRNKPGWNRMYKS
GDLGFYNPDGTIEFSSRKDTQVKI

>Trichoderma longibrachiatum_mod11_Gly

ERQVKKRPDAPAIDAWDGRLTYSQLDHAANRLAHLLVDDYAVQVGDIVHVSFEKSMWYIVAILAINKAGAAW
APLDSAHPFQRLQAVAKQTGAKLALASTANAGLCGQLVGRVIEVSAAIDAELSASDVNSENGPQVNVTPLDASY
ILFTSGSTGTPKGIVMQHGALCTSQTDISRWLGLDHTVRMLQFSSFVFDVSVGEIMEPLMNGACVCVPSDHMRLN
SLDSFVRDFNVTWAYLTPSFTRTLKPQDFPGLELLLLIGEAVTQDVLDTWYGLPNTRFVNAWGPAETCVFSTLYD
WQSNTESPLTIGRAVGAYVWVVDAENPQRLAPTGCLGEIVVQGPPLLREYLADPTKTAAATVTELPEWAPRRES
TKWNRFYRTGDLGFYSHDGLLHYASRKDTQVKI

>Trichoderma longibrachiatum_mod12_Lxx

EKQASLIPQAPAIAAWDGNFTYDELNKAANKLAHHLVNVYGVKPNDFVHVCFEKSSWYIVSIIAINKAGATWV
PLDPFHPEQRLQSIISQTKATLALVSPSNVALLSPLVANVVAVDSSLDNMLPVQDEAQSPSVSVSPSTAAYVLFTSG
STGTPKGVIIEHRSLCTSQKDIAKRLRYHEKVRVLQFSAFVFDFSISEIIMSLLHGACVCVPSEHIRLNSIVDFIRDMEI
NWLYLTPSFLRTIDPNDVPNVELVLMGGEAVPREVFETWYGRVRLFNAWGPTESCVMGTIHEFESADESPLTIGR
PVGGFCWIVDPENPQLLAPTGTLGEVVIQGPTLLREYLDNPEKTAEATVYELPDWVPRPDENNWNRFYKTGDLC
FYNPNGTIEFSSRKDTQVKI

>Trichoderma longibrachiatum_mod13_Aib

EEQARIRPDAPAICAWDGEMSYLQLNAAANKLAHHILNAGVKSEDLVHVCFEKSLWFFVSIVAINKVGAAWVP
LDSSHPEQRLRQVVSQTRAKFALSSPTNAALCGSLVENIIEVSQSLLDSIPYDGANGPAFNVPSGSAAYVLFTSGST
GTPKGLVMQHQAVCTSQTAIAKRLRLTPDVRILQFAAYVFDLSIGEIVAPLIHGACVCVPSEEMRMNGLKEFVRD
ASINWAFLTPSFVRTLRPEDVPGLDLLLLAGEAVGRDILDTWFGKVRLVNGWGPAETCVFSTLHEWVSVEESPLT
VGTPVGGHCWIVDAQDSSKLAPIGCLGEVVLQGPTLLREYLADPQRSKEAIITSLPSWAPKQDSQTWSRFYKSGD
LCHYNPDGTIEFYSRKDTQVKI

>Trichoderma longibrachiatum_mod14_Pro

QERIEKQPTDPAIASWDGDLTYEELGVLASRLAWKLQGLGVGPESLIPLCFPKSTWAVVAMVAIEMAGGAFVPL
DPKAPVARLRGIIEDTKSTLAVASPSCQETLQEVGIDVLVVDEALLLELSDPVEKIQSKIRPKNASVVLFTSGSTGKP
KGMVIQHDSLCSSGNAYGEDLNIGPGTRVFQFSAYTFDVGVLDCLVSLMRGATVCIPSDHARLNDLAGAMTATK
ANWVFLTPTVADLLSPTDVPYLKVLCLGGEAISKKCADRWVNYTELHGLYGPAEASICAWNPLVGKSGRSTNLG
RPISSAFWVVEPSNYKRLVPVGCIGELLIEGPMLARGYLNVSADVASNWMENVDWLPGSNKKRVYRTGDLVRR
NPDGTFDFMGRKDTQVKL

>Trichoderma longibrachiatum_mod15_Vxx

EQRAQMNPDAEAVSAWDASFTYAELNRSANILANYLIQSMGVRVGDLVHVCFEKSAWYVVSILAINKAGAAWI
PLDPSHPVERHQQVVGQTRSKLALTSPTNAAKCAKLVNNVLEVTRELLDDLETKIKDTKPTTTVTPQDVAYILFTS
GSTGVPKGVVMEHGALCSSQTSISKRLGYASGVRMLQFASFVFDACIGEIIAPLISGSCVCIPSWETQMNSLTSYICE
ENVTWAMLTPSFARTIDPSEVPCLELLILIGEAVSRDVFELWFGKLRLLNGWGPTETCVFGALHEWQSIDESQMTI
GQPVGGYCWIVDPEDPQKLAPTGTFGEVVIQGPNLLREYLADEAKTASSTVAVLPEWAPNRHSRYWNRFYKTG
DLAMYNPDGTIQYYSRKDTQVKI

>Trichoderma longibrachiatum_mod16_Aib

ERQAEIRPDAMAIRAWDAEFTYNEFNRAANRLANHLTASYNIKPDDLIHVCFEKSAWFFVSILAINKSGAAWVP
LDPSHPEQRLRQVVSQTRARLALTSPTNRDLVAGLVESVVTVDSQLDAELSKIDEYSQKGPVTTVSSDNAVYVLFT
SGSTGTPKGLVMQHGSVATSQTAIVKRLGLTPDVRMLQFAAFVFDLSIGEIIAPLITGACLCIPSDHTRMNGLTRYI
RDMGINWAFLTPSFIRTIKPAEVPGLDLVLLAGEAVPRDVLTTWFGKVRLINGWGPAETCVFSTLHEWQSVNESP
LTVGKPVGGFCWVVDPEDPHRLAPTGTLGEVVIQGPTLLREYLSDPQRTQASTVYALPKWAPRPDSKHWNKFYK
SGDLCYYNQDGTIEFSTRKDTQIKI

>Trichoderma longibrachiatum_mod17_Aib

EAQARQTPDLPAVWAWDGHLTYGQLNEAANRLAHYLINEHNVKIEDLIHVCFEKSVWHWVSVLAINKAGAV
WVPLDPSHPEQRLRQVASQTRSTLALTSDTTSSLLSGFIDNVFEVSPSLFDQIDVRLGEKEPQVSVSSSNAAYVLFTS
GSTGTPKGLVMTHGAFATSQIAIKKRIGTDTNTRALQFASYVFDMSVGEGFVQLISGACIFIPSEHTRMNGLKEFIA
EHEINSLWLTPSFIRTLSPEQVPTVDVVFLAGEAIPRDVFTTWCTKVRLWNGWGPAETCVVSSLHEFTSLDESPLTI
GRPIGGYCWIVDPTDHTRLAPIGTIGEVVIQSPTILREYLADVERTKASTVYELPAWAPYRDQTPWSRFFKSGDLAS
YNPDGTLEFASRKDTQVKI

>Trichoderma longibrachiatum_mod18_Gly

SQHASSSPHHEAIFSSEGSLTYGELDHLTDILATHLSSLGAGPETVVPFCFEKSMWAVVAIVSILKAGAAFVPLDPS
HPTSRREALVKEVRARVLVASSSAITSCKGMAEHLVELSPSVMAKLAASVTPRILPKVGPRNTAYILFTSGSTGKPK
GVVMQHGSFSSTTIGYGKVYNLSQLSRVFQFSNYIFDGSLGEIFGPLAFGGTICIPSEDERLGSAPAFMNTAKVNTA
MLTPSFVRTFTPDQVPHLKTLVLGGEAASKSTLEMWVDRVTLYNGYGPAEACNYAATHVFKSSSESPRIIGSGFN
GACWVVEPDNHSILTPIGCTGELVLQGHALARGYLNDKAKTEQSFVSDVGSVPLALLHEPKRFYLTGDLVRYNS
NGELEYLGRKDSQVKL

>Trichoderma longibrachiatum_mod19_Gly

DNAQRDPNHEAIFSSEGSMTYAALDRLTDVLASHLCQLGVGPETIVPFCFEKSMWAVVAIVGTLKAGGVFVPLD
PSHPINRREALVREVGAQIIIASESAAASCAGMAPRIVELSSDFMARLSAPSQTIVEPRRPSPSNAAYVLFTSGSTGK
PKGVVMEHSALTTSTIGYGRVYELSPASRVFQFSNYIFDGSLGEILATLTFGGTVCIPDEVERLQDAPGFVRKAQIN
TAMLTPSFVRTFTPDQVPGLKTLVLGGEPAGRDILDAWCDRVKLINGYGPAEACNYATWHPFSSSKDSPRVIGKA
FNSSCWVVEPHNHHVLTPVGCVGELAIQGHVLARGYINDSERTRSSFVTELASLSSVIAGPQRFYLTGDLVRYCSD
GSLEYLGRKDTQVKL

>Trichoderma longibrachiatum_mod2_Ala

KQIEAQPDAPAFDSWDAKLTYREVGHLATRLAAKLQSLGIGPDVMVPISFPKSTPAMITMVAIQMAGGALVPLD
PAAPLARLQSIVSDTQAKLIVTHPSLEEKLQELGVDLLIVDLSMLDKLPDPSTKFISSDATPESLYAVLFTSGSTGKP
KGIRIPHSSLCSVSDAHAAETGVGPGSRVFQFSAYTFDIGILDVLVTLMRGGCVCVPSDHDRLNN--
LAGVISALQANWVFLTPTVADMLNPTDVPCLTTINLGGEAVNKKSSERWQGYTTLNGLYGPAEASICARNADV
HNGASTNLGFPLSSAFWAVEPNDPSRLVPIGCIGELLIQSPILAYGYLNADAKNTANWLEDMTYNWLPANGPKR
AYRTGDLVRRNPDGTFEYMGRKDNQVKI

>Trichoderma longibrachiatum_mod20_Leol

SKQAARRPAHQALYSSEGSMTYSKLDRLTTQFAVYLSSLGVRPETIVPFCFEKSIWAIVAMMGILKAGGAFMPLDP
SYPASRRQALIDEVNAQFIIVSPTTAPDSQGMVRNTIELSPSLIAIVSTIDVTEQSFTKSGPNDAAYVLFTSGSTGKPK
GVIIDHKAISAALLRQHEAFSFNDDTRTLQFANFVFDACIAEIFTSLVVGATVCVPTEHERVHNTAAFIREACVNH
AFLTPTFIKTLSPEQVPDMKTVILMGEAPSQDIIDTWANEIDLHNGYGPAEGCVGSTNNTYSSSVKVSVTNVGRSF
THGLWIVDPDDHNRLMPIGCVGELLLQGSSLARGYINDEEKSRQAFIEGVEWLPSNINVGERRFYKTGDLVRYTP
DGSIEYVSRKDTQVKI

>Trichoderma longibrachiatum_mod3_aib

EEQALKRPDAPAVVGWDLTFTYAELNEAANRLAHYLTQNFAIKPDELIHVCFEKSAWHFVAILAINKAGAGWV
PLDPSHPEQRLRQIASQTRARIILTSPANSDICTRLGLSVIEISPFFDQKLVKSGMKSSAGPDVKVTPRNIAYVLFTSG
STGTPKGLVMEHGSVCTSQTAISQRLRLTADVRMLQFAAFVFDLSIGEIVAPLISGASLYIPDENTRLNDLSNFIQE
NRINWAFLTPAFARTIKPEDVPALDLLLLAGEAVGRDVFESWFGKVRLINGWGPAETCVFSTLHEWKSIDESPLT
VGRPVGGFCWIVDPENPDKLAPTGTIGEVVIQGPTLLREYLADAERTKMSTVSDLPSWAPHRESQYWSRFYKSGD
LCYYNPDGTIEFSTRKDTQIKI

>Trichoderma longibrachiatum_mod4_Ala

EHQTKQTPHAHAVHAWDGSLTYSELDLAANRLANFLIQRHDVKVGDVVHVCFEKSLWYVVSVLAINKAGAA
WVPMDPAHPFQRLQQVASQTGAKLALSSSIHGPLCSKLLDTVIEVSLSLDEQLKSDEAISDIKPGTKVTPNDAVYL
LFTSGSTGVPKGIIMEHASLCTSQRDIAKRLGLTSGVRMLQFSSFVFDVSVGEIMLSLMHGGCVCIPSDHDRLNNL
DGFIRDAEVTWAFLTPSFARTLRPQDVPTLELIVLAGEPVSQDVFDLWFGKARLVNGWGPAETCVLSAIHEWKSA
DESPLTIGRSVGSFAWIVDAENPHRLAPVGCIGEIVMQGPTLLREYLADPAKTAASTMTSLPNWAPRANDKKWA
RFYKTGDLGFYNPDGSIHYSGRKDTQVKI

>Trichoderma longibrachiatum_mod5_Aib

ARQAELRPDAVAISAWDAELTYSQLDLAANRLANHIITAYGVKPNDFIHVCFEKSAWHFVAILAINKAGAAWV
PLDPSHPEQRLRQVVSQTGAKLVLTSPGNSTLCSGLLDDVLEVKPGLDQKLAETVGSKAPKVAMTPNHAVYALF
TSGSTGTPKGLVMQHRAVCTSQTAIAKRLGLSSDIRQLQFAAFVFDLSIGEIIAPLISGACICVPSEDTRMNSLTEYIR
DQRINWAFLTPSFVRTLRPKDVPGLELLLLAGEAVSKEILHTWFGKLRLINGWGPAETCVFSTLHEWTSVNESPLN
VGKPVGGFCWVVDPENPQKLAPVGTLGEVVIQGPTLLREYLADPERTAASSAKTPEWAPQPDSKHWGHLYKSG
DLCSYNPDGTLEFSSRKDTQIKI

>Trichoderma longibrachiatum_mod6_ala

ERQAASTPEAIAIDSWDGKLTYAQLDQAATRLSHHLIKTYEVAPGDLILLLFEKSLWYVISTIAVNKAGAAWVPL
DPSHPMQRLQQVTRQTKAKVVLTSSLHIELGEELLDTVVDVGQALDDALATAGHPLHAPDVTTSSRDSAYVLFT
SGSTGVPKGLVMSHGSVCTSQTAISSRLGLHSGVRMLQFSAFVFDVSIGEIYGSLIRGACVVMPSDEIRLNNLTSFM
QDKEVNWACFTPSFAEILHPTDLPNMELIILAGEPSTKQILEDWVGKVKLINAWGPAESCVFSSMHEWKTATDSP
ITIGKPVATFAWIVDPENPHRLAPIGTVGEVVLQGPTLLCEYLHDPVKTETSILKSIPSWAPR-RES-
RHWNRFYLTGDLGSYNVDGTIEYHGRKDTQVKI

>Trichoderma longibrachiatum_mod7_Gln

AQQAARDPHHEAVYSSEGTVTYATMDRLSNLLAHHLHALGVRPESVVPFCFDKSAWAIIAMLAILKAGGVFLPL
DPSHPRNRREALVQEVKAEVMIVSPSSSVTCEGLTPTMVELTTPLLEQLSSTYDAFRQIHPKPKPSNAAYVLFTSGS
TGKPKGVLMEHAGFATSTLGHGRVYNLGPASRVFQFSNYVFDGSLGEIFTTLSFGGTVCVPSETERLQEAPTFMC
QARVNTAMLTPSFVRTFTTDQVPSLQLLVLGGEASSKDLIETWCDRLRLVNGYGPAEACNYATTHDFKPTDSPRT
IGRGFNSACWIVEPADYNKLTPIGCVGELIIQGNALARGYVNDPKRTAGSFVTAVDCLPTDRISGPHRFYLTGDLV
RYNSNGEMEYLGRKDTQVKL

>Trichoderma longibrachiatum_mod8_aib

EQQAIRRPQAPAIDGWDASFTYGQLNEAANRLANHLVAEYDIKNDELIHVCFDKSAWFFVAILAINKAGGAWIP
LDPSHPAQRHQRVVSQTKARLALVSTSHISTCVDLVDEVLEVSSTTDEILRKSASSRSGPARQVSSSNTAYVLFTSGS
TGTPKGLVMEHGSVCTSQTAIVKRLRMTPSVRILQFAAFVFDLSIGEIVAPLITGACICVPSEHDRMNALPEFVRQH
KVNWAYLTPSFIQTLSPKDVPSLELVLLAGEAVSRDILDAWFGKVRLVNGWGPAETCVFSTLHEWQSADDESPLT
IGRPVGGFTWIVDPENPQKLAPIGVPGEVVIQGPTILREYLDDPVRTAASTVYNLPAWAPNRGSKWNRFYKSGDL
CSYNADGTIEFVSRKDTQIKI

>Trichoderma longibrachiatum_mod9_Vxx

DEVASVRGDALAICGWDKSFTYKEMSETTNRLAHHLVNDYGVKVGDVIHVCFEKSAWFIIATLAINKAGAAWS
TLDTSHPIERYQKIVSQTGSKLALASAVNSHRCAGLLPHVIELTSELDARLAQNASWSACGPAVTITPSDAAYILFT
SGSTGVPKGVVIEHASLCTGQTSLSQALDFHEGFKVLQFSSYSFDAALFEIGSTFLTGACLYVPSWDEQMNELVDYI
RKHEITCTLLTPTLARTIRPEDVPSLDMLLVAGEAPTRDILDIWFGKLRLTNAWGPTECCVIACIHQWTSVDESPK
VIGRPIGGSCWIVDPDDATRMAPVGTVGEIVVQGRNVFRGYLSDPAKTAAATVTGLPDWVPKRDSAHWDRFYL
TGDLGFINEAGNIEYCTRKDTQVKI

>Trichoderma amaonicum-mod6

LEKAIAVNHEVPEFMDTCLHTLIEIQADQRPNAIAVSASDGEFTYRQLNNAANRLAHHLVDRYSVRVEDLIHVC
FEKSIWHIISVIAINKVGAAWVPLDPSHPEQRLQQIVEQTGAKLALASSSHTALCAKVIKNVLEVSSIIDDQLVNAR
VSSSNPAVPVSPRNTVYVLFTSGSTGTPKGIVLEHAAVCTGLNAISKRLGLPASARMLQFSAFTFDVSVGEIFTSLIS
GACVCIPSDWERMNNITAFIQRNHINWAFLVPSYIRTIDPDDVPSLGVVALMGEPATKDVYNAWFGKVRLFNSW
GPTETCVISSSHEWKSANESASVIGKPLGAYCWILDMRNPTQLAATGIVGELVVQGPTLLREYLAAPDKTKESIIYD
LPDWIPHQALPGYDRLYKTGDLCFYNPDGNIEYYSRQDTQIKIRGHRVEAGEIEHRVSQAMTVDQVAVELVGSTL
VAYLCFNLDCKPISLTATPDEIFIPLTEDMQQLISTAVAQLKTTLAHYMVPTTFIVCKFMPS

>Trichoderma amazonicum-mod1

DLRRAMECNSEEPDIISSCLHQIIEDHALKRPDAMAVHASDLTLTYSQLNLAADRLSSYLIDNYTVQARDLVHVC
FEKSAWFFVSILAINKAGAAWVPLDPSHPIQRHRKVVTQTEAKLALASPNNTSLCSDLVDNVVEVSASLDEMLIK
RRQGNRNSRTVSPSDACYVLFTSGSTGTPKGFVMQHRALCTSQTAATKRLRMTSDIRVLQFASYVFDMSIGETVG
PWIAGACICVPSEEVRMNGLAEYIRMMKINWVYSTPSFSRTLNPDDIPGVELLLLAGEAVSRDILETWVGKVRLIN
GWGPAETCVFSTLHEFESLDDSPLTIGRPVGGFCWLVDPENPHRQVPFGTIGEVVIQGPTLLREYLADPAKTESTT
VKPLPDWAPNRNLDHWNRFYKSGDLCKYNSDGTIAFVTRKDTQIKIRGLRVELGEVEHQIKACLDGVHQVMVD
KIDTEAGSSLVAFFCFSTETRVVTTADIVADGNTTMFLPITSQLRSQIVGMIGHLSVNLPKYMIPSL

>Trichoderma amazonicum-mod10

LELAIASNSVTPAVVDSCIHELIETRAQISPDAPAISAWDAELTYSQLIAASNRLAYYLIHTHGVKPNDLVHVCFEK
SAWFFVSIIAINKAGAAWVPLDPSQPEERLLQIAHQTEARVALVSPKNTTLCTNLVENVIEITAQLDRTLSLSVPKS
YAPRLAVSPYSTAYIIFTSGSTGTPKGVVMTHISLCTSQTALAERLGMTSSVRILQFSAFVFDMFIGETMTSLIYGACV
CVPSDSERMENLVGFINKKRVNWAFLTPTFARTIQPDDVSGLQLLLVGGESIPQDLVVKWFGKLRLICAWGPTES
CVISAVHEWTSAEESSLVIGRPVGGFCWIVDPDMPHRLAPVGTVGEIVVQGPTLLREYLNDPQRTEAALIPKPTW
ALQPDSQHWNRCYRSGDLCFYGHDGNIVFSGRRDTQVKIRGFRIELGDIEHHIRSQLDCPCEVAVDILKTTAGAS
LIAFISFNIEANASIGDSGADIFLQLEPDMQNKFSSLLGRLKALLPPYMVPTNFVPCQY

>Trichoderma amazonicum-mod11

FSHKVNSEALPEILDTCLHKLIELQAETRPDGMAIQGWDKNFTYHELNRAANRLAHHLVTEIGVKPYDIVHVCFS
KSAWYVVAILAINKAGAAWGPIDPSHPLQRHKQIVSQTKARVALASLDNVERCSKLVPSVVAVSSSLDSNLSEKG
CDSRHGPDVTVTALHAAYVLFTSGSTAVGKRFGFTPKVKILQFTTYVFDFSISEIIAAMLFGACVCIPSEETRMNRL
KDFVQESGINWLFLTPSFLQTLRPDDVPSVELVAVGGEPLPRALFEEWVDKVRFFNCWGPSETCVMSAMHEYKS
ADESNMTIGKPVGGFCWIVDPEDPQRLAPVGAVGELIVQGPTLLREYLNDPAKTQASILYSRPDWALCPDTEHW
NRFYKSGDLCSYNPDGTIKFHSRKDTQIKIRGLRVELGEIEYRIKEALDGALQVAVDVFKSDTASVLAAYFCFSDAT
AVMSGTDVDDVFMPLTEEVQHRIAAMVGEIKISLPEYMIPTLFIQCQ

>Trichoderma amazonicum-mod12

IQQSHKLNFDVPEIINSCFHHLVEDQATYRPNSMAIRSWDGDFTYAELDQAANRLANHLVQTYDIQTDELIHVCF
DKSVWYFISILAINKAGAAWVPIDPSHPEQRLRQVVNQTRAKIALCSPEYIDLCFSLVESVVQVGPGLDSQLLQLV
NSQQGPASRVSPNNIAYVLFTSGSTGTPKGLVMQHRSVCTSQTSVARRLGLTPEVRMLQFAAFVFDLSIGEIIAPLI
TGACLCVPSAHARMNELPKFIQDTNVNWAYLTPSFVRTLKPKDVPGLELLLLAGEAVPRDVLTAWFGNVRLING
WGPAETCVFSTLHEWKSIDESSLTIGRPVGGFCWVVDAEHPDRLAPFGTIGEVVIQGPTLLQEYLADPERTNASTM
SSPEWAPHPDEINWNRFYKSGDLCRYNADGTLEFSTRKDTQIKIRGLRVELSEVEYHIQQALTGVQQVAVDVFRG
DSGSNLVAYICFNDETRTAGIQVDKVETPFSSPDEILRQLVALAGELAITLPRYMIPTLFIPC

>Trichoderma amazonicum-mod13

LLSPWDLHHAVESNRLSPSSEACTHWLIQETIRSRPNDTAIASWDAELTYSQVGIFASRLAVKLQERGVGPETLVL
LCFPKSAWAVIAMVAIEMAGGAFVPLDPAAPAARLKGIIDDTHAILAIASPSTGTVLEGLGVEILSVDQEMLSTLP
DPTHQVVSNVQPQHASVILFTSGSTGKPKGMVIQHNNLCSSSDAYGNKLHIGPGTRVFQFSAYTFDVGILDCLVS
LMRGACLCIPSDHDRMNNLSGAMRSLKATWVFLTPTVADLLSPTDVPDLKVVCLGGEAISKKCADRWVNRVKL
HGLYGPAEASICAWNPTVGKSGRSTNLGRPISSAFWVVEPSNIRQLVPVGCIGELLIEGPMLARGYLNVSAEVAAS
WMDDVDWLPGEKKRVYRTGDLVRRNADGTFDYMGRKDTQIKLHGQRVELGEIETQIHQFLPDNMAAMVDILK
ADDENAHDILLAFLWYTEGDTAQSQSLSLMESLPEKTQRFICHLDSSLAESLPSYMIPSSYLIFEGKP

>Trichoderma amazonicum-mod14

DVEQAIERNSYGIEPIESCVHELISIQAAKNPQHEAIYSSSGSMTYREVDKLSNQLASHLIRLGVKSETIVPFCYEKSV
WAIIAMLGIIKAGGVFLPLDPAHPLSRRQALIDETSAQYMIVSPTTVEECKGMTKNNIELSPTFFSKTKSSARARKF
KPVKPRPGSAMYILFTSGSTGKPKGVIIEHKAISSFLVKVSDVLHMNGDSRMLQFSSYGFDASIFEIFATLISGGTVC
VPTEAERMQHTANFVEQARINSALLTPTFVKTLTPYAVPSLRMLLLGGEAPSKEIIDLWRQHVSLWNAYGPTETCI
MSTLQHYTDATTPATSIGRGFAHHCWVVNPENHHELTPIGCVGELLVQGDSLSRGYINDEEKTNNAFINHVKW
LPANVDVGKRRFYKTGDLVRYNPDGSFNYLGRKDTQVKIRGQRIELGEIEYQIKFHLPDLKHAAVDIIHHDTHES
LVAFVNFSGDNDPSEVAQVQLVSHNDELKELFAQLVANLATVLPSYMVPKYFIAVESMP

>Trichoderma amazonicum-mod2

QQSMDWNAHKVVIEEHCMHDIISKRAKSCPNDEALYSSEGSLNYAALERLSDLVAYQLLEYNVQPETVVPFCME
KSIWAVVTMIGILKAGGAFMPLDPSHPESRRLALVQEVNAKVIITSPSTALSCEGMAQHTIQVSSSLLTHAPNTAK
SYRELSNYKKPEPHNAAYVLYTSGSTGKPKGLVMPHSAACTSLLRHPEKFSINKSTRTFQFATYVFDVCILEIFVSLF
VGATVCVPTETERVGNTSRFMTEARVTWTCLTPSFIGTLDPDTIPTLQTLCIGGEAPTRDILSKWHGRAELINAYGP
AEACVDVAGHIFMSPDDSPTNVGRPFAHKLWIVEPDNHNRLAPIGCIGELVIEGHAIARGYINNEEKTKASFVDD
LEWLPSATSSSRPRVYKSGDLARFNPDGTIEFFGRQDTQVKIRGQRLELGEIEYNIKAKLADVQHTVVDLIEREAGK
MIIAFITFKNHSCDTTADAADPYGNFIKDDKLISSFQDLMLELRRVLPGYMIPSIIFPLR

>Trichoderma amazonicum-mod3

IEFAHAANPNAPNVVDSCIHTLIEERTRINPDSSAVCSWDANFTYSELDENANRLANYLMNVLKVKAGDLVMV
CFDKSAWFVVAILAINKIGAAWVPIDPSHPTERHQSIVKQTGSTLALCTPSNSLKCGQLAAKVLKVNTEFISELRR
VEAYAGKPDVKVNPNDVSYIIFTSGSTGVPKGVVIQHRAICSSQVALSQRLGMHDGVRMLQFSSFVFDASLFEILSP
LISGACVCIPSWDMQMNSLPAFIRDVNVTWAFMTPSVAGILRPQDVPSLELITVGGEAPSKEVFNIWLGKVRLFN
VWGPTETTVIATVHEVKSAPTQGSHLTIGQPICGNCWIVDPEDPTRLAPVGTVDPEKTAAAVVTSLPQWTPNRDL
WNRFYRSGDLAVYNSDGTIKYCSRKDGQIKIRGLRIESGEIEHHIRKNMDTPGQVAVEALRSEAGTNLVAFICQNS
DTIPASMTNNMTSEDIVLPLTDELKSSLGALRSSLISSLPGYMVPTFFVPCK

>Trichoderma amazonicum-mod4

DKAVLFNPEMPEVIENCFHQLFERQALETPDAMAIRAWDSSFTYAELDSAANRLAHHLMAQHTVKLDELIHVC
FDKSAWFFVSILAINKAGAAWVPLDPSHPLERLRQIVTQTKAQVILTSESHAALCSGLVSLVIEASDSLDRQLSSNN
KYSQSSPVTKVSPNNAVYVLFTSGSTGIPKGLVMEHRSVCSSQKAIIKRLGLHSNVRMLQFAAFVFDLSIGEIVAPLI
SGACVCVPSEQARMNDIAGFIRETGVNWAYLTPSFIRILKPEEVPALELLLLCGEVTPRDVFNTWVGKVRFISGWG
PAETCVFSTLHEWKSDAESPLTIGKPVGAFCWIVDPERPDQLAPIGTVGEIMLQGPTLLREYLDDPERTRSSVTTSL
PNWALQRAHQWTRFYKSGDLAMYNSDGSIEFCSRRDTQVKIRGLRVELTEVEHQIRESLEGIRQVVVDVATSDSG
SNLVSYLCFSEETRSSNSKAHLDDIFMPLTAELQPSLAAMIGQLKVILPNYMIPTLFIICR

>Trichoderma amazonicum-mod5

DKAVLFNPEMPEVIENCFHQLFERQALETPDAMAIRAWDSSFTYAELDSAANRLAHHLMAQHTVKLDELIHVC
FDKSAWFFVSILAINKAGAAWVPLDPSHPLERLRQIVTQTKAQVILTSESHAALCSGLVSLVIEASDSLDRQLSSNN
KYSQSSPVTKVSPNNAVYVLFTSGSTGIPKGLVMEHRSVCSSQKAIIKRLGLHSNVRMLQFAAFVFDLSIGEIVAPLI
SGACVCVPSEQARMNDIAGFIRETGVNWAYLTPSFIRILKPEEVPALELLLLCGEVTPRDVFNTWVGKVRFISGWG
PAETCVFSTLHEWKSDAESPLTIGKPVGAFCWIVDPERPDQLAPIGTVGEIMLQGPTLLREYLDDPERTRSSVTTSL
PNWALQRAHQWTRFYKSGDLAMYNSDGSIEFCSRRDTQVKIRGLRVELTEVEHQIRESLEGIRQVVVDVATSDSG
SNLVSYLCFSEETRSSNSKAHLDDIFMPLTAELQPSLAAMIGQLKVILPNYMIPTLFIICR

>Trichoderma amazonicum-mod7

EHDFAVSANGIPPVAIKQCIHSLIEEEANKHPESYAIQAWDADFTYQQLDQCANMLANYLISSLHVKIGDIVHVC
FDKSAWYIVAILAINKAGATWSPLDPTHPVQRYQQIISQTGSSLILTSPTNTSKCATLADSILEISSGFHEILVKDVTE
RRPDVAVAPEDAAYILFTSGTTGVPKGVVIEHQSLCTSQTALAQRVGFTNDTRMLQFSSFVFDASVLEIIAPLVMG
GCVCIPSWDEQMNDLAGFIVNASVNAVFATPTVARSLRPEEIPGVEILVVGGEAAASDILETWIGKLRLFNAWGP
TEICVAGSVHEWKGPNESPMTIGRPILGNWWIVDPNNPKQLVPIGCVGEIVCQGPTLFREYLANPAKTQEVIVTSL

PSWAPDRDSVDWGRFYRTGDLGHYNPDGTVEYAGRKDTQVKIRGLRVELHEIEHQLFAHLPRVRQVAVNVMK
REETSHLVAYYCFSNDTLPSHLAADNAGKGMFLPLRDDAMPQIQDLIAHLKGVLPPYMIPSTFI

>Trichoderma amazonicum-mod8

VEQSVKFNPEAPEIMDTCVHTLIEAQAHREPDALAISAWDGNLTYRQLNESANRLAHYLVKNYNIRPDDLIHVC
FEKSVWHFVAIIAINKAGAAWVPLDPSYPEQRLRQVISQTHSTLVLASSRNAKLCSSLVANVLQIDVELDKALILA
EDGHKGPTIAVTHRHAAYMLFTSGSTGKPKGLVMEHGAVCTSQTAIAKRLNLTSDVRMLQFAAFVFDLSIGEIIG
PLISGACLYIPSEETRMNGLKQYVNETGVNWAYLTPAFVRTFVPEDMPGLELLLLAGEAVPREVLSTWFGKVRLV
NGWGPAETCCFSTLHEWSSVDESPLVVGRPVGGFCWIVNPEDPQQLTPTGTLGEIVIQGPTILREYLADTERTKDS
TVYSLPKWAPQPDSEHWNRFYKSGDLGYYNPDGTIVFSSRKDTQIKIRGLRVELGEVEHYIRETLEGVCQVVVDV
YQKDGRSNLVAYICFNDEMKNPSADEEFTAAALFALITDDMQQRIAIMLGELRITLPQYMIPTMFI

>Trichoderma amazonicum-mod9

SWDLQQAINASRLRPATESCTHWLIQEKIKEQPEEIAITSWDGNLTYAQLGANATCLAAKLQDLGIGPESLVPICF
HKSKWAVVSMVAVQMAGGAFIPLDPGAPPARLQGILQDTGAALAISDPSSQATLQSLGIDVITVDDEMMSNIPSS
VALQCEVQPANASVVLFTSGSTGKPKGMVIQHNSLCSSSDAYGSDLNIGPGTRVFQFSAYTFDVGVLDCLVSLMR
GACICIPSDHDRLNNLAAAITTSQANWVFLTPTVADLLHPVDVPTLKTVCLGGEAISKKCADRWVNLVDLHGLY
GPAEASICAWNSVVGKSGRSTNLGHPISSAFWVVDVNDPKSLVPLGCIGELLIEGPMLARGYLNVSADVAANW
MEGVDWLPGSDQPRRIYRTGDLVRRNADGTFDYIGRKDTQVKLHGQRVELGEIEAWINEYLPDNMAAIVDVVK
DKDGPDSLLAFLWYTEEASNAPTRLMESVSDEARAAILHLDTSLSSILPPYMIPSSYLLFSGKPEQT

*>Trichoderma asperelloides_mod1****

DIQRAIEYNSEEPDIINSCIHQIIEEHAREHPDAMAICAWDMKLTYSQLNTAADRLAGYLVHDFAVKPNDLIHVC
FEKTVWFFVSLLAINKAGAAWAPLDPSHPIKRQQQIVSQTKAKLLLTSPNNVRICYGLVEHVLEVSSALDEMLKS
RKQDRQNLNLVSPDAVAYVLFTSGSTGTPKSLVMQHRAACTSQTAIVKRLGMTSDAKMLQFASFVFDLSIGEILG
PWIARACIYVPSDETRMNGLADYIRTMNINWMYSTPSFSRTLNPDTIPGLEVLLLAGEVVGRDILETWFGKVRLIN
GWGPAETCVFSTLHEWASLGESPSTIGKSVGGFCWIVDDSNRLAPIGAVGEIIIQGPTILKEYLSDSAKTEAAIMRSL
PDWVPKRNFDCWNRFYKTGDLGKYNQDGTIEFVSRKDTQIKIRGLRVELGEIERMLKRLVELLDPKSLRTYSLVES
IKNAPETAMESRLQAIWAKRLNIPIESIGRDDGFLELGGDSIAVIHFVAEARQAGMSLTV

>Trichoderma asperellum_mod_12_Aib

KQSVVRPDAPAVCAWDGEMTYNELNNAANKLAHRIVNAGVKTNDLVHVCFEKSLWFFVSILAINKAGGAWV
PLDPSHPKQRLRQIAGQTLAKLALSSPTNAALCRDIISEVIEVNGELINELSETYDGVNGPITRVPSSNAAYVLFTSG
STGTPKGLVMQHQAVCTSQTAIAQRLRLTPDVRILQFAAYVFDLSIGEIVGPLIHGACICVPSEEMRFNGLKEFIHE
KNINWAFLTPSFVRTLKPEDVPSIELLLLAGEAVGRDILNMWFGKVRLINGWGPAETCVFSTLHEWTSIDESPLTV
GKPVGGYCWIVDAEDPSKLAPIGCLGEVVLQGPTLLREYLADPQRSKEAIITPLPSWAPKQDSQPWSRFYKSGDLC
FYNPNGTIEFSSRKDTQVKI

>Trichoderma asperellum_mod1_Aib

ERQARERPNAPAIQAWDLELTYEQLNRAAHRLAHYLINTWGVTDQDLVHVCFEKSAWFFVSIVAINKAGAAW
VPLDPSHPIQRQQQVVSQTKATLALASPTNAGMCSELVDNVVEVSSTLDEELAKTEMSSYEMMRAVSPYNAARL
YFTDCNYQAPWDDVRHFINTMKINWAYLTPSFTRTLNPSDVPGLELLLFAGEAVGRDVFEAWFGKMRLINGWG
PAETCVFSTLHEWKSLEESSLTVGSPVGGYCWIVDPQNPQRLAPIGTLGEVVIQGPTILREYLADADKTEASMVRS
LPEWAPKRTARHWNRFYKSGDLCRYNADGTIEFGSRKDSQVKI

>Trichoderma asperellum_mod10_Gly/Ala/Ser

ERQTEERPNALAVDAWDGKLTYGELDRAANRLARLLISRYTVKVGDIVHVCFEKSLWYIVAILAINKAGASWSPI
DSAHPFHRLQAVAIQTGAKLALASTANRALCERLVDSVVEVSASLEEALSAEGISDMGPDVCVTSNDTAYILFTS
GSTGMPKGIVMQHGALCTSQTALGERLGLDHTVRMLQFSSFVFDVSVGEIMGSLINGACVCIPSDEMRLNSLDVF
VRDFKATWAYLTPSFTRTLNPKDFSGLKLLALMGEATTQDVLDTWYGLPNMRFVNGWGPAETCVLSSTYEWQS
NTESTLTIGKAVGSYVWIVDPENPMRLAPTGCLGEIIVQGPALFREYLADPNKSAAAIVTNLPEWAPRRESAAWN
RFYRTGDLGFYDHNGVLNFVSRKDTQVKV

>Trichoderma asperellum_mod11_Leu/Ala/Aib

ENQAKLNPSAPAIAAWDGNFTYSELDSAANKLAHHLINAYGVKFNDFVHVCFEKSAWYIVSIIAISKVGATWVP
LDPFQPEQQLRSIVSQTKATLALVSPRNVELCSSLVDNVIEVTTDLDISLPGQDGVQSPNVSVSSQTAAYVLFTSGST
GTPEGAVIEQGSLCTSQNDIAKRLHYHSNVRILQFSAFISHLSISEIIMSLINGACVCVPSEDQRTNGIVDFIRNMRIN
WLYITPTFLRTLNQSDVPGVELVLMGGESVPRDVFDMWFGHVRLLNAWGPTESCMMGAIHEFEYADESPLTIGS
PVGGFCWIVDPENPHHLAPIGSLGEVVIQGPALFREYLNNPEKTEETTVNVLPDWAPRPDERNWNRFYKTGDLC
FYNANGAIEFSSPKDMQTKI

>Trichoderma asperellum_mod13_Pro

RIKAQPNDPAISSWDGNLTYAELGTLANRLAWKLQDLGVGPESLIPLCFPKSTWAVVAMVAVEMAGGAFVPLD
PNAPAARLQSLIEDTKSTLAIASPSCQATIRDIGIDVFVVDEALLLELSDPIAPITSSVQPDNASVILFTSGSTGKPKG
MVIQHNSLCSSGNAYGTDLNIGPGTRVFQFSAYTFDVGVLDCLVSLMRGATICIPSDHARLNDLAGAMNATKAN
WVFLTPTVADLLSPTDVPYLEVLCLGGEAISKKCAERWINHTEVHGLYGPAEASICAWNPAVGRSGLSTNIGRPT
SSAFWVVEPSNNKRLVPVGCIGELLIEGPMLARGYINANAESASNWMENVDWLPGPDIKRVYKTGDLVRRNAD
GTFDFIGRKDTQVKL

>Trichoderma asperellum_mod14_Leu

EQQALLNPDAPAINAWDADFTYAELDRAANRLANYLIQSLGVKVGDFIHVCFEKSAWHVVSVLAINKAGAAWI
PLDPSHPTERHQQVVNQTSSVLALTSVANVSKCIDLVANVLEVTRELDDRLIADVQFNDTKPATSVTPQDIAYILF
TSGSTGIPKGVIMEHVALCSSQTSASKRIGYGSSVRMLQFASFVFDACIAEIFTPLISGACVCIPSWETQMSSLASYIR
KANVNWALLTPSFARTINPAEVPCLELLMLGGEAVSRDVFELWFGKLRLFNAWGPTESCVFGALHEWKSIHESQ
LTVGQPLGGYCWLVDPKNPQKLAPTGTLGEVVIQGPNLFREYLSDKEKTASSTVASLPQWAPNQHSRYYNRFYK
TGDLAMYNSDGTLQYYSRKDTQVKI

>Trichoderma asperellum_mod15_Aib

EAQAISRPNAIAINAWDAKFTYAEFNRAANRLANHLVATCNLGVGDLVHVCFEKSAWFFVSILAINKSGAAWA
PLDPSHPEQRLRQVVSQTCAKIALASTENSTLCASLVESIITVNSDLDLQLSKVEEYSQKGPVTTVSPDSPVYVLFTS
GSTGTPKGLVMQHRSVCTSQTAIAKKLSLHSDVRMLQFAAFVFDLSIGEIIAPLITGACLCIPSENTRMNGLVQFIR
EMDVNWAFFTPSFIRTLNPSEVPSLELILLAGEAVPRDVLTTWFGKVRLINGWGPAETCVFSTLHEWQSINESPLTI
GKPVGGFCWIVEPDHPDRLAPVGTLGEVVIQGPTLLLEYLADPERTQASVLDNLPQWALHSRPDSKHWNRFYKS
GDLCYYNQNGTIEFSTRKDTQIKI

>Trichoderma asperellum_mod16_Aib/Ala/Vxx

EAHAKEAPTSPAICAWDGVMTYGQLNEMANILAHHLIAHYSVQVEDLIHVCFEKSVWYWVSVLAISKAGAVW
VPLDPSHPEQRIRQVTSQTHSKLALVSDTTRHLVSDIVDQVLEVSPALYERLNSPLGTEDPQVPVSSHNAVYVLFTS
GSTGTPKGLVQTHGGVSSTLSAIRNRMGIDSNNRTLQFASYVFDMSIGEGILHLISGACIFIPSEDTRMNGLTEFINE
HKINSLWLTPSFVRTISPEQVPSVDFLLLAGEAVTRDVLTTWCGSVRLFNGWGPAETCVFSSLHEFTSIEESPLTIGR
PIGGHCWIVDPENPYKLAPIGTLGEVVIQSPAILREYLDDVERTKASTIYDLPAWAPRRDEPHWSRFFKSGDLALY
NPDGTLEFSSRKDTQVKI

>Trichoderma asperellum_mod17_Gln/Glu

AEHALRDPTHEAIYSSEGSLTYGELDHLSDILAAYLSSLSVGPETVVPYCFEKSMWAVIAILATLKTGAAFMPLDPS
HPVKRRAELVKEVKARVLIASENAATSCMAEHIVELSPSVLAKLSASVIPRAFPKVGPRNAAYVLFTSGSTGKPKG
VVMEHSALTTSTIGYGRVYSFSPASRVFQFSNYIFDGSLGEILATLAFGGTVCIPNESERLQDAPGFIRKARINTAML
TPSFVRTFTPDQVPNLKTLVLGGCVGELVIQGHVLARGYINDSERTKTSFVTEISSLSTQTITGPQRFYLTGDLVRYC
ADGSLEYLGRKDSQVKL

>Trichoderma asperellum_mod18_Valol

SKQAARRPNHEALYSTEQSMTYTELDNLTTQLATYLYGLGIRAGAIVPFCFEKSMWAIVAMMGILKVGGTFIPLD
PSHPVSRRQALIDEVGAQFMIVSPTTAPNCQGMVQSTIELSPSLISFISTRNATTQTVISSDPNNAAYILFTSGSTGKP
KGVVIDHKAVSAVLLRQREAYAYNDDTRMLQFANYTFDACIAEIFTSLAVGATVCVPTEHERLHNTAAFIREAR
VNHGLLTPTFIKTLSPEQIPGMKSLLTMGEAPSRELIETWAGKIDLHNCYGPAEACVTSTNYTYSPSIKTSATHLGR
SFTHGIWIVNADDHNRLVPVGCVGELLLQGSCLARGYINNEEKTRQSFIEDVDWLPSGVNIGERRFYKTGDLVRY
TPDGSIEYLGRKDTQVKI

>Trichoderma asperellum_mod2_Gly/Ala

KQIDANPDAPAIDSWDAKLTYGEVGTLASRLAVKLQSLGVGRDTLVPISFPKSTPAMITMVAIQMAGGALVPLDP
AAPLARLQSIVSDTQAKLIVTHPSLEDKLQELGVDLLIVDNSMLHQLPDPESKFITSGATPDSLYAVLFTSGSTGKP
KGIRIPHSSLCSASDAHAASTGVGPGARVFQFSAYTFDVGVLDVLITLMRGGCVCVPSEYDRLHNIAGAINALQA
NWALLTPSVADMINPAEVPCLTTLGLIGEAIYKKNSERWKGYADLHGLYGPAEASVCAWNSDIHNGPSTNLGFP
LSSAFWVVEADDPSRLVPIGCIGELLIQSPILACGYLNADAKNTANWLEDMTYDWLPAAGPKRAYRTGDMVRR
NPDKTFEYMGRKDNQVKI

>Trichoderma asperellum_mod3_Aib

KKQAIERPDALAIAGWDRTFTYAELNEAANRLAHYLTQNFAIKADELIHVCFEKSAWYFVAILAINKAGAGWVP
LDPSHPEQRLRQIVYQTRAKIVLTSPASVNICSTLDMSIIEISPVFDEKLIKSGMPSATGPAVIVTPRNIAYVLFTSGST
GTPKGLVMEHGSVLTSYIQEHKINWAYLTPAFARTLKPEDVPALELLLLAGEAVSRDVFESWFGKVRLINGWGPA
ETCVFSSLHEWKSA-S-ESPLTIGRPLI

>Trichoderma asperellum_mod4_Leu/Gln/ala

EHQAEQTPHATAVHSWDGEYTYSELDLAANRLANLLVDCHGIKTGDFIHVCFEKSIWYIVSILAINKAGAAWVP
MDPAHPFQRLQQVAGQTGARLALASSTHSHLCSELFDSVVEVSSAFNEQLKEDTAISNQKPSVNVKSRDAAYVL
FTSGSTGMPKGIIMEHAALCTNIVLSLMHGGCVCIPSDHDRLNNLSGFITEAKVNWAYLTPSVARTLRPEDVPSLE
FLLVGGEAASQDVLDLWFESPSNIGRSVGSFVWILDTEDYRRLAPGPTLLREYLADPVKTASSTLTSLPNWAPRAS
STNWNRFYKTGDLGFYDSDGNIHILGRKDSQVKI

>Trichoderma asperellum_mod6_Aib

SQQAARDPHHEAVYSSEGTLSYATLDRLSSLFAHHLQELGVKLESVVPFCFEKSVLAIIAMIGILKAGGAFMPLDPS
HPHSRHKALIEEVEAEVMIVSSSYFLACDGLTSTTVELTALLLEQLAFTYDAFQPIHPKSLPNNAAYVLFTSGSTGK
PKGVLMEHSAFSTSTLGHGRVYKLGPTSRVFQFSNYIFDGSLGEIFTTLSFGGTVCVPSETDRLQDAPSFMHRAQV
NTAMLTPSFVRTFTPEQVPSLQLLVLGGEPSSKDLLETWCNHVRLVNGYGPAEACNYATTHDFKDSDSPRTIGHG
FNSACWIVEPNDCNKLTPIGCVGELIIQGNALARGYINDAEKTSCSFITDIDCLPKSLIAGPHRFYLTGDLVRYNSN
GEIEYIGRKDTQVKL

>Trichoderma asperellum_mod7_Gln

EQQATVRPDAPAVHGWDTKFTYAQLNEAANRLANHLVQEYEIEFNELIHVCFEKSAWFFVAVLAINKAGGAW
VPLDPSHPAQRHQQIVTQTKARLALVSSSNVSTCIDLVDHVIEVSSTTDENLSRVGSSQRNPDLETSPRHAAYVLFT
SGSTGTPKGLVMEHGSVCTSQTAIVRRLGMTTSVKILQFAAFVFDLSIGEIVAPLITGACLCVPSEHDRLNELPKFIR
QNNINWVYLTPSFIRTLNPKDVPSVELVLLAGEAVSRDILDVWFGKVRLINGWGPAETCVFSTLHEWKSVDESPL

TIGHPVGGFCWIVDPENPNKLAPIGTLGEVVIQGPTLLREYLSDTARTAASTVYSLPDWAPNRTVQHWDRFYKSG
DLCSYNADGTIEFSSRKDTQIKI

>*Trichoderma asperellum_mod8_Aib*

RGNALAISSWDACFTYEEISATTNRIAHYIVNTYGIEVGDTIHVCFEKSAWFMIAILAINKAGAIWSALDPSGPAEY
HRKIISQTGAKLIVASATNSHTCADLLPNVLKLTPELDAKLTENFGQNISRPTVNITPGDAAYVLFAAESSDIPEGV
VMEHASLCTSQMSLSHALDLDESFKVLQLSSYSSDAALFEICSTFLVGACLYVPSPDEQKNQLEDYIRKHKINCAL
LTPTLARTIRPDGVPSLDVLLIGGEATTRDILDIWCNKLRLFNIWGPAECCVAASIHRWTATESPNVIGRPISGSCWI
VDPDDPKSMAPIGTVGEIAVQGRNLFCEYLSDLVKTVAATIIGLPSWVPKSDSAYCNRFYLTGDLGFINEDGNME
YCTRKDSQIKV

>*Trichoderma asperellum_mod9_Aib/Ala*

DHQATIQPHALAIISWDRNFTYKQLNEASNRLAHFLVNKHSVKPDDLVHVCFEKSAWHCVAILAINKAGAAW
VPLDASHPEQRLRQVISQTRAVLALSSPSNSALCATLIANVVQVDTELDNRLSEIEPSANGPDVAVSSGNAAYVLF
TSGSTGTPKGLVMEHGSVCTSQTAITKRLGLHPKVRMLQFAAYVFDLSIGEIIGTLISGATICVPSDHIRLNNIVDFIR
KERVTWAYLTPSFVRTIKPSEVPNLELLVLAGEAIPRDVFTSWFGKSLDESSLTIGRPVGGFCWIVDPENPHCLAPT
GTLGEIVIQGPTILREYLSDTERTEAALVKSLPEWAPFREEANWSRFYKSGDLGFYNPDGTIQFSSRKDTQIKI

>*Trichoderma citrinoviride_mod1_Aib*

ERQARTNPNSPAIQAWDMELSYSQLDRAANRLAHHLVKSCGVKDQDFVHVCFEKSAWFFVSVIAVNKAGATW
VPLDPSHPLQRQQQVVSQTKATLALASPSNVEMCTELVDNVVEVSSTLDEKLAKTEESSYGPIRNVSPDNAAYVL
FTSGSTGTPKGLVMQHRAVCTSQTAITKRLGMTSNVRMLQFASFVFDLSIGEIVGPWVVGGCLCVPSEETRMNNL
VDFINTMQINWAYLTPSFTRTLNPDDVPSLELLLFAGEAVGRDVFEAWFGKVRLINGWGPAETCVFSTLHEWKSL
EESPLTVGTPVGGYCWIVDPHDPQRLAPVGTLGEVVIQGPTVLREYLADPTKTEASLVRN-
LPEWVPNRTAAHWDRFYKSGDLCRYNADGTIEFGSRKDSQVKI

>*Trichoderma _citrinoviride_mod10_Aib*

EKQAAAQPDAPAVLSWDRDFTYRELNEASNRLAHLLVNKYDVKPNDMVHVCFDKCAWHFVAITAINKVGAA
WVPLDASHPEMRLRQIVSKTKATLALVSSSNASLCASLVERVLEVNTALDNELLAAETGEHGPLVDVSSHSAAYV
LFTSGSTGVPKGFVMEHGSVCTSQVAIAKRLGLGPNVRILQFAAFVFDLSIGEIIGPLISGGCICVPSEHTRMNGIINF
INEMKVTWAYITPSFARTIRPSEVPSLELLLLAGEAVPRDVFAAWFGSTVRLINGWGPAETCVFSTLHEWKSADES
PLTVGRPVGGFCWIADPEDPSRLAATGTLGEILIQGPTILREYLSDVERTEAAVIKSLPEWAPFPNEPGWDRMYKS
GDLGFYNPDGTIEFSSRKDTQVKI

>*Trichoderma citrinoviride_mod11_Gly*

ERQVKHRPNAPAVDAWDGSLTYSQLDHAANRLAHLLVDDYAVQVGDIVHVCFEKSMWYIVAILAINKAGAA
WAPLDSAHPFQRLQAVAKQTGATLALASTANAGLCGQLVNRVIEVSAVLDETLSTNDVNSEEGPDVHVTPLDA
AYILFTSGSTGTPKGIVMQHGALCTSQTDISRWLGLDHTVRMLQFSSFVFDVSVGEIMEPLMNGACVCVPSDHMR
LNSLDAFVRDFNVTWAYLTPSFTRTLKPEDFPGLELLLLIGEAVTQDVLDTWFGLPNTRFVNAWGPAETCVFSTL
YDWQSNTESPLTIGRAVGAYVWVVDAENPRRLAPTGCLGEIVVQGPPLLREYLADLTKTAAATVTELPEWAPRR
ELTKWNRFYRTGDLGFYSHDGLLHYASRKDTQVKI

>*Trichoderma citrinoviride_mod12_Lxx*

ERQVKHRPNAPAVDAWDGSLTYSQLDHAANRLAHLLVDDYAVQVGDIVHVCFEKSMWYIVAILAINKAGAA
WAPLDSAHPFQRLQAVAKQTGATLALASTANAGLCGQLVNRVIEVSAVLDETLSTNDVNSEEGPDVHVTPLDA
AYILFTSGSTGTPKGIVMQHGALCTSQTDISRWLGLDHTVRMLQFSSFVFDVSVGEIMEPLMNGACVCVPSDHMR
LNSLDAFVRDFNVTWAYLTPSFTRTLKPEDFPGLELLLLIGEAVTQDVLDTWFGLPNTRFVNAWGPAETCVFSTL
YDWQSNTESPLTIGRAVGAYVWVVDAENPRRLAPTGCLGEIVVQGPPLLREYLADLTKTAAATVTELPEWAPRR
ELTKWNRFYRTGDLGFYSHDGLLHYASRKDTQVKI

>*Trichoderma citrinoviride_mod13_Aib*

EEQARIRPDAPAICAWDGEMSYSQLNVAANKLAHHILNAGVKSEDLVHVCFEKSLWFFVSIVAINKVGATWVPL
DSSHPEQRLRQVVSQTRAEFALSSPTNAALCGSLVKNVIEVSQSLLDNIPYDGVNGPALNVPSSNAAYVLFTSGST
GTPKGLVMQHQAVCTSQTAIAKRLRLTPNVRILQFAAYVFDLSIGEIVAPLIHGACVCVPSEEMRMNGLREFIQDT
SINWAFLTPSFVRTLRPEDVPGLDLLLLAGEAVGRDILDTWFGKVRLVNGWGPAETCVFSTLHEWASIDESPLTV
GTPVGGHCWIVDAEDSSRLAPIGCLGEVVLQGPTLLREYLADPQRSKEAIITSLPSWAPKQDSRPWSRFYKSGDLC
HYNPDGTIEFYSRKDTQVKI

>*Trichoderma citrinoviride_mod15_Vxx*

EQRALMSPDAEAVSAWDARFTYAELDRSANILANYLIQSMGVQVGDLVHVVFEKSAWYVVSILAINKAGAAWI
PLDPSHPAERHQQVVGQTQSRLALTSSANAAKCADLVSNVLEVTRELLDNLETQGNDTRPVTSVTPQDVAYILFT
SGSTGVPKGVVMEHGALCSSQTSISKRLGYAPGVRMLQFASFVFDACIGEIIAPLISGACVCIPSWETQMNSLTSYIR
EKNVTWAMLTPSFARTMDPSEVPCLELLILIGEAVSRDVFELWFGKLRLLNGWGPTETCVFGALHEWQSIEESQM
TIGQPVGGYCWIVDPEDPQKLAPTGTFGEVVIQGPNLLREYLADEAKTASSTVTVLPEWAPNRHSRHWNRFYKT
GDLAMYNPDGTIQYYSRKDTQVKI

>*Trichoderma citrinoviride_mod14_Pro*

QERIEKQPNDPAIASWDGDLTYEELGALASRLAWKLQSLGVGPESLVPLCFPKSTWAVVAMVAIEMAGGAFVPL
DPKAPVARLRGIIEDTKSTFAVASPSCQATLSEIGIEVLAVDEALLLELSDPVEDIQSKARPKNASVVLFTSGSTGKP
KGMVIQHNSLCSSGNAYGEDLNIGPGTRVFQFSAYTFDVGVLDCLVSLMRGATICIPSDHARMNDLAGAMTTH
KANWVFLTPTVADLLSPADVPYLKVLCLGGEAISKKCADRWVNYTELHGLYGPAEASICAWNPLVGRSGRSTNL
GRPISSAFWVVEPNNHKRLVPVGCIGELLIEGPMLARGYLNVSADVASNWMENVDWLPGSDKKRVYRTGDLVR
RNADGTFEFMGRKDTQVKL

>*Trichoderma citrinoviride_mod16_Aib*

ERQAEIRPNAMAIKAWDAELTYRDFNRAANRLANHLMASYDIKPDELVHVCFEKSAWFFVSILAINKAGAAWV
PLDPSHPEQRLRQVVSQTRARLALTSSTNRDLVASLVESVVTVDSELDAELSKVEEYSQTGPVTSVSSDNAVYVLFT
SGSTGTPKGLVMQHGSVATSQTAIVKRLGLTPDVRMLQFAAFVFDLSIGEIIAPLITGACLCIPSDHTRMNGLTQYI
RDTGINWAFLTPSFIRTINPAEVPGLDLVLLAGEAVPRDVLTTWFGKVRLINGWGPAETCVFSTLHEWQSVNESP
LTVGKPVGGFCWVVDPEDPHRLAPTGTLGEVVIQGPTLLREYLSDPQRTQASTVYDLPKWAPRPDSRHWNKFYK
SGDLCYYNQDGTIEFSTRKDTQIKI

>*Trichoderma citrinoviride_mod17_Aib*

EAQARETPDLPAVWAWDGQLTYRQLNEAANRLAHYLISDHNVQVEDLVHVCFEKSVWHWVSVLAINKAGAV
WVPLDPSHPEQRLRQVASQTRSTLALASDTTRHLVSDIIERVVEVSPTLFEQIDVRLGEKEPQVSVSASNAAYVLFT
SGSTGTPKGLVMTHGAFTTSQTAIKKRLGPNTNTRALQFASYVFDMSVGEGFVQLISGACIFIPSEHTRMNGLKEFI
AEHKINSLWLTPSFIRTLSPEQVPTVDFVFLAGEAIPRDVFTTWCTKVRLWNGWGPAETCVVSSLHQFTSLDESPL
TIGRPIGGFCWIVDPTDHSRLAPIGTVGEVVIQSPTILREYLADVERTKASTVYELPAWAPNRDQAPWSRFFKSGDL
ASYNPDGTLEFASRKDTQVKI

>*Trichoderma citrinoviride_mod18_Gln*

SQHASNSPHHEAIYSTEGSLTYGELDHLTDILATYLSSLGAGPETVVPFCFEKSMWAVVAILSILKAGAAFVPLDPS
HPTSRREALVKEVSARVLVASSNAITSCEGMAEHVVELSPSVMARLAASVTPKILPKVGPRNTAYVLFTSGSTGKP
KGVVMQHGSFSSTTIGYGKVYSLSPLSRVFQFSNYIFDGSLGEIFGPLAFGGTICIPSEDERLGSAPGFMNMAKVNT
AMLTPSFVRTFTPDQVPHLKTLVLGGEAASKSTLEMWVNRVTLYNGYGPAEACNYATTHVFKSSSE-SPRI-
IGSGFNGACWVVEPDNHSKLAPMGCTGELVLQGHALARGYLNDKAKTEQSFVGEIGSLPLSLLHEPKRFYLTGD
LVRYNSAGELEYLGRKDSQVKL

>*Trichoderma citrinoviride_mod19_Gln*

DNAQRDPDHEAIFSSEGSMTYAALDRLTDVLASHLCQLGVGPETIVPFCFEKSMWAVIAIVGILKAGGVFVPLDPS
HPVNRREALVKEVGAQIIIASESAAASCAGMAPRIVELSSDFMARLSAPNQTLVDPKRPSPSNAAYVLFTSGSTGK
PKGVVMEHSALTTSTIGYGRVYELSPASRVFQFSNYIFDGSLGEILATLTFGGTVCIPDEVERLQDAPGFVRKARINT
AMLTPSFVRTFTPDQVPGLKTLVLGGEPAGRDILDAWCDRVKLINGYGPAEACNYATWHPFASSQDSPRVIGKA
FNSSCWVVEPNNHHVLTPVGCVGELAIQGHVLARGYINDSERTRSSFVTEIASLSSAIAGPQRFYLTGDLVRYCSD
GSLEYLGRKDTQVKL

>*Trichoderma citrinoviride_mod2_Ala*

KQIEAQPDAPAFDSWDAKLTYREVGILATRLAAKLQSLGVGPDVMVPISFPKSTPAMITMVAIQMAGGALVPLD
PAAPLARLQSIVSDTQAKLIVTHPSLKDKLQELGIDLLIVDMSVLDKLPDPSTKFVSSDATPESLYAVLFTSGSTGKP
KGIRIPHSSLASVSDAHAAETGVGPGSRVFQFSAYTFDIGILDVLVTLMRGGCVCVPSDHDRLNNLAGVINALQA
NWVFLTPTVADMLNPADVPSLTTINLGGEAVNKKSSERWQGYTTLNGLYGPAEASICARNADVHNGASTNLGF
PLSSAFWAVEPNDPSRLVPIGCIGELLIQSPILAYGYLNVDAKVAANWLEDMTYDWLPANGPKRAYRTGDLVRR
NPDGTFEYMGRKDNQVKI

>*Trichoderma citrinoviride_mod20_Pheol*

SKQAARRPEHQALYSSEGSMTYSELDQLTTQLAVYLSGLGVRPETIVPFCFEKSIWAIVAMMGILKAGGVFMPLDP
SYPASRRQALIDEVNAQFMIVSPTTAPDSQGMVRNLIELSPSLISRVSTISIAQQSLIKSGPNNAAYVLFTSGSTGKPK
GVIIDHKAISAALLRQREAFSFNDDTRTLQFANFVFDACIAEIFTSLVVGATVCVPTEHERVHNTAAFIREARVNH
AFLTPTFIKTLSPEQIPDMKTVILMGEAPSRGIIDTWADEIDLHNGYGPAEGCVGSTNNTYSSSVKVSVTNVGRSFT
HGLWIVDPDDHNRLMPIGCVGELLLQGSSLARGYINDEDKSRQSFIDEVEWLPSSINIGERRFYKTGDLVRYTPDG
SIEYVSRKDTQVKI

>*Trichoderma citrinoviride_mod3_Aib*

EKQALERPDALAIVGWDRTFTYAELNEAANRLAHHLTQSFSIKADELIHVCFEKSAWHFVAIVAINKAGAGWVP
LDPSHPEQRLRQIASQTRARIVLTSPANSDICARLGLSVIEISPFFDQKLIKSGMNGSAGPDVKITPRNIAYVLFTSGS
TGTPKGLVMEHGSVCTSQTAISQRLGLTADVRMLQFAAFVFDLSIGEIVAPLISGASLYIPDENTRLNDLSNFIQEN
QINWAFLTPAFARTIKPEDVPALELLLLAGEAVSRDVFESWFGKVRLINGWGPAETCVFSTLHEWKSIDESPLTVG
RPVGGFCWIVDPENSEKLAPTGTIGEVVIQGPTLLREYLADAERTRLSTVYDLPAWAPCRESQHWSRFYKSGDLC
YYNPDGTIEFSTRKDTQIKI

>*Trichoderma citrinoviride_mod4_Ala*

ERQTKHTPHAHAVHAWDGSLTYSELDLAANRLANFLIQRHGVKVGDVVHVCFEKSLWYVVSVLAINKAGAA
WVPMDPAHPFQRLQQVASQTVANLALSSSIHGPLCSKLLDTVVEVSSALDEQLKGDEAISDVKPATKVTPNDAV
YLLFTSGSTGVPKGIIMEHASLCTSQRDIAKRLGLTSSVRMLQFSSFVFDVSVGEIMLSLMHGGCVCIPSDHDRLNN
LDGFIRDAQVTWAFLTPSFARTLRPQDVPTLELIVLAGEPVSQDVFDLWFGKARLVNGWGPAETCVLSAIHEWKS
ADESPLTIGRSVGSSAWIVDTENPQRLAPVGCIGEIVMQGPTLLREYLADPVKTASSTLTSLPNWAPRANDKKWG
RFYKTGDLGFYNPDGTIHYSGRKDTQVKI

>*Trichoderma citrinoviride_mod5_Aib*

ARQAELRPEAVAISAWDAELTYSRLDLAANRVANHLIAAYGVKPNDFIHVCFEKSAWHFVAILAINKAGAAWV
PLDPSHPEQRLRQVISQTGAKLVLTSPSNSTLCSGLLENVLEVTSALEQRLAETGSSKAPKVSVTPEHAVYALFTSG
STGTPKGLVMQHGAVCTSQTAIAKRLGLSSDIRQLQFAAFVFDLSIGEIIAPLISGACVCVPSEDIRMNSITEYIRDQ
RINWAFLTPSYVRTLRPKDVPGLELLLLAGEAVPKEILNTWFGKLRLVNGWGPAETCVFSTLHEWKSVDESPLNV

GKPVGGFCWVVDPENPHKLAPVGTLGEVVIQGPTLLREYLADPERTAASSAVAPEWAPQPDSKHWGRLYKSGD
LCSYNPDGTIEFSSRKDTQIKI

>*Trichoderma citrinoviride*_mod6_Aib

ERQALATPEAIAVDSWDGKLTYAQLDQAATRLSHHLIKTYDVASGDLVLLLFEKSLWYIISMIAVNKAGATWVP
LDPSHPMQRLQQVTRQTNAKVILTSSLHFELGEELLDTVVEISQDLDDALTTAGHTLSAPDVMVSPQDSAYVLFTS
GSTGVPKGLVMSHESVCTSQTAIASRLGLHSGVRMLQFSAFVFDVSIGEIYGSLIRGACVVVPSEDIRMGDLTGFM
QEKEVTWACFTPSFVQTLHPADFPTLELVILAGEPSKRQILEDWVGKVKLINGWGPAETCVFSSMHEWKSTTDSPI
TIGKPVAAFAWIVDPDNHHRLAPIGTIGEVVLQGPTLLRGYLDDPVKTEASILKSIPSWAPRRESQHWNRFYLTGD
LCSFNPDGTLEYHGRKDTQVKI

>*Trichoderma citrinoviride*_mod7_Gln

AQQAARDPHHEAIYSSEGTVTYATMDRLSSLLAHHLHALGVRPESVVPFCFDKSAWAIIAMLAILKAGGVFLPLD
PSHPRNRREALVQEVGAEVMIVSPSSSVTCEGLTPTMVELTTQLLEQLSSVYDAFKQSHPKPKPSNAAYVLFTSGST
GKPKGVLMEHSGFATSTLGHGRVYNLGPTSRVFQFSNYVFDGSLGEIFTTLSFGGTVCVPSETERLQEAPTFMRKA
RVNTAMLTPSFVRTFTPDQVPSLQLLVLGGEASSKDLIETWCDRLRLVNGYGPAEACNYATTHDFKPTDSPRTIG
RGFNSACWIVEPTDYNKLTPIGCVGELIIQGNALARGYINDPKRTADSFVTAVNCLPKDSISGPHRFYLTGDLVRY
NSNGEMEYLGRKDTQVKL

>*Trichoderma citrinoviride*_mod8_Aib

EQQAVTRPEAPAIDGWDAKFTYAQLNEAANRLANHLVAEYEIKNDELIHVCFEKSAWFFVAILAINKAGGAWV
PLDPSHPAQRHQQIVSQTKARLALVSASHVSTCVDLVDEVLEVSSTTDEIFRKTESSHRGPARKVSSSNAAYVLFTS
GSTGTPKGLVMEHGSVCTSQTAIVKRLNMTPSVRILQFAAFVFDLSIGEIVAPLITGACICVPSEHDRMNALPEFVR
QNNVNWAYLTPSFIRTLSPKDVPSLELVLLAGEPVSRDILDAWLGKVRLVNGWGPAETCVFSTLHEWQSIDSESP
LTIGRPVGGFTWIVDPQDPQKLAPIGVVGEVVIQGPTILREYLDDPIRTSASTVYNLPAWAPNRGAKWNRFYKSG
DLCSYNADGTIEFVSRKDTQIKI

>*Trichoderma citrinoviride*_mod9_Vxx

VALVRGDALAISGWDKSFTYKEMAEATNRLANHLVNDYGVKVGDVVHVCFEKSAWFIIATLAINKAGAAWST
LDTSHPIERYQKIVSQTESKLALASAVNSHRCAGLLPHVIEVTSELDARLAQNASWSTRGPAVTISPSDAAYILFTS
GSTGVPKGVVIEHASLCTSQTSLSQSLGFHEGFKVLQFSSYSFDAALFEIGSTFLTGACLYVPSWDEQMNELVGYIR
KHGITSTLLTPTLARTIRPEDVPSLDVLLVGGEAPTRDILDIWFGKLRLTNAWGPTECCVIGCIHQWTSVEESPKVI
GRPIGGSCWIVDPDDATRMAPVGTVGEIVVQGRNVFREYLSDPVKTAAATITGLPEWVPKRESAHLDRFYLTGDL
GFINEAGNIEYCTRKDTQVKI

>*Trichoderma endophyticum*-mod1

DLQRAIECNSEEPDIISSCLHQIIEDHALKRPDAMAVHASDLTLTYSQLNLAADRLSRYLIDKYAVQARDLVHVCF
EKSAWFFVSILAINKAGAAWVPLDPSHPLQRHKKVVMQTKAKLALASPNNIPLCADLVENVVEVSATLDEMLIR
RRQGNKNPRTVSPSDACYALFTSGSTGTPKGFVMQHRALCTSQTAVTKRLRMTSDVRVLQFASYVFDVSIGETVG
PWIAGACICVPSEEVRMNGLAEYIRMMKINWVYSTPSFSRTLNPDDIPDVELLLLAGEAVSRDILETWVGKVRLIN
GWGPAETCVFSTLHEYGSLDDSPLTIGRPVGGFCWVVDPENPHRLAPFGTIGEVVIQGPTLLREYLADPAKTKSAT
VKPLPSWAPNRSSDQWNRFYKSGDLCKYNSDGTIAFVTRKDTQIKIRGLRVELGEVEHQIKACLDGVHQVMVDK
IDNEAGASLVAFFCYNTETKIVNTAGGDPTMFLPITNQLKSQIVGMIGHLSVNLPRYMVPSLFIPC

>*Trichoderma endophyticum*-mod11

SQLNDLSLTSNWDVEFSHKVNNEALPEILDTCLHTLIELQAEACPDAMAVQGWDKTFTYHELNRAANRLAHHL
VTEIAWYVAAVLAINKAGAAWGPIDPSHPLQRHKQIVSQTKARVALASPDNVERCSKLVPSVVAVSSSLDSNLSE
KNYDSRSGPDVTVTALHAAYVLFTSGSTGTPKGLIIDHGALCTSQTAVGKRFGFTPKVKILQFTTYVFDFSISEIIAA
MLFGACVCIPSEEIRMNRLKDFVQESGINWLFLTPSFLQTLKPDEVPNVELVAVGGEPLPRALFEEWVDKVRLFNC
WGPSETCVMSAMHEYKSADESNMTIGKPVGGFCWIVDPEDPQILAPAGAVGELIVQGPTLLREYLNDPAKTEASI
LCTRPDWALCPDTEHWNRFYKSGDLCSYNPDGTIQFHSRKDTQVKIRGLRVELGEIEYRIKEALHGALQVAVDVF
KSDTASVLAAYFCFSDATAVISGTDVNDVFMPLTEEVQHRIAAMVGEIKISLPEYMIPTLFIQCQ

>*Trichoderma endophyticum*-mod10

LELAIASNSHTAKIVNSFTHELIDKRAQISPDALAISAWDGELTYSQLTTASNRLAYHLIHTLGIRPDDLVHVCFEK
SVWYFVSIIAINKAGAAWVPLDPSQPEERLLQIAHQTKARVALVSPENTTLCSKLVESVIEITAKLDEELSLSVLHSY
VPRVALSPHSAAYLLFTSGSTGTPKGVVITHMSICTSQIAFADRIGLNSNVRILQFSSFVFDMSILETLGSLFYGACVC
VPSDRERMENITGFINEQRVNWVYLTPSFARTIQPEDVPGVQLLLVGGEAIPQDLFHTWFGKVRLINAWGPTEASI
VSALHEWTSIEESSLVIGRPVGGLCWIVDPDMPYRLAPIGTVGEIIIQGPALLREYLGDPQRTEAALIQLPTWAPRP
DHQNWNRFYRTGDLGYYNSDGNIVFSGRRDTQVKVRGFRIELGDIEHHIRDQLDAICEAAVDVFKTTSGASLIAFI
GFNVKAQQPDTTNGDSSADIFLSLDSHIQAKLSTLLGQLKALLPPYMVPTIFVP

>*Trichoderma endophyticum*-mod12

MQQSHKLNSDVPEIINSCFHQLVEDQATYRPNAMAIRSWDGDFTYAELNQAANRLANHLVQTYDIETNELIHV
CFEKSVWYFISILAINKVGAAWVPIDPSHPVQRLQQVVDQTQAKIVLCSPEYIDLCFSLVESVVQVGPELDNQLFQ
LVNSQQGPASNVSPNNIAYVLFTSGSTGTPKGLVMQHKSVCTSQIAIARRLGLTPEVRMLQFAAFVFDLSIGEIIGP
LITGACLCIPSAHSRVNELPRFIQDMNVNWAYLTPSFVRTLKPTDVPGLELLLLAGEAVPRGVLTAWFGKVRLIN
GWGPAETCVFSTLHEWESIDESSLTIGRPVGGFCWVVDAENPDRLAPFGTIGEVVIQGPTILQEYLADPERTNAST
MPSTEWAPRPDGTNWNRFFKSGDLCRYNADGTLEFSTRKDTQIKIRGLRVELSEVENHIQQALIGVQQVAVDVF
RGESGSNLVAYICFNNETRTAGVQIDKVETPFSSPDESLRNQLVALAGELALALPRYMIPTLFIPC

>*Trichoderma endophyticum*-mod13

LLSPWDLIHAVESNRLNPSSETCAHWLIQENIQSRPNDIAIASWDAELTYSQVGTFASRLAVKLQERGVGPETLVL
LCFPKSAWAVIAMVAIEMAGGAFVPLDPAAPAARLQGIIDDTHAILAIASPSTGTVLEGLDVEIMLVDQEMLSAL
PDPTHPVVSNVQPQHASVILFTSGSTGKPKGMVIQHNNLCSSSDAYGDKLNIGPGTRVFQFSAYTFDVGILDCLVS
LMRGACLCIPSNHDRMNNLAGAMRSLKANWVFLTPTVADLLSPTDVPDLKVVCLGGEAISKKCADRWVNHVE
LHGLYGPAEASICAWNPTVGKFDRSTNLGRPISSAFWVVEPSNIRQLVPVGCIGELLIEGPMLARGYLNVNAEVA
ANWINDVDWLPGEKKRVYRTGDLVRRNADGTFDYMGRKDTQIKLHGQRVELGEIESQIHHFLPDNMAAMVDI
LKADDENAHDILLAFLWYTEEDTTQSQSLHLMESLPEKSQRFICHLDSSLAETLPSYMIPSSYLIFEGKPE

>*Trichoderma endophyticum*-mod2

QRSMKWNDHEVVIEEHCMHDVISRRTESCPNDEALYSSEGSLTYAALERLSDLVAYQLLQYNVQPETIVPFCMEK
SIWVVVAMVGILKAGGALMPLDPSHPESRRLALIQEVNAKVIITSPSTALSCEGMAQHTVQVSSSLLTHAPKTTKS
YRELSNYKKPEPHNAAYVLYTSGSTGKPKGLVMPHSAACTSLLRHPEKFSINKSTRTFQFSAYVFDVCIEEIFVSLFV
GATICVPTETERVGNTSRFMTEARVTWTCLTPSFIGTLDPDTLPTLQTLCIGGEAPTRDILSKWHGRAEIINAYGPA
EACVDVAGHVFKSRDDSPTNIGRPFAHKLWIVEPDNHNRLTPIGCIGELVIEGHAIARGYINNEEKTKESFVDDLE
WLSSATPSDRPRVYKSGDLARFNPDGTIEFFGRRDTQVKIRGQRLELGEIEYNIRAKLADVQHTVVDLIEREAGKM
IIAFITFKNYLCDTMADIADPYGNFIKDVKLTSSFQDLMLELRRVLPSYMIPSIIFPLR

>*Trichoderma endophyticum*-mod14

DVEQAIERNNYSIEPIESCVHELISIQAAKNPQQEAIYSTSGSMTYRKVDQLSNQLASHLIRLGVKSETIVPFCYEKS
VWSIIAMLGIIKAGGVFLPLDPSHPLSRRQALIDETSAQYMIVSPTTAEECKGMTKNNIELSPKFFSKKKRSSTARKF
KPMKPRPGSAMYILFTSGSTGKPKGVIIEHKPISSFLVKLCEVAQFNGNSRILQFSSYGFDASIFEIFAILISGGTVCVP
TETERMQHTSTFIEEAKVNTAILTPTFVKTLSPESVPSLKTLFLGGEAPSKETIDLWRQHVSLWNAYGPTETCITSTL
QHYTDATTLATSIGRGFAHHCWVVNPENHHELTPIGCVGELLVQGDSLSRGYINDQEKTNNAFINHVEWLPAN
VDVGKRRFYKTGDLVRYNPDGSFNYLGRKDTQVKIRGQRIELGEIEYQIKLHLPDLKHAAVDIIRHDTHESLVAFV
NFSGDKGPSEVAQVQLVSHNDELQELFSQLVANLTTVLPSYMVPKYFIAVESMP

>*Trichoderma endophyticum*-mod3

IEFARAANPNPPNVVDTCIHTLIEQRTQISPDSAAVCSWDADFTYSELDENANRLANYLMNVLKVKAGDLVMV
CFDKSAWFVVAILAINKVGAAWVPIDPSHPTERHQSIVKQTRSTLALCTPSNSLKCSQLVATVLEVNREFISELRGI
EAYAGKPAVKVSPNDVSYIIFTSGSTGVPKGVVIQHRAICSSQVALSQRLSMHAGVRMLQFSSFVFDASLFEILSPLI
SGACVCIPSWDMQMNSLPAFIRDTNVTWAFLTPSLAGILSPQDVPSLELITVGGEAPSKEVFDIWLGKVRLFNVW
GPTETTVIATVHEVKSTSTQGSHLTIGQPICGNCWIVDPEDPTKLAPVGTVGEIVVQGPNLLREYLADPEKTAAAT
VTSLPQWTPNRELWNRFYRSGDLAVYNSDGTIKYCSRKDGQIKIRGLRIELGEIEHHIRKNMDTQGQVAVEALRS
EAGTNLVAFICQNSDTIPASMTNNMTSEDIVLPLTDELKSSLGALRSSLISSLPGYMVPTFFVPC

>*Trichoderma endophyticum*-mod4

EKAVSFNPEMPEVVENCFHQLFESQALETPEATAICAWDRSLTYAELDNAANRLAHYLMAQHTVRLDELIHVCF
DKSAWFFVSILAINKAGAAWVPLDPSHPPERLRQIVTQTRAQVVLTSESHAALCGRLVPLVIKVSDSLDRQLSSND
GYSSSSPVTKVSPNNAVYVLFTSGSTGTPKGLVMEHQSVCSSQKAITKRLGLHSKVRMLQFAAFVFDLSIGEIIAPLI
SGACVCVPSEQARMNDITGFIQETGVNWACLTPSFIRVLKPEDVPTLELLLLCGEVTPRDVFNTWVGKVRFISGW
GPAETCVFSTLHEWKSNTESPLTIGKPVGAFCWVVDPERPDQLAPIGTVGEVMLQGPTLLREYLNDPERTKSSVIT
SLPNWALQRAPQWTRFYKSGDLAMYNPDGTIEFCSRRDTQVKIRGLRVELSEVEHRIRESLEGIRQVAVDVSTSDG
GSNLVSYLCFSEEIRSSSNAQANLDDIFLPVTAELQPLLAAMIGQLKVILPSYMIPTLFIIC

>*Trichoderma endophyticum*-mod5

VGDWDIKHAVNSQFPMSSTQSCTHWLIQDAIRAYPDSPAVASCDGELTYAELGTLVSRLSTKLQQLGVGPEVLVP
VCFPKSIWAVVTMIAVEMAGGAFVPLDPKAPQSRLKSILDDIKATIALTSPCCESTMKDLDVSTVAIDGPFLDTLPE
ASVSSQHSGSPSDAAVVLFTSGSTGRPKGMAIQHDALSSSAAAYGRNLQIGPGTRVFQFSAYTFDVGVLDTLVTL
MRGGCLCVPSDHDRLNDLPGAINKTKANWVFLTPTVADLLNPADVPTLRVVCLGGEAISQKTANRWKYAELH
GLYGPAEASICAWNSTVGRSGKSTNLGRPLSSAFWVVEPSDIHSLVPVGCIGELLIQGPMLARGYLNVRAEQAAN
WLEDFTADWLPYGFPNRAYRTGDLVRRNADGTYDYIGRKDTQVKLHGQRVELGEIESQIQKALPAEMTAIVDIV
KSHVLAFLWYTHETAPNEQSSLKLISTLSETQKILISDIDMALSTALPPYMIPSSYLIFDGKPGQTT

>*Trichoderma endophyticum*-mod7

EYDFALAANGNPPVTVNRCLHSLIEEEAKKHPDTIAVQGWDVAFTYQYLDDCANRLAHHIINNFHVKSGDIVH
VCFDKSAWYIVAILAINKAGATWSPLDPSHPIQRYEQILSQTGSSLILTSTTNTAKCATLARIVLEISSELNETLETGA
NVHRPDVNVSPADAAYIIFTSGTTGMPKGVVIEHTSVCTSQMALARRVGITDATRMLQFASFVFDASIFEIFAPLFV
GGCNIWSKLLLRPEEIPNLEILVVGGEAASSDIIQVWAGKLRVFNAWGPTEICVIGSVHEWTDSNDSPMTLGRPIL
GNWWIVDPNNPMRLVPTGCVGEIVYQGPTIFREYLANPSQTEEAIVSSLPVWAPRRDMRGWSRFYRTGDLGCYN
PDGTMEYFGRKDTQVKIRGLRIELHEIEHQIFAHFPGVRQVAVDVMKREEGSHLVAYYCFSNDTIPSHLVADSEN
NNTFLPLRDDILPQIQDLITHLGDILPPYMVPKTFI

>*Trichoderma endophyticum*-mod8

VEQSMKFNPEVPEIMDTCVHTLIEAQAHREPNALAISAWDGDLTYRQLNESANRLAHYLVKNYNVQPDTLIHV
CFEKSVWHFVAIIAINKAGAAWVPLDPSYPEQRLRQVISQTRSTLVLTSSRNAKLCSNLINKVLRIDVELDKRLILTE
DGHKGPTVAVTHRHAAYVLFTSGSTGKPKGLVMEHGAVCTSQTAIAKRLNLTSDVRMLQFAAFVFDLSIGEIIGP
LISGACLYIPSEETRMNGLRQYVNETGVNWAYLTPAFVRTFVPEDMPGLKLLLLAGEAVPREVLSTWFGKVRLVN

GWGPAETCCFSTLHEWSSVDESPLVVGRPVGGFCWIVNPDDPQKLTPTGTLGEIVIQGPTILREYLADVERTKDST
VYALPKWAPRPDSEHWNRFYKSGDLGYYNPDGTIVFSSRKDTQIKIRGLRVELGEVEHYIHETLEGVCQVVVDVY
QKEGRSNLVAYICFNDEMKNPSADEEFTAAALFASITDDMRQRIAIMLGELRITLPQYMIPTLFI

>Trichoderma endophyticum-mod9

SWDLQQAIDASRLRPATESCTHWLIQDKIKEQPEETAITSWDGDLTYAQLGAHATCLAAKLQDFGIGPESLVPICF
HKSKWAVVSMVAVQMAGGAFIPLDPGAPPARLQGILQDTGAALAISDPSSQAALQSLGVEVITVDDKAISKLPSS
VALQCEVQPANASVVLFTSGSTGKPKGMIIQHNSLCSSSDAYGSDLKIGLGTRVFQFSAYTFDVGVLDCLVSLMRG
ACICIPSDHDRLNNLAAAITASQANWVFLTPTVADLLHPVDVPTLKTVCLGGEAISKKCAERWVNHVDLHGLY
GPAEASICAWNSMVGKSGRSTNLGHPISSAFWVVDVNDPKSLVPLGCIGELLIEGPMLARGYLNVSADVAANW
MEGVDWLPGNNQPRRIYRTGDLVRRNADGTFDYIGRKDTQVKLHGQRVELGEIEAWINEFLPDNMAAIVDVVK
DKDGPDSLLAFLWYTEEASNSPARLMDVVSDEARAAILHLDTSLSTILPPYMIPSSYLLFGGKPEQT

>Trichoderma parareesei_mod1_Aib

ERQAKTAPDAPAIHAWDMDLSYSQLDSAANRLAHHLVKSCGVKDQDFVHVCFEKSAWFFVSVIAVNKVGATW
VPLDPSHPLQRQQQVVSQTKATLALASPSNVEMCSQLVNTVVEVSWALDAKLSKTEESSYGPVRNVSPDNAAYV
LFTSGSTGTPKGLVMQHRAVCTSQTAITKRLKMTSSVRMLQFASFVFDLSIGEIVGPWVVGGCLCVPSEETRMNN
LVDFINTMKVNWAYLTPSFTRTLNPDDVPGLDLLLFAGEAVGRDVFEAWLGKVRLINGWGPAETCVFSTLHEW
KSFEESPLTVGKPVGGYCWIVDPHDPQRLAPVGTLGEVVIQGPTVLREYLADPTKTEASLVRSLPEWVPNRTAAH
WDRFYKSGDLCRYNADGTIEFGSRKDSQVKI

>Trichoderma parareesei_mod10_Aib

EKQAVAQPDAPAVLSWDHSFTYKQLNEASNRLAHLLVNKYNVKPNDLVHVCFDKCAWHFVAITAINKVGAA
WVPLDPSHPEMRLRQIVSQTRATLTLVSSSNASLCSSLTERVIEVNAGLDNELLAVESGEYGPSVDVSSHSAAYVLF
TSGSTGVPKGFVMEHGSVCTSQIAIARRLGLGPNVRMLQFAAFVFDLSIGEIVGPLISGACICVPSEDTRMNGIVAFI
NETKVTWAYITPSFARTIKPSEVPHLELLLLAGEAVPRDVFATWFGSTVRLINGWGPAETCVFSTLHEWKSADESP
LTVGRPVGGFCWIADPEDPSRLAATGTLGEIFIQGPTILREYLSDVERTEATVIKSLPEWAPFRNEPGWDRMYKSG
DLGFYNPDGTIEFSSRKDTQVKI

>Trichoderma parareesei_mod11_Gly

ERQVKQRPDSPAVDAWDGRLTYSQLDHAANRLAHLLVDNYAVQVGDIVHVCFEKSMWHIVAILAINKAGAA
WAPLDSAHPFQRLQAVAKQTGAKLALASTANAGLCEQLVDRVIEVSAALGETFSSNDTNSEKGPQVDVSPLDA
AYILFTSGSTGTPKGIVMQHGALCTSQTDISRWLGLDHTVRMLQFSSFVFDVSVGEIMEPLMNGACVCVPSDHMR
LNSLDVFVRDFNVTWAYLTPSFTRTLKPEDFPGLELLLLIGEAVTQDVLDTWFGLPNTRFVNAWGPAETCVFSTL
YDWQSNTESPLTIGQAVGAYVWVVDAENPQRLAPTGCLGEIVVQGPPLLREYLADPSKTAAATVTGLPEWAPRR
ESTKWNRFYRTGDLGFYSHDGLLHYASRKDTQVKI

>Trichoderma parareesei_mod12_Aib

EEQSRLHPHAPAIAAWDGNFTYDELNKAANKLAHHLVNVYGVKPNDFVHVCFEKSAWYIVSILAINKAGATW
VPLDPSHPEQRWQSIIGQTKATLALVSRGSVAPLSRLIDNVVEIDSNLDSLLPEHDAARSPNVSISPSTAAYVLFTSG
STGTPKGFIIEHKSISTSQKAFNKRLRYHKNVRVLHFAASVFDLSIAEIIMSLLNGACLCVPSEHTRLNGIIDFIRDM
KINWLYLTPSFLRTIDPSEVPNVELILLAGEAVPREVFETWYGRVRLLNGWGPAETCVTSTIHEFEFADDSPLTIGRP
VGGFCWIVDPENPQLLAPTGTVGEVVIQGPTLLREYLDNPEKTQETTVYELPDWAPRPDEKNWSRFYKTGDLCFY
NPDGTIEFSSRKDTQVKI

>Trichoderma parareesei_mod13_Aib

EEQARIRPHAPAICAWDGEMSYSQLNAAANKLAHHLFTAGVKSEDLVHVCFEKSLWFFVSIVAVNKVGAAWVP
LDSSHPEQRLRQVVSQTRAQFALSSPTNAALCGSLVDKVIEVSQGLIDSFPYDGANGPAIKVPSSNAAYVLFTSGST
GTPKGLVMQHQAVCTSQTAIAKRLRLTPDVRILQFAAYVFDLSIGEIVAPLIHGACVCVPSEEMRMNGLKEFIRD
ASINWAYLTPSFVRTLRPEDVPDLDLLLLAGEAVGRDILDTWFGKVRLVNGWGPAETCVFSTLHEWASIDESPLT
VGTPVGGHCWIVDAEDSSKLAPIGCLGEVVLQGPTLLREYLADPQRSKEAIITSLPSWAPKQDSQPWSRFYKSGDL
CYYNPDGTLEFYSRKDTQVKI

>Trichoderma parareesei_mod14_Pro

DRIEKQPSDPAIASWDGDLTYEQLGVLASRLAWKLQGLGVGPESLIPLCFPKSTWAVVAMVAVEMAGGAFVPLD
PKAPVARLRGIIEDTKSTLAVASPSCQDALHEIGIDVLAVDEALLLELSDPVEGIQSKAGPKNASVVLFTSGSTGKP
KGMVIQHNSLCSSGNAYGQDLNIGPGTRVFQFSAYTFDVGVLDCLVSLMRGATICIPSDHARLNDLAGAMTATK
ANWVFLTPTVADLLSPADVPYLKVLCLGGEAISKKCADRWINYTELHGLYGPAEASICAWNPSVGKSGRSTNLG
RPISSAFWVVEPNNHKQLVPVGCIGELLIEGPMLARGYLNVSADVASNWMDNVDWLPGSDKKRVYRTGDLVRR
NADGTFEFMGRKDTQVKL

>Trichoderma parareesei_mod15_Vxx

EQRALMNPNAEAVSAWDARFTYAELDRFANILANHLIQSMGVQVGDLVHVCFEKSAWYVVSILAINKAGAAW
IPLDPSHPAERHQQVVGQTRSRLALTSPANAAKCANLVADVLEVTRGLLDDLETQVNDTRPVTSVGPQDVAYIL
FTSGSTGVPKGVVMEHGALCSSQTSISKRLGYAPGVRMLQFASFVFDACIGEIIAPLISGSCVCIPSWETQMNSLTSY
IHEENVTWAMLTPSFARTMDPSEVPCLELLILIGEAVSRDVFELWFGKLRLLNGWGPTETCVFGALHEWQSIDES
QMTIGQPVGGYCWIVDPEDPQKLAPTGTFGEVVIQGPNLLREYLADEVKTASSTVTVLPEWAPNKHLRHWNRFY
KTGDLAMYNPDGTIQYYSRKDTQVKI

>Trichoderma parareesei_mod16_Aib

ERQAEVRPDAMAIRAWDAELTYREFNRAANRLANHLTASYDIKPDDLIHVCFEKSAWFFVSILAINKSGAAWVP
LDPSHPEQRLRQVVSQTRARIALTSPSNRDLVTGLVESVVTVDSQLDVQLSKVDEHSQKGPETAVSSDNAVYVLFT
SGSTGTPKGLVMQHGSVCTSQTAIVQRLGLTPDVRMLQFAAFVFDLSIGEIIAPLLTGACLCIPSDHTRMNGLTQYI
RDTGINWAFLTPSFIRTINPAEVPGLELVLLAGEAVPRDVLTTWFGKVRLVNGWGPAETCVFSTLHEWQSVNESP
LTVGRPVGGFCWVVDPEDPHRLAPTGTLGEVVIQGPTLLREYLSDPERTQASTVYDLPKWAPRPDSRHWNKFYK
SGDLCYYNQDGTIEFSTRKDTQIKI

>Trichoderma parareesei_mod17_Vxx

EAQAKQTPDLPAVWAWDGQLTYRQLNEAANRLAHYLIREHNVQVEDLVHICFEKSVWHWVSVLAINKAGAV
WVPLDPSHPEQRLRQVASQTRSTLALASDTTKSLLSHIIERVVEVSPALFERIDVRLGEKEPQAPVSASNAAYILFTS
GSTGTPKGLVMTHGAFATSQTAIKKRMGTGTHTRALQFASYVFDMSVGEGFVQLISGACIFIPSEHTRMNGLKDF
ITEHRINSLWLTPSFIRTLSPEQVPTVDFVFLAGEAIPRDVFTTWCTKVRLWNGWGPAETCVVSSLHEFTSLDESPL
TIGRPIGGYCWIVDPTDHTKLAPIGTMGEVVIQSPTILREYLADIERTKASTVYELPEWAPYRDQAPWSRFFKSGDL
ASYNPDGTLEFASRKDTQVKI

>Trichoderma parareesei_mod18_Gln

SQHAMGSPHHEAVYSSEGSLTYGELDHLTDIFATHLSSLGAGPETVVPFCFEKSMWAVVAILSILKAGAAFVPLDP
SHPTSRREALVKEVGARVLVASSSAIASCKGMVEHVVELSPSVMAKLAASVTPRILPKVGPRNTAYVLFTSGSTGK
PKGVVMQHGSFSSTTIGYGKVYNLSPLSRVFQFSNYIFDGSLGEIFGPLAFGGTICIPSEDERLGSAPAFMNAAKVN
TAMLTPSFVRTFTPAQVPHLTTLVLGGEAASKSTLEMWVDRVTLYNGYGPAEACNYATTHVFKSSSESPRIIGSGF
NGACWVVEPDNHNILAPIGCTGELVLQGHALARGYLNDKAKTEQSFVSDIGSLPSSSLHEPKRFYLTGDLVRYNS
SGELEYLGRKDAQVKL

>Trichoderma parareesei_mod19_Gln

DNAQRDPNHEAIFSSEGSMTYAALDRLTDVLASHLCQLGVGPETIVPFCFEKSMWAVVAIVGILKAGGVFVPLDP
SHPINRREALVKEVGAQIIIASESAAASCAGMAPRIVQLSSDFMARLSAPSQTLVEPRRPSPSNAAYVLFTSGSTGKP
KGVVMEHSALTTSTIGYGRVYELSPASRVFQFSNYIFDGSLGEILATLTFGGTVCVPDEVERLQDTPGFVRKAQINT
AMLTPSFVRTFTPDQVPSLKTLILGGEPAGRDILDAWCDRVKLINGYGPAEACNYATWHPFSSSRDSARVIGKAF
NSSCWVVEPNDHHALTPVGCVGELAIQGHVLARGYINDSERTRSSFVTEIASLSPVIAGPQRFYLTGDLVRYCSDG
SLEYLGRKDTQVKL

>Trichoderma parareesei_mod2_Ala

KQIEAQPDAPAFDSWDAKLTYREVGLLATRLAAKLQSLGVVSDVMVPISFPKSTPAMITMVAIQMAGGALVPLD
PAAPLARLQSIVSDTQAKLIVTHPSLKEKLQELGVDLLIVDLSMLDKLPDPSTRFISSDATPESLYAVLFTSGSTGKP
KGIRIPHSSLCSVSDAHAAETGVGPGSRVFQFSAYTFDIGILDVLVTLMRGGCVCVPSDHDRLNNLAGVISALQAN
WVFLTPTVADMLNPADVPCLTTINLGGEAVNKKSSERWQGYTTLNGLYGPAEASICARNADVHNGASTNLGFP
LSSAFWAVEPNDPSRLVPIGCIGELLIQSPILAYGYLNADAKNNANWLEDMTYDWLPANGPKRAYRTGDLVRRN
PDGTFEYMGRKDNQVKI

>Trichoderma parareesei_mod20_Pheol

SKQVARRPAHQALYSSEGSMTYSKLDRLTTQLAVYLSSLGVRPETIVPFCFDKSIWAIVAMIGILKAGGVFMPLDPS
YPASRRQALIDEVNAQFMIVSPTTAPDSQGMVQNMIELSPSLIAFFSTIDTGDQSFIKSGPNNAAYVLFTSGSTGKP
KGVVIDHKAISAALLRQREAFSFNDDTRTLQFANFVFDACIAEIFSALVAGATVCVPSEHERVHNTAAFIREARVN
HAFLTPTFIKTLSPEQIPGMKTVILMGEAPSQEIIDTWADEIDLHNGYGPAEGCVGSTNNTYSSSVKVSVTNVGRSF
THGLWIVDPDNHNRLMPIGCVGELLLQGSSLARGYINDEEKSRQSFIDQVEWLPANVNVGERRFYKTGDLVRYT
PDGSIEYVSRKDTQVKI

>Trichoderma parareesei_mod3_Aib

EKQALERPDAPAVVGWDRVFTYAELNEAANRLAHHLTQTFAIKADELIHVCFEKSAWHFVAILAVNKAGGGW
VPLDPSHPEQRLRQIASQTRGRIILTSPANSDICARLGLSVIEISPFFDQKLIESGMNSSTGPDVKVTPRNIAYVLFTSG
STGTPKGLVMEHGSVCTSQTAISRRLGLTADVRMLQFAAFVFDLSIGEIVAPLISGASLHIPDENTRLNDLPNFIQK
NQINWAFLTPAFARTLKPEDVPALDLLLLAGEAVSRDVFESWFGKVRLINGWGPAETCVFSTLHEWRSLDESPLT
VGRPVGGFCWIVDPENPDKLAPTGTVGEVVIQGPTLLREYLADPERTKLSTVYDLPAWAPRRESQHWSRFYKSGD
LCYYNPDGTIEFSTRKDTQIKI

>Trichoderma parareesei_mod4_Ala

ERQTKQTPHAHAVHAWDGSLTYSELDLAANRLANFLIQRHGVKVGDVVHVCFEKSLWYVVSVLAINKAGAA
WVPMDPAHPFQRLQQVASQTGAKLALSSAIHSPLCSKLLDTVVEVSSNLDEQLKSDETIGHVKPSTKVTPNDAVY
LLFTSGSTGVPKGIIMEHASLCTSQRDIAKRLGLTSSVRMLQFSSFVFDVSVGEIMLSLMHGGCICIPSDHDRLNNL
DGFIRDAEVTWAFLTPSFARTLRPQDVPSLELVVLAGEPVSQDVFDLWFGKARLVNGWGPAETCVLSAIHEWKS
ADESPLTIGRSVGSFAWIVDAENSNRLAPVGCIGEIVMQGPTLLREYLADPAKTASSTMTSLPNWAPRANDKKW
GRFYKTGDLGFYNPDGTIHYSGRKDTQVKI

>Trichoderma parareesei_mod5_Aib

ARQAELRPDAVAISAWDAELTYGQLDLAANRLANHIITAYGVKPNDFIHVCFEKSAWHFVAILAINKAGAAWV
PLDSSHPEQRLRQVVSQTSAKLVLTSPSNSTLCAGLLGNVLEVTLALDQKLAATLGSQAPKVAVAPEHAVYALFT
SGSTGTPKGLVMQHRAVCTSQTAIAKRLGLSSDIRLLQFAAFVFDLSIGEIIAPLISGACVCVPSEDVRMNSITEYIR
DQRINWAFLTPSYVRTLRPKDVPGLELLLLAGEAVPKEILNTWFGNLRLVNGWGPAETCVFSTLHEWKSVDESPL
TVGKPVGGFCWVVDPENPHKLAPVGTLGEVVIQGPTLLREYLADPERTAASSATAPDWAPQPDSKHWGRLYKS
GDLCSYNPDGTLEFSSRKDTQIKI

>Trichoderma parareesei_mod6_Aib

ERQAISSPDAIAIDSWDGKLTYAQLDQAATRLSHHLIKTYHVAPGDLVLLLFEKSLWYIISTIAVNKAGAAWVPLD
PAHPMQRLQQVTRQTKAQVILASSLQFELAQELLDTVVEISQTLDDALTTAGSTLRTPDVTVSSRNKAYVLFTSGS
TGVPKGIVISHGSVCTSQTAISSRLGLHSGVRMLQFSAFVFDVSVGEIYGSLIRGACVVIPSDEIRMNDLTSFMLEKE
VTWACFTPSFIETLHPADLQNLELVILEGEPSKRHILEEWFGKVKLINGWGPAETCVFSSMHEWASATESPVTIGK
PVACFAWIVDPDNHHRLAPIGTVGEIVLQGPTLLCEYLDNPVQTQASILKSIPSWAPRRESQQWNWFYLTGDLGS
YNPDGTLAYHGRKDTQVKI

>Trichoderma parareesei_mod7_Gln

AQQAARDPHHEAIYSSEGTVTYATLDRLSSLLAHHLHALGVRPESVVPFCFDKSAWAIIAMLAILKAGGVFLPLD
PSHPRNRREALIEEVGAEVMIVSPSSSVTCEGLTPTMVELTTPLLEQLSSTYDAFQQIHPKPKSSNAAYVLFTSGSTG
KPKGVLMEHSGFATSTLGHGRVYNLSPTSRVFQFSNYVFDGSLGEIFTTLSFGGTVCVPSETERLQEAPTFMRKSRV
NTAMLTPSFVRTFTPDQVPSLQLLVLGGEASSKDLIETWCDRLRLVNGYGPAEACNYATTHDFKPTDSPRTIGRGF
NSACWIVEPTDYNRLTPIGCVGELIIQGNALARGYINDPKRTADSFVTAVDCLPRDMISGPHRFYLTGDLVRYNSN
GEMEYLGRKDTQVKL

>Trichoderma parareesei_mod8_Aib

EQQAVVRPEAPAINGWDAKFTYAQLNEAANRLANYLVAEYEINNDELIHVCFEKSSWFFVAILAINKAGGAWV
PLDPSHPAQRHQQVVSQTKARLALVSTSHISTCVDLVDDILEVSSTTDELLRKSQSSRHGPARKVSSSNAAYVLFTS
GSTGTPKGLVMEHGSVCTSQTAIVKRLNMTPSVRILQFAAFVFDLSIGEIVAPLITGACICVPSEHDRMNALPEFVR
QNNVNWAYLTPSYIQTLSPQDVPGLELVLLAGEAVSRDILDAWFGKVRLVNGWGPAETCVFSTLHEWRSKDEES
PLTIGRPVGGFTWIVDPENPQKLAPIGVPGEVVIQGPTILREYLDDPVRTSESTVYNLPAWAPNRGTKWNRFYKSG
DLCSYNADGTIEFISRKDTQIKI

>Trichoderma parareesei_mod9_Lxx

EVALVRGDAPAISGWDKSFTYKEMTEATNRLAHHLVNDCGVKVGDIIHVCFEKSAWFIIATLAINKAGAAWSTL
DTSHPIERYQKIVSQTGSKLALASAANSYRCVGLLPHVIELTSELDARLAQNASWSSCGPTVTVSPSDVAYILFTSG
STGVPKGVIIEHATLCTSQTSLSQALGFHEGVKTLQFSSYSFDAALFEIGSTLLTGACLCVPSWDEQMNELVGYIQK
QEITSTLLTPTLARTIRPEDVPSLDVLIVGGEASTRDILDIWFGKLKLINAWGPTEACVIASTHPWTSVDESPNVIGR
PIGGSLWVVDPDDATRMAPVGTVGEIAVQGRNLFRGYLSDPVKTAAATVTGLPEWVPKRDSSAHWDRFYLTGD
LGFMNEAGNIEYCTRKDTQVKI

>Trichoderma reesei_mod1__Aib

ERQSKANPDAPAIHAWDMELSYSQLDRAANRLAHHLVKSCGVKDQDFVHVCFEKSAWFFVSVIAVNKAGATW
VPLDPSHPLQRQQQVVSQTKATLALASPSNVEMCSELVNTVVEVSSALDEKLSKTEESSYGPVRNVSPDNAAYVL
FTSGSTGTPKGLVMQHRAVCTSQTAITKRLEMTSSVRMLQFASFVFDLSIGEIVGPWVVGGCLCVPSEETRMNNL
VDFINTMQVNWAYLTPSFTRTLNPDDVPGLDLLLFAGEAVGRDVFEAWFGKVRLINGWGPAETCVFSTLHEWK
SFEESPLTVGKPVGGYCWIVDPHDPQRLAPVGTLGEVVIQGPTVLREYLADTTKTEASLVRSLPEWVPNRTAAHW
DRFYKSGDLCRYNADGTIEFGSRKDSQVKI

>Trichoderma reesei_mod10_Aib

EKQAAAQPDAPAVLSWDHSFTYKQLNEASNRLAHLLVNKYHVKPNDLVHVCFDKCAWHFVAITAINKVGAA
WVPLDPSHPEMRLRQIVSQTRATLTLVSSSNASLCSSLTERVIEVNASLDNELLAVESGEYGPSVDVSSHSAAYVLF
TSGSTGVPKGFVMEHGSVCTSQIAIARRLGLGPNVRMLQFAAFVFDLSIGEIVGPLISGACICVPSEDTRINGIVAFI
NETKVTWAYITPSFARTIKPSEVPHLELLLLAGEAVPRDVFATWFGSTVRLINGWGPAETCVFSTLHEWKSADESP
LTVGRPVGGFCWIADPEDPSRLAATGTLGEILIQGPTILREYLSDVARTEATVIKSLPEWAPFRNEPGWDRMYKSG
DLGFYNPDGTIEFSSRKDTQVKI

>Trichoderma reesei_mod11_Gly

ERQVQQRPNSPAVDAWDGRLTYSQLDHAANRLAHLLVDDYAVQVGDIVHVCFEKSMWYIVAILAINKAGAA
WAPLDSAHPFQRLQAVAKQTGAKLALASTANAGLCKQLVDRVIEVSAALDEKLSSNAVSSGKGPQVNVSPLDA
AYILFTSGSTGTPKGIVMQHGALCTSQTDISRWLGLDHTVRMLQFSSFVFDVSVGEIMEPLMNGACVCVPSDHMR
LNSLDVFVRDFNVTWAYLTPSFTRTLKPEDFPGLELLLLIGEAVTQDVLDTWFGLPNTRFVNAWGPAETCVFSTL
YDWQSNTESPLTIGRAVGAYVWVVDAENPQRLAPTGCLGEIVVQGPPLLREYLADPSKTAAATVTELPEWAPRR
ELTKWNRFYRTGDLGFYSHDGLLHYASRKDTQVKI

>Trichoderma reesei_mod12_Aib

EQQASLRPQAPAIAAWDGNLTYDELNKAANKLAHHLVNVYGVKPNDFVHVCFEKSAWYIVSILAINKAGATW
VPLDPSHPEQRWQSIISQTKATLALVSPGSVGPLSRLIDNVVAVDSNLDSLLPEHDAARGLSVSISPSTAAYVLFTSG
STGTPKGFIIEHRSICTSQKAFNKRLRYHENVRVLHFAATVFDLSIGEIIMSLLKGACLCVPSEHTRLNGIVDFIRDM
KINWLYLTPSFLRTIDPSQVPNVELILLAGEAVPREVFETWYGRVRLLNGWGPAETCVTSAIHEFESADDSPLTVGR
PVGGFCWIVDPENPQLLAPTGTVGEVVIQGPTLLREYLDNPEKTQETTVYELPDWAPRPDEKNWSRFYKTGDLCF
YNPDGTIEFSSRKDTQVKI

>Trichoderma reesei_mod13_Aib

EEQARIRPDAPAICAWNGEMSYSQLNFAANKLAHHLLNAGVKSEDLVHVCFEKSLWFFISIVAINKVGAAWVPL
DSSHPEQRLRQVVSQTRAQFALSSPTNAALCGSLVDKVIEVSQGLIDSFPYDGANGPAIKVPSSNAAYVLFTSGST
GTPKGLVMQHQAVCTSQAAIAKRLRLTPDVRILQFAAYVFDLSIGEIVAPLIHGACVCVPSEEMRMNGLKEFIRD
ASINWVFLTPSFVRTLRPEDVPNLDLLLLAGEAVGRDILDTWFGKVRLVNGWGPAETCVFSTLHEWASIDESPLT
VGTPVGGHCWIVDAEDSSKLAPIGCLGEVVLQGPTLLREYLADPQRSKEAIITSLPSWAPKQDSQPWSRFYKSGDL
CYYNPDGTLEFYSRKDTQVKI

>Trichoderma reesei_mod14_Pro

DRIEKQPSDPAIASWDGDLTYEELGVLASRLAWKLQGLGVGPESLIPLCFPKSTWAVVAMVAIEMAGGAFVPLDP
KAPVARLRGIIEDTKSTLAVASPSCQDALHEIGIDVLAVDEALLLELSDPVEGIQSKAGPKDASVVLFTSGSTGKPK
GMVIQHNSLCSSGNAYGQDLNIGPGTRVFQFSAYTFDVGVLDCLVSLMRGATICIPSDHARLNDLAGAMTATKA
NWVFLTPTVADLLSPADVPYLKVLCLGGEAISKKCADRWINYTELHGLYGPAEASICAWNPSVGKSGRSTNLGR
PISSAFWVVEPNNHKQLVPVGCIGELLIEGPMLARGYLNVSADVASNWMDNVDWLPGSDKKRVYRTGDLVRR
NADGTFEFMGRKDTQVKL

>Trichoderma reesei_mod15_Vxx

EQRALMNPNAEAVSAWDARFTYAELDRSANILANHLIQSRGVQVGDLVHVCFEKSAWYVVSILAINKAGAAWI
PLDPSHPAERHQQVVGQTRSRLALTSPANAAKCANLVANVLEVTRGLLDDLETQVNHARPVTSVGPQDVAYIL
FTSGSTGVPKGVVMEHGALCSSQTSISKRLGYAPGVRMLQFASFVFDACIGEIIAPLISGSCVCIPSWETQMNSLTSY
IREENVTWAMLTPSFARTMDPSEVPCLELLILIGEAVSRDVFELWFGKLRLLNGWGPTETCVFGALHEWQSIDESQ
MTIGQPVGGYCWIVDPEDPQRLAPTGTFGEVVIQGPNLLREYLADEVKTASSTVPVLPEWAPNRHLRHWNRFYK
TGDLAMYNPDGTIQYYSRKDTQVKI

>Trichoderma reesei_mod16_Aib

ERQAEIRPDAMAIRAWDAELTYREFNRAANRLANYLTASYDIKPDELIHVCFEKSAWFFVSILAINKSGAAWVPL
DPSHPEQRLRQVVSQTRARIALTSPSNRDLVTGLVDSVVTVDSQLDVQLSKVDEHSQKGPETAVSSDNAVYVLFT
SGSTGTPKGLVMQHGSVCTSQTAIVKRLGLTPDVRMLQFAAFVFDLSIGEIIAPLITGACLCIPSDHTRMNGLTQYI
RDTGINWAFLTPSFIRTINPAEVPGLELVLLAGEAVPRDVLTTWFGKVRLVNGWGPAETCVFSTLHEWQSVNESP
LTVGRPVGGFCWVVDPEDPHRLAPTGTLGEVVIQGPTLLREYLSDPERTQASTVYDLPKWAPRPDSRHWNKFYK
SGDLCYYNQDGTIEFSTRKDTQIKI

>Trichoderma reesei_mod17_Aib

EAQVRKTPDLPAVWAWDGQLTYRQLNEAANRLAHYLINEHNVQVEDLVHVCFEKSVWHWVSVLAINKAGAV
WVPLDPSHPEQRLRQVASQTQSTLALTSDTTKSLLSHIIDRVVEVSPALFEQIDVRLGEKEPQVSVSASNAAYILFTS
GSTGTPKGLVMTHGALTTSQTAIKKRMGTGTHTRALQFASYVFDMSVGEGFVQLISGACIFIPSEHTRMNGLKQFI
TEHRINSLWLTPSFIRTLSPEQVPTVDFVFLAGEAIPRDVFTTWCTKVRLWNGWGPAETCVVSSLHEFTSLDESPLT
IGRPIGGYCWIVDPTDHTKLAPIGTMGEVVIQSPTILREYLADVERTKASTVYELPEWAPYRDQAPWSRFFKSGDL
ASYNPDGTLEFASRKDTQVKI

>Trichoderma reesei_mod18_Gln

SKHALSSPHHEAIYSSEGSLTYGELDHLTDILATHLSSLGAGPETVVPFCFEKSMWAVVAILAILKAGAAFVPLDPS
HPTSRREALVKEVSARVLVASSSAIASCKGMFEHVVELSPSVMAKLAASVTPRILPKVGPRNTAYVLFTSGSTGKP
KGVVMQHGSFSSTTIGYGKVYNLSPLSRVFQFSNYIFDGSLGEIFGPLAFGGTICIPSEDERLGSAPAFMSTSKVNTA
MLTPSFVRTFTPDQVPHLTTLVLGGEAASKSTLEMWVNRVTLYNGYGPAEACNYATTHVFKSSSESPRIIGSGFNG
ACWVVEPDNHNILAPIGCTGELVLQGHALARGYLNDKAKTEQSFVSDISSLPSSSLHEPKRFYLTGDLVRYNSNG
KLEYLGRKDSQVKL

>Trichoderma reesei_mod19_Gln

DNAQRDPNHEAIFSSEGSMTYAALDRLTDVLASHLCQLGVGPETIVPFCFEKSMWAVVAIVGILKAGGVFVPLDP
SHPINRREALVKEVGAQIIIASESAAASCAGMAPRIVQLSSDFMARLSAPSQTLVEPRRPSPSNAAYVLFTSGSTGKP
KGVVMEHSALTTSTIGYGRVYELSPASRVFQFSNYIFDGSLGEILATLTFGGTVCIPDEVERLQDAPGFVRKAQINT
AMLTPSFVRTFTPDQVPSLKTLVLGGEPAGRDILDAWCDCVKLINGYGPAEACNYATWHPFSSSRDSPRVIGKAF
NSSCWVVEPNNHHALTPVGCVGELAIQGHVLARGYINDLERTRSSFVTEIASLSSVIAGPQRFYLTGDLVRYCSDG
SLEYLGRKDTQVKL

>Trichoderma reesei_mod2_Ala

KQIEAQPDAPAFDSWDAKLTYREVGVLATRLAAKLQRLGVGSDVMVPISFPKSTPAMITMVAIQMAGGALVPLD
PAAPLARLQSIVSDTQAKLIVTHPSLEEKLQELGVDLLIVDLSMLDKLPDPSTKFISSDATPESLYAVLFTSGSTGKP
KGIRIPHSSLCSVSDAHAAETGVGPGSRVFQFSAYTFDIGILDVLVTLMRGGCVCVPSDHDRLNNLAGVISALQAN
WVFLTPTVADMLNPADVPCLTTINLGGEAVNKKSSERWQGYTTLNGLYGPAEASICARNADVHNGASTNLGFP
LSSAFWAVEPNDPSRLVPIGCIGELLIQSPILAYGYLNADAKNNANWLEDMTYDWLPANGPKRAYRTGDLVCRN
PDGTFEYMGRRDNQVKI

>Trichoderma reesei_mod20_Pheol

SKQVARRPAHQALYSSEGSMTYSKLDRLTTQLAVYLSSLGVRPETIVPFCFDKSIWAIVAMIGILKAGGVFMPLDPS
YPASRRQALIDEVNAQFMIVSPTTAPDSQGMVQNMIELSPSLIAFFSTIDTGDQSFIKSGPNNAAYVLFTSGSTGKP
KGVVIDHKAISAALLRQREAFSFNDDTRTLQFANFVFDACIAEIFSALVAGATVCVPTEHERVHNTAAFIREARIN
HAFLTPTFIKTLSPEQIPGMKTVILMGEAPSQEIIDTWADEIDLHNGYGPAEGCVGSTNNTYSSSIKVSVTNVGRSFT
HGLWIVDPDNHNRLMPIGCVGELLLQGSSLARGYINDEEKSRQSFIDQVEWLPANVNVGERRFYKTGDLVRYTP
DGSIEYVSRKDTQVKI

>Trichoderma reesei_mod3_Aib

EKQALERPGAPAVVGWDRVFTYAELNEAANRLAHHLTQTFAIKADELIHVCFEKSAWHFVAILAINKAGAGWV
PLDPSHPEQRLRQIASQTRARIILTSPANLDICARLGLSVIEISPFFDQKLIKSGMNSSAGPDVKVTPRNIAYVLFTSG
STGTPKGLVMEHGSVCTSQTAISRRLGLTADVRMLQFAAFVFDLSIGEIVAPLISGASLHIPDENTRLNDLPNFIQK
KQINWAFLTPAFARTLKPEDVPALDLLLLAGEAVSRDVFESWFGKVRLINGWGPAETCVFSTLHEWRSINESPLT

VGRPVGGFCWIVDPEHPDKLAPTGTVGEVVIQGPTLLREYLADAERTKLSTVYDLPAWAPRRELQHWSRFYKSG
DLCYYNPDGTIEFSTRKDTQIKI

>Trichoderma reesei_mod4_Ala

ERQTKQTPHAHAVHAWDGSLTYSELDLAANRLANLLIQRHGVKVGDVVHVCFEKSLWYVVSVLAINKAGAA
WVPMDPAHPFQRLQQVASQTGAKLALSSAIHSPLCSKLLDTVVEVSSNLDEQLKSDETISHVKPTTKVTPNDAVY
LLFTSGSTGVPKGIIMEHASLCTSQRDIAKRLGLTSSVRMLQFSSFVFDVSVGEIMLSLMHGGCICIPSDHDRLNNL
DGFIRDAEVTWAFLTPSFARTLRPQDVPSLELIVLAGEPVSQDVFDLWFGKARLVNGWGPAETCVLSAIHEWKSA
DESPLTIGRSVGSFAWIVDAENSNRLAPVGCIGEIVMQGPTLLREYLADPAKTASSTMTSLPNWAPRANDKKWG
RFYKTGDLGFYNPDGTIHYSGRKDTQVKI

>Trichoderma reesei_mod5_Aib

ARQAELRPDSVAISAWDAELTYSQLNLAANRLANHIITAYGIKPNDFIHVCFEKSAWHFVAILAINKAGAAWVP
LDSSHPEQRLRQVVSQTNARLVLTSPSNSTLCSGLLADVLEVTPALDQKLAATVGSQAPKVAVTPEHAVYALFTS
GSTGTPKGLVMQHRAVCTSQTAIAKRLGLSSDIRQLQFAAFVFDLSIGEIIAPLISGACVCVPSEDVRMNSITEYIRD
QRINWAFLTPSYVRTLRPKDVPGLELLLLAGEAVPKEILNTWFGKLRLVNGWGPAETCVFSTLHEWKSVDESPLT
VGKPVGGFCWVVDPENPHKLAPVGTLGEVVIQGPTLLREYLADPERTAASSATAPDWAPQPDSKHWGRLYKSG
DLCSYNPDGTLEFSSRKDTQIKI

>Trichoderma reesei_mod6_Aib

ERQAISTPDAIAIDSWDGKLTYAQLDEAATRLSHHLIKTYDVAPDDLVLLLFGKSLWYIISTIAVNKAGAAWVPLD
PAHPMQRLQQVTRQTKAQVILASSLQCELAQELLNTVVEVSQALDDALTTAGSTLRTPDAMVSSRDKAYVLFTS
GSTGVPKGIVISHGSVCTSQTAISSRLGLHSGVRMLQFSAFVFDVSVGEIYGSLIRGACVVIPSDEIRMNDLTRFMRE
KEVTWACFTPSFIETLHPADLENLELVILEGEPSKRHILEEWFGKVKLINGWGPAETCVFSSMHEWKSATESPVTIG
KPVGCFAWIADPDNHHRLAPIGTVGEIVLQGPTLLCEYLDDPMQTQASILKSIPSWAPRRES-
QHWNRFYLTGDLGCYNPDGTLAYHGRKDTQVKI

>Trichoderma reesei_mod7_Gly

AQQAARDPHHEAIYSSEGTVTYATLDRLSSLLAHHLHAHGVRPESVVPFCFDKSAWAIIAMLAILKAGGVFLPLD
PSHPRNRREALIEEVGAEVMIVSPSSSVTCEGLTPTMVELTTPLLEQLSSTYDAFQQIHPKPKPSNAAYVLFTSGSTG
KPKGVLMEHSGFATSTLGHGRVYNLGPTSRVFQFSNYVFDGSLGEIFTTLSFGGTVCVPSETERLQAPTFMRKSRV
NTAMLTPSFVRTFTPDQVPSLQLLVLGGEASSKDLIETWCDRLRLVNGYGPAEACNYATTHDFKPTDSPRTIGRGF
NSACWIVEPTDYNRLTPIGCVGELIIQGNALARGYINDPKRTADSFVTAVDCLPRDMISGPHRFYLTGDLVRYNST
GEMEYLGRKDTQVKL

>Trichoderma reesei_mod8_Aib

EQQAVVRPEAPAINGWDAKFTYAQLNEAANRLANHLVAEYEINNDELIHVCFEKSAWFFVAILAINKAGAAW
VPLDPSHPSQRHQQVVNQTKARLALVSTSHISTCVDLVDDILEVSSTTDELLRKSQSSRHGPTRKVSPSNAAYVLFT
SGSTGTPKGLVMEHGSVCTSQTAIVKRLNMTPSVRILQFAAFVFDLSIGEIVAPLITGACICVPSEHARMNALPEFV
RQNNVNWAYLTPSYIQTLSPKDVPGLELVLLAGEAVSRDILDAWFGKVRLVNGWGPAETCVFSTLHEWRSKDEE
SPLTIGRPVGGFTWIVDPENPQKLAPIGVPGEVVIQGPTILREYLDDPVRTSDSTVYSLPPWAPNRGTKWNRFYKS
GDLCSYNADGTIEFISRKDTQIKI

>Trichoderma reesei_mod9_Lxx

EVALVRGDAPALSGWDKSFTYKEMTEATNRLAHYLVNDYGVKVGDIIHVCFEKSAWFIIATLAINKAGAAWSTL
DTSHPIERYQKIVSQTGSKLALASAANSYRCVGVLPHVIELTPELDARLAKNTSWSACGPAVAVIPSDMAYILFTS
GSTGVPKGVVIEHATLCTSQTSLSQTLGFHEEYKVLQFSSYSFDAAVFEIDSTLLTGACLYVPSWDEQMNELVGYIR
KHTITCTLLTPTLARTLRPEDVPSLNMLILGGEAPTRDILDIWFGKLKLVNGWGPTEACVIACLHSWTSVDESPSVI
GRPIGGSIWVVDPDDATRMAPVGTVGEIAVQGRNLFRGYLSDPVKTAAATVTGLPEWVP-
KRDSSAHWDRFYLTGDLGFINEAGNVEYCTRKDTQVKI

>Trichoderma virens_Aib/Ala_mod5

TYAQLDRMANRLAHHLVKAFDVKTGDLILLCFEKSLWYIVSIIAINKAGAAWVPLDPSHPMQRLQQVVNQAKA
KLFLTSPLYHTLGDELSDTVLEISQALDDILLKVEPSTLAPDVALSPRNAAYVLFTSGSTGTPKGFVIEHKAVCTSQT
AITNRLGLHRGVRMLQFASYVFDLSVGEICCTLICGACLVIPSDEVRLNNLAKFMREKDINWAWFTPSFIRTLEPA
DVPKLELVLLCGEASDKKILEIWVGKVQLFNGWGPAETCVFSSLHEWKSVTESPLTVGKPVASFIWIVDPKNPHR
LAPVGTVGEAVVQGPTLLREYLDDPIRTKASILESSPRWAPRCESQHWNRFYLTGDFCCYNPDGTIEYHGRKDTQI
KI

>Trichoderma virens_Aib/Isovaline_mod7

TYGQLDQAANRLANHLIADYEIKNDELIHVCFEKSSWFVVAILAINKAGGAWVPLDPSHPTQRHQQIVDQTKAR
LALVSPSNISTCIDLVEHVVEVSPVTDEILSKTESSHRGPDREISPSNAAYVLFTSGSTGTPKGLVMEHRSVCTSLTAI
TKRLKIRPSARTLQFAAHVFDCAVGEIISSLFTGGCLFVPSDHDRMNALPEFIRQNKINYMWSTPSFIRTLSPMDVP
SLELVVLVGEAVTRDIMDTWFGKVRLINGWGPAETCVVSTIHEWQSIDESPLTIGRSVGGFCWIVDPEDPQKIAPV
GALGEIVIQGPTILREYLADPVRTSGSTVYNLPNWAPNRRDKYWNRFYKSGDLGLYNADGTIQFASRKDTQIKI

>Trichoderma virens_Aib_mod1

TYLELDRAANRLAHHLVKSCGVRDQDLVHVCFEKSAWFFVSIIAVNKAGATWIPLDPSHPIQRQQQVVSQTKAA
LALASISNVDMCSKLIGNVVEVSHTLDEALAQTEMSSHGPVSNVSPRNAAYVLSTSGSTGTPKGLVMQHQAVCT
SQTAITKRLRMTSDVKMLQFASFVFDLSIGEIFGPWVVGGCICVPSEETRMNDLVNFINTMKINWAYLTPSFARTL
NPVDVPGLELLLFAGEAVRRDVFEAWFGRLRLINGWGPAETCVFSTLHEWKSLDESPLTIGRPVGGHCWIVDPQ
DPQRLAPIGTFGEVVIQGPTILREYLADFTKTESSMVRSLPEWVPNRTSTHWDRFYKSGDLCRYNADGTMEFGSRK
DGQVKI

>Trichoderma virens_Aib_mod15

TYSEFNRAANRLANYLTANYSIKTDDLIHVCFEKSAWFFVSILAINKSGAAWVPLDPTHPEQRLRQVVSQTCSEIT
LTSAANKNLVSSLSKSVIIVDSELDLQLSKVEEYSQKGPVTATSSDNAVYVLFTSGSTGTPKGLVMQHGSVCTSQT
AIVKRLGLTPSVRMLQFAAFVFDLSIGEIIAPLITGACLCIPSEHTRMNSLTQFIRDMEINWAFLTPSFIRTINPVEVP
GLDLVLLAGEAVPRDVLTTWFGKVRLINGWGPAETCVFSTLHEWKSVNESPLTIGKPVGGFCWVVNPEDPHRLA
PIGTLGEVVIQGPTLLSEYLSDPERTRSSTLYNLPKWAPRPDSKHWNKFYKSGDLCYYNQDGTIEFATRKDTQIKI

>Trichoderma virens_Aib_mod16

TYGQLNEAANRLAHYLISEYDVQVEDLIHVCFEKSVWYWVAIFAINKAGAVWVPLDPSHPEQRLRQVTSQTRSK
LALTSNNTRNLLSGIIDRVIEVSSALYRHINVSLGAKNPQVSVSSHNAAYVLFTSGSTGVPKGLVMTHGGISTSQM
AIKKRMGINSSNRTLQFASHVFDLCLGESILQLISGACIFIPSEYTRMNGLKDFITEHKINTLFLTPSFVRTLSPDQLPS
VTLLLLAGEAVPRDILTTWFGKVRLWNGWGPAETCLFSSLHQFQSVDESPLTIGRPVGGFCWVVDPTNANKLAPI
GTLGEVVIQGPTVLREYLADVERTKATTMYKLPAWAPYQDQPSWSRFFKSGDLASYNPDGTLEFSNRKDTQVKI

>Trichoderma virens_Aib_mod9

TYKQLNEASNRLAHLLVNKYNVKPDDLIPVFFEKNAWYFVAITAINKAGAAWVPLDPSHPVLRLRQILSQTGTT
LALSSSANAVLCSTLVRKVVEVNAELDNKLLATESSAHGPVVDVSSRNAAYVLFTSGSTGIPKGLIMEHGSVCTSQ
VAIAKRLGLNSKVRILQFAAFVFDLSIGEIVGPLISGACICVPSEHIRMNSIANFINRQGITWTYLTPSFVRTIKPSEVP
NLKLLLLAGEAVPRDIFATWFGKLRLINGWGPAETCCFSTLHEWQSADESPLTVGRPVGGFCWIVDPENPHRLA
PTGALGEVIIQGPTILREYLSDVDRTEAAVIKSLPDWAPFREQSSWSRFYKSGDLCVYNPDGTIEFSSRKDTQVKI

>Trichoderma virens_Ala_mod3

TYSELDRAANKLANFLIKGHGVKVGDIVHVCFEKSLWYVVSVLAVNKAGAAWVPMDPAHPLQRLQQVVSQTG
ARLALASSVHRHLCSKLLNTVVEVSLTLDEQLNRDEGISDIKPSVAVVSNDVAYVLFTSGSTGVPKGIVMEHGSLC
TSQRDIAKRLGLKSNVRMLQFSSFVFDVSVGEIMLSLMHGACVCIPSDHDRLNNLGGFIRDAEVTWAFLTPSFART
LCPQDVPNLELIVLAGEPVSQDVFDLWFGKTRLVNGWGPAETCVLSAIHEWKSADESPLTIGRSVGSFAWIIDVE
NPQRLAPVGCIGEIVMQGPTLLREYLADPAKTVSSTLTTLPNWAPRANDRKWGRFYKTGDLGFYNPDGTIHYSG
RKDTQVKI

>Trichoderma virens_Alanine_mod8

TYREMAETTNRIAHHLVTDYGVKVGDIIHVCFEKSAWFIIATLAINKAGAAWSTLDPSHPTERYQKIINQTGSQIA
LSSPVNSNRCASLLPNVIELTSELDSKLAQNVEWSTSRPAVNVTPRDAAYVLFTSGSTGVPKGVVIEHGSLCTSQIS
LSKALDFNEEFRVLQFSSYSFDIILFEIGSTFLTGACLFVPSWDEQMNELVEYIRKHQLTFMVLTPTLARTIRPEDVPS
VDMLVVAGEAPTRDILDIWFGKLRLANGWGPTECSVIACLHQWTSVDESPKVIGRPIGGSCWIVDPEDATCMAP
LGTVGEIVIQGRNLLREYLSDPVKTATATITGLPQWVPKRDSIHWDRFYLTGDLGFINEAGNLEYCTRKDTQVKI

>Trichoderma virens_glutamine_mod17

TYGELDHLTDILATHLSSLNVGPETVVPFCFEKSMWAVVAILAILKAGAAFVPLDPLHPISRREALVREVNARVLI
ASSDAVASCAGMAEHVVELSPSVMARLATSVTLKILPKVGPRNTAYILFTSGSTGKPKGVVMQHGSFSSTTIGYGK
VYNLSPLSRIFQFSNYIFDGSLGEIFGPLAFGGTICIPSDDERLQCAPDFMHRAKVNTAMLTPSFVRTFTPDKVPHLK
TLVLGGEAASKSTLEMWVDRVTLFNGYGPAEACNYATTHIFKSSAESPRLIGSSFNGACWVVEPSNHNKLTPIGC
TGELVLQGHALARGYLNDKMKTEESFVCEIGSLPSSLLHEPKRFYLTGDLVRYNSNGELEYLGRKDSQVKL

>Trichoderma virens_Glutamine_mod6

TYANLDRLSNLLAHYLHRLGVRPESIVPFCFDKSPWAIVAMLAILKAGGAFLPLDPLHPRNRREALVQEVGAEIM
IVSPSSSVPCEGLTSIMVEFTIELLEQLSSRYDAFQEILPKAEPSNAAYVLFTSGSTGKPKGVLMEHSAFATSTLGHGG
IYNLSPASRVFQFSNYIFDGSLGEIFTTLSFGGTVCVPSEDERLQKAPSFMREARVNTAMLTPSFVRTFAPEQVPSLR
LLVLGGEPSSKDLLETWCGRLRLVNGYGPAEACNYATTHDFKPTDSPHTIGRGFNSACWIVDPTDYNKLTPIGCI
GELIIQGNALARGYINDADRTKNSFITNVDCLPKSIISGPHRFYLTGDLVRYTPDGQLEYLGRKDTQVKL

>Trichoderma virens_Gly_mod2

TYTELVEAANRLANYLTKNFTIKTDELIPVCFEKSSWYIVAILAINKVGAGWVPLDPSHPEQRLRQVVSQIRAKIVL
CSPSNAYICNGFGLTVIEISSVFNQKLVESGVTSTTGPATKVTPRNIAYVLFTSGSTGTPKGLVMEHVSVCTSQTAIS
QRLELTTDVRMLQFSAFVFDISVGEIVATLISGASLYIPDENTRLNNLPNFIQNNNINCAMLTSSFLRTIKPADVPAL
ELLVFVGEAPARDVFETWFGKVRLINGWGPAETCVLSTIHEYQSIDENCLTIGRPVGGFCWIVDSDNPQRLAPTG
AIGELVVQGPTLLREYLADSEKTKLAIVYDLPDWAPRRESQYWNRFYKSGDLCYYNPDGAIEYLTRKDTQVKI

>Trichoderma virens_Leucina_mod14

TYTELDRSANRLAHYLIQSSGVQVGDCVHVCFEKSAWYVISILAINKAGAAWIPLDPSHPTERHQQIVSQTGSTLS
LTSPANAAKCASVVASVLEVTGEFMETLEEQYKDSKPATSVSPKDVAYILFTSGSTGIPKGVVIEHGALCSSQTSYV
RRLGHAPGVRMLQFSSFVFDASIAEIFAPLISGACVCIPSWETQMNSLTGYICKENVTWALLTPSFARTMDPLEVA
CLEILILGGEAVSRDVFELWFGKVRLFNAWGPTESCVFGSLHEWESISESQMTIGRPLGGYCWIVDPEDPQKLAPT
GTFGEVVIQGPNLLREYLADKDKTASSIVAALPDWAPNRRSHHWNRFYKTGDLAKYNPDGTLQYYSRKDTQVKI

>Trichoderma virens_Leucine_mod11

TYAELNSAANKLAHYLIKSYSVKLNDLVHVCFDKSAWYIVSIIAINKAGATWVPLDPFHPEQRLRSIVNQTKSTLA
LASPGNITLCSALVDNVVEVSSALDSMLPAQDGLESPKVSVSSRTAAYILFTSGSTGTPKGVVIQHRSLCTNMNAI
AKRVRYHLDVRILQFSAYVFDFSILEIIMSLLQGACVCVPSEHIRMNGIVDFIRDMNINWLYLTPSFLRTINPIDVPN
VELVFAGGEAIPRDVFETWVGRVRFINGWGPTETTVVGSIHEFESVDESPSTIGHPVGGFCWIVDPNNPQLLAPTG
TLGEIVIQGPTLLHEYLDNPDKTQEAILYDLPEWAPRPDENNWGRFYKTGDLGFYNANGKIEFSSRKDTQVKI

>Trichoderma virens_mod1_Aib/Vxx

DLQRAIEYNNEEPDIVSSCLHQVIEDHAAKRPDAMAICAWDMSLTYNQLNLIANRLSCYLVDNYAVQSGDFIHV
CFEKSAWFYISVLAINKAGAAWIPLDPSHPIQRQKQVVMQTKAKLALASPSNATLCADLVDNVVEVSAALEEILR
ERRPGSNSLRPVSPSAACYALFTSGSTGTPKGFVIQHRALCTSQTALVKRLHMTSDIRMLQFASYVFDMSIGEVVA
PWIAGACICVPSEQVRMNGLAEYIRTMEINWVFLTPSFSRTLNPDDIPGVEVMILAGEAVGRDILETWFGKVRLIN
GWGPAETCVLSTLHEFESLNDSPLTIGRPVGGFCWLVDPENPRHLAPIGTVGEVVIQGPTLLREYLADPAKTESTT
VKPLPDWAPNSDSEHWSRFYKSGDLCKYNSEGTIAFVTRKDTQIKIRGLRVELGEIEHQIKACLNGVCQVVVDKI
NSGAGSNLVAFFCFSTETRMVATASTNADGNTSMFLPVTTQLKSQILGMIGHLSVNLPRYMVPSL

>Trichoderma virens_mod10_Lxx/Vxx

LELAIASNSDTPEVISSCIHDLVDKQARITPDAPAISAWDAELTYSQFIAAANRLAYHLIHSHGIKPNDLVHVCFEK
SAWHFVSIIAINKVGAAWVPLDPSQPEERLLQIVRQTKARAALVSPNNASLCTKLVENVIEVTAELDGQLGLSVS
NSYVPRVAVPPHSVAYIIFTSGSTGTPKGVVMTHLSLCTSQTALADRLGMTPSVRILQFSAFVFDMFIGETMASLIY
GACVCVPSDRERMENVVGFINEQRINWVFLTPSFARTIQPKDVPGLQLLLVGGESIPQDLVHKWFGKLRLICAWG
PTETCVISATHEWTSAEESSLVIGRPVGGFCWIVDPDMPHRLAPIGTVGEIVVQGPTLLHEYLNDSQKTEAALMST
PTWAPRPDSQHWHRCYRSGDLCFYNHDGNIVFSGRKDTQVKVRGFRVELGDIEHHIRDRLDAPCEVAVEVLKTT
VGANLIAFVTFNVEADAANGSSSADIFLHLDSGMQETFSALLEQLKVLLPPYMVPTTFVPCQY

>Trichoderma virens_mod11_Lxx/Vxx

FAREANGEDAPEILNTCLPRLIEIQAEIRPEAIAVQGWDKQFTYNELNRAANRLAHHLVSDIGVKPHDIVQVCFS
KSAWYVVAILAINKAGAAWGPIDPSHPLHRHQQIISQTKAQVALTSLDNVERCSLLVPSVVTVSSSLDSDLSEKNY
DSNHGPDVTVTSHYAAYVLFTSGSTGMPKGLIIEHGALCTSQTAVGKRFGFTPKVKILQFTTYVFDFSISEIIAAMLF
GACVCIPSEETRMNRLKGFIQESGINWLFLTPSFLQTLKPDDVPGVELVAVGGEPLPRALFEEWVGKVRFFNCWG
PSETCVMSAMHECKSEEESNMTIGRPVGGFCWIVDPEDPQRLAPTGTVGELIVQGPTLLREYLGDPEKSKGSILCS
RPDWALYPDSENWSRFYKSGDLCSYNPDGTIKFSSRKDTQVKVRGLRVELGEVEYRIKEALHGALQVAVDVFKSG
ATSTLAAYFCFSDATAVVSGNDPDNIFIPLTEEVQQLIATMVGEIKIMLPEYMIPALFIQCQ

>Trichoderma virens_mod12_Aib

IEQSHKFNADIPEVINSCFHDLVEYQATHRPSAMAIRSWDGEYTYAELNQAANRLANHLVQTYDIKTNELIHVCF
EKSVWYFISILAINKVGAAWVPLDPSHPIQRLQQVVNQTRAKIALCSPDNVDLCYGLVESVVQVATELDSQLLQL
VTSQQGPACNVSPNNIAYVLFTSGSTGTPKGLVMQHRSVCTSQIAIAKRLRLTPEVRILQFAAFVFDLSIGEIIATLIT
GACLCVPSEHARLNELTKFIQAMDVNWAYLTPSFVRTLNPDDMPNLELLLLAGEAVPRDVLTAWFGKVRLING
WGPAETCVFSTLHEWKSIDESSLTIGRPVGGFCWIVDVEDSGRLAPFGTIGEIVIQGPTLLQEYLADPERTNASIRPS
TEWCPRPDGTNWNRFYKSGDLCRYNTDGTIEFSTRKDTQIKIRGLRVELSEVEYHIQQALTGVRQVAVDLFNGDN
GSALVAYICFSDETRTPGIEVDNSNTPFSSPDESLRNQLIALVGELGVALPRYMIPTLFIPC

>Trichoderma virens_mod13_Pro

LLSPWDLQHAVESARLKPSSETCTHWLIQETVQSRPDDIAISSWDAELTYSQVGTLASRLAVKLQARGVGPESLILL
CFPKSAWAVIAMVAVEMAGGAFVPLDPSAPAARLKGIIDDTHATLAIASPSSSKVLEGLGVEIMSVDQEVLSGLP
DPVSKVVSNVQPRNASVILFTSGSTGKPKGMVIQHNNLCSSSDAYGNDLNIGPGTRVYQFSAYTFDVGVLDCLVS
LMRGACLCIPSDHDRMNNLSGSMRSLKANWVFLTPTVADLLSPTDVPDLKIVCLGGEAISKKCADRWVNHVEL
HGLYGPAEASICAWNPLVGKSGRSTNLGRPISSAFWVVEPSNVRQLVPVGCIGELLIEGPMLARGYLNVSAEVAA
NWIEDVDWLPGGRKRVYRTGDLVRRNADGTFDYMGRKDTQVKLHGQRVELGEIESQIHEFLPRNMAAIVDIVT
ADDENATNILMAFLWYTEDNDAPSQTLQLMQSVSDQAQSIISHLDSSLAESLPSYMIPSSYLIFEGKPE

>Trichoderma virens_mod14_Vxol

DIEQVIKRNNYSIEPIESCVHELISIQAAKNPQQEALYSSSGSMTYREIEQLSNQLASHLIQLGVKSETIVPFCYEKST
WSIIAMLGIMKAGGVFLPLDPAHPLNRRQALVDETSAQFMIVSPTTAKECEGMTKNNIELSPTFFASANSSVTTRK
FKPIKPKPGSAMYILFTSGSTGKPKGVIIEHKGIASFLFRSCEILNMGGDSRMLQFSSYVFDASIFEIFAVLISGGTVCV
PTETERMQHTANFIEKGRINSAILTPTFVKTLIPELVPSLEILLLCGEAPPKEIVDLWRQHVSLWNAYGPTETCVMS
TLHHYTDIATPATTIGRGFAHHCWVVNPENHHELTPIGCVGELLVQGESLSRGYINDEEKTNNAFITHVKWLPD
NIDIGKRRFYKTGDLVRYNHDGSLDYLGRKDTQVKIRGQRIELGEIEYQIKLQSSEIKHAVVDIICDDIHQSLVAFVS
LFGDKGPSEATPVQLMSHNNELQELFSQLAANLTTVLPSYMVPKYFIAVESMP

>Trichoderma virens_mod2_Gln

QQSMYWNNHEIVVEEHCMHDIISTHAKSCPNDEALFATEGSLTYAALENLSDHVAYQLLQYNVQPETVVPFCM
EKSIWAVVAMLGILKAGGAFMPLDPSHPESRRLALVQEVNAKVILTSPSMATSCEGMAEHTIEVSASLLTVAPNIK
SDYHTLSNYKKPEPHNAAYVLYTSGSTGKPKGLIMPHSAACTSLLRHPEKFSIDKSSRSFQFASYVFDVCILEIFVSL
FVGATVCVPTESERIGNTWRFMTEARVTWTCLTPSFIRTLDPDTVPTLRTLCMGGEAPTKDILTKWHGKVELINA
YGPAEACVDVAGHVFKSQDESPTTIGRPFAHKLWIVEPYNVNRFTPIGCIGELVIEGHAIARGYINNEEKTNESFL
NDLEWLPNVPTGDKHRVYKTGDLARFNSDGTIEFFGRQDTQVKIRGQRLELGEIEYNIKGNLSDVQHVVADLIER
ETGKMIIAFVTFENHLCDTMADAADPYGNFIRDGKLNSSFQDLMLGLRRVLPGYMIPSIIFPLR

>Trichoderma virens_mod3_Vx/Lx

IEFAHASNPNKPLVIDSCVHTLIEERTKINPDSPAVCSWDADFTYSELDENANRLANYLSSMLGVKSGDLVMVCF
DKSAWYIVAILAINKAGAAWVPIDPSHPLERHQSIVSQTGATLALCNSSNLSKCTQLFTDVLEVNTQFISELRGIKA
YEGKPNAKVSPEDVAYLIFTSGSTGVPKGVVIQHRAICSSQVALSQRLGMHDGVRMLQFSSFVFDASLFEIMSPLIS
GACVCIPSWDVQMNSLPAFIRDVNVTWAFLTPSLASIIKPQDVPSLELLAIGGEAPSKEVFSIWLGNLRLFNVWGP
TETTVIAACHEVKSTQLSHLTIGQPICGNCWIVDPKDPTKLAPVGTVGEIVVQGPNLLQEYLADPEKTATATLTSL
PEWAPNRDLWNRFYRSGDLAVYNSDGTIRYCSRKDGQVKIRGLRIELGEIEHHIRKNMDATSQVAVEAFRSESGT
NLVAFICQNSDTIPAAMTGNMTSEDIILPLTDQLKSSLETLRNSLISSLPGYMVPTFFVPC

>Trichoderma virens_mod4_Aib

QKSATFNSQVPEAIESCFHQLFEKQAIETPDALAICSWDGTLTYAELDKAANRLAHHLMSQHAVKLEELIHVCFN
KSVWFFVSILAINKAGAAWVPLDPSHPPERLRQIVKQTKAQVALASESHVALCSDLMPHVIEVSAFLDQKLSSND
GYSQNSPATKVSPNNAVYVLFTSGSTGIPKGLIMEHRSVCSSQKAVIKRLGLHSNVRMLQFAAFVFDLSIGEIVAPL
ISGACICVPSEWARMNDIAGFIREKEVNWAYLTPSFIRVLKPEDVPSLELLLLCGEVTPRDVFNTWVGKLRFISGW
GPAETCVFSTLHEWKSDTESPLTVGNPVGAFCWIVDPEHPDRLAPIGTVGEVMLQGPTLLREYLDDPERTKSSIVM
PLPDWALERGSQWPRFYKSGDLAMYNPDGTIEFCSRRDTQVKIRGLRVELSEVEHSIRESLEGVRQVAVDISTSSSG
SNLVSYLCFSEETRSSSNSKISLDDIFMPLTADMQPLLAAMIGQLKVTLPSYMIPTLFIIC

>Trichoderma virens_mod5_Pro

LVGGWDMKHAVNSQMLKPSTESCTHWAIQDTVRTYPDSLAVASWDGELTYAELGSVVSRLAAKLQLLGIGSET
LVPLCFPKSICAVITMIAVQMAGSAFVPLDPKAPKTRLSSILDDIKANIALTSPCCESNMEGLGVPLIVIDKDFLDAL
PNATASQRSGNPSDAAVILFTSGSTGRPKGMVIQHNALCSSANAYGRDLQIGPGTRVFQFSAYTFDVGILDTLVTL
MRGGCLCIPSDHDRLNDLPGAINKAQANWVFLTPTVADLLNPADVPTLRVVCLGGEAINQKTADRWKDCVEL
HGLYGPAEASICAWNSTVGKSGKSTNLGRPLASAFWVVEPNDIQKLVPVGCIGELLIQGPMLARGYLNVRTEQA
ANWVEKFSANWLPHDFPSRAYRTGDLVRRNADGTYDYISRKDTQVKLHGQRVELGEIESQIHKAIPAEMSAIVD
VVKSHEGQHQDSLLAFLWYTDNSAPSQQLPLELIGTLSDEQRVLISDIDMALSINLPSYMIPSSYLIFN

>Trichoderma virens_mod6_Ala

LERAIAINQELPEFIDSCLHTLIENQANQRPDAIAISASDGELTYGQLNSAANRLAHHLIERYSVQIEDRINVCFEKS
IWHVISIIAINKIGAAWVPLDPSHPEQRLRQIVEQTGAKLALTSSNQFALCAKVIKDVVEVSDTIYDQLVNAAVSS
NNPGVGVSPHNAAYVLFTSGSTGTPKGIVLEHAAVCTGLNAIAKRLQLPASARMLQFSAFTFDVSVGEIFTSLITG
ACVCIPSDWARMNNIAAFIQEKKINWAFLVPSYIRTIDPEDVPSLEVIALMGEPATKDIFNTWFGKVRLFNSWGPT
ETCVISSSHEWKSANESPHVIGKPLGAHCWILDPQNPSQLAATGIVGEVVVQGPTLLREYLGAPDKTKASIIDALP
DWVPHQALPGWGRLYKTGDLCFYNSDGNIEYSSRKDTQIKIRGHRVEAGEIEHRVLQAMAVDQVAVELVNTES
GNTLVAYLCFNLDCKAISLTATPDEVFLPLTDDMQQSISTAVAQLKTDLAHYMVPTMFIVCK

>Trichoderma virens_mod7_Vxx/Lxx

YDFAVAANGAPPTTIERCVHSLIESEANKHPTSMAVNGWDADFTYQQLDLCANRLAHHLINTFNVKIGDIVHM
CFEKSAWYIVAILAVNKTGAAWSPLDSSHPVQRYSQIIAQTGASLILTSPANASKCIPLVPHILEVSATLDATLVAD
RTNHRSPSINVSSADRAYIIFTSGTTGTPKGVVIEHGSLCTSQMALAQRVGLTHSARVLQFASHVFDASVFEIFAPL
LVGGCVCVPSWDEQMNDLTGYMINASVTAAFATPTVSRSLRPEKMPRMEILIVGGEAGASDILETWIGKVRLFN
GWGPTETCVIASVHEWTDIGDSARTIGRPITGNWWIVDPSNPQRRVPIGCIGEIVYQGPTLFREYLANPSKTEEVLI
SSLPSWATGGDWKSWDRFYRTGDLGFYNPDGTVEYCGRKDTQVKIRGLRIELHEIEYQIYTHLPAIRQVTVDVLK
AEEASHLVAYYCLNNDTQPSHLSVDSEGKGLFLPLLDDMVPQINALISNLNDILPPYMIPKIFI

>Trichoderma virens_mod8_Aib

VKQAIKFNSEAPEIIDTCVHTLIEAQARRNPDALAISAWDGTLTYRQLNECGNRLAHYLIRQYSVKPDDLIHVCFE
KSVWHFIAILAINKAGAAWVPLDPSYPEQRLRQVISQTRSTLVLASSRNAKLCSSLVENILQLDAELDKSLTLTENG
QSGPTVAVTHHNAAYVLFTSGSTGKPKGLVMEHGAACTSQTAIAKRLNLTSDVRMLQFAAFVFDLSIGEIIGPLIS
GACLCIPSEDTRMNGLKEYVNDTGVNWAYLTPAFVRTLNPEDMPGLNLLLLAGEAVPRDVLTTWFGKVRLVNG
WGPAETCCFSTLHEWASVDESPLVVGRPVGGFCWIVNPEDPQQLTPTGTLGEIVIQGPTILREYLADIERTKDSTVY
SLPTWAPQPDSEHWNRFYKSGDLGYYNPDGTIVFSSRKDTQIKIRGLRVELGEVEHYIRETLDGVRQAVVDVYQR
DGRSNLVAYVCFTDEMKNPSADEEFTANGLFASITDDLRKRIAIMLGELRVTLPSYMIPTLFI

>Trichoderma virens_mod9_Pro

SWDLQQAIDASRLRPATDSCTHWLIQDKIREQPEEIAITSWDGDLTYAQLGAHATRLAAKLQDLNIGPESLVPICF
PKSKWAVVSMVAIQMAGGAFIPLDPGASPLRLQGILQDTGATLAITSPSCQATLQSLGMETIAVDDEMISKLPSSF
VLQCEVQPVNSSVILFTSGSTGKPKGMIIQHNSICSSSDAYGADLNIGLGTRVFQFSAYTFDVGVLDCLVSLMRGA
CICIPSDHDRLNNLAAAITASQANWIFLTPTVADLLQPVDVPTLKTVCLGGEAISKKCAERWVNYVSLHGLYGPA
EASICAWNSMVGKSGRSTNLGHPISSAFWVVEANDPKSLVPIGCIGELLIEGPMLARGYLNVTDEVAANWMERV
DWLPGDNQLKRIYRTGDLVRRNADGTFDYIGRKDTQVKLHGQRVELGEIEACIHEFLPPNMAAIVDLVKDKDVP
DSLLAFLWYSEGESTSPARLMDTISDEARATILHLDTSLSTILPSYMIPSSYLLFNGKPEQT

>Trichoderma virens_Prolina_mod13

TYKELGVLASRLAWKLQGLGVGPESLIPLCFPKSTWAVVAMVAIEMAGGAFVPLDPNAPVARLRGIIEDTKSSLA
VASPSCQDTMRDIGIEVFAVDEALLLELSDPADGVKSMAQPDNASVVLFTSGSTGKPKGMVIQHNSLCSSGNAY
GCDLEIGPGTRIFQFSAYTFDVGVLDCLVSLMRGATICIPSDHARLNDLAGAMNVTKANWVFLTPTVADLLSPAD
VPYLKTLCLGGEAISKKCADRWVNCTNLHGLYGPAEASICAWNPAVGQSGRSTNIGRPTSSAFWVVEPNNYKRL
VPVGCIGELLIEGPMLARGYLNATADVASNWMENVDWLPGNNNKRVYRTGDLVRRNADGTFEFMGRKDTQV
KL

>Trichoderma virens_serine_mod10

TYSELDRTANRLAHLLVHDYAVKVGDIVHVCFEKSKWYFVAILAVNKAGAAWAPFDPAHPPQRLKAVASQTG
AKLALASTANTRLCEQVVDCVVEVSSTLDKNLWTTYDNIEKGPDINVTPMDAAYILFTSGSTGVPKGIVMQHGA
LCTNQAALSGWLGFDHTVRMLQFSSFVFDVSVGEIVQALMNGACVCVPSEHMRLNSLDSFVRDFNVTWAYLTP

SFTRTLKPKDFPSLKLLLLAGEPTTQDVLDTWFGLPNTRFINAWGPAETCVYNTLYEWQSNTESPLKLGRAVGAYI
WVVDVENPQRLAPTGCLGEIIVQGPPLLKEYLADPEKTAAATVTELPEWAPRRQSTTWNRFYRTGDLGFYDHDG
MLHFASRKDTQVKI

>Trichoderma virens_Val/Leu_mod4

TYSQLNLAANRLANHIIKSYGIKPGDLVHVCFEKSIWHFVATLAINKAGAAWSPLDPAHPEQRLRKIVVQTGAN
LVLTSPSNSMLCSALSEKVLEVTSSLDQKLAETVDSEAPDVAVTPDNVAYVLFTSGSTGTPKGVVIEHRSVCTSQTA
MIKRLRVTSDVRMLQFASFVFDMSVGEVIPTLFSGGCVFVPSEDIRMGNIAQYIRDQRINWAFFTPSFLRTLAPKDI
PNLEVLLIGGEAIPKEILNTWFGRVRLINGWGPVETCVCSSWHEWKSVDESPLTIGRSAGGFCWIVDPEDPHKLAP
IGTVGEVVVQGPTLLREYLGDPERTAASSVKAPKWAPQPDSQHWGRFYKSGDLCSYNSDGTLKFFSRKDTQVKI

>Trichoderma virens_valinol_mod18

TYSELDHLTTQFAVYLSRLGVRPETIVPFCFEKSMWAIVAMLGILKAGGVFIPLDPSHPTSRRQALVDEVCAQFMV
VSPTTASDCQGMVQNTIELSQSFIAHISTINVTKQSFVRPGPNNAAYVLFTSGSTGKPKGVIIDHKGVATVLLRQRE
DFSINGDSRMLQFSNYVFDACIIEIFTTLIAGATVCVPTEHQRVHNTAAFIREARINHAILTPTFTKTLSPEQIPGMK
AIILVGEAPTKEIIDIWVDKVELHNGYGPAEACVTSTNYTYSSSVNFSATNIGRSFTHGLWVVDPDDHNRLMPTG
CVGELLLQGSSLARGYINDEEKTRKFFIKEVEWLPSEANVGERRFYKTGDLVRYNLDGYVEYLGRKDTQVKV

>Trichoderma endophyticum-mod6

LKKAIAVNHEIPEFMDTCLHTLIEIQAEQRPNAIAISASDGEFTYSQLNEAANRLAHHLIKTYSVGVEDLIHVCFEK
SIWHIISVIAINKVGAAWVPLDPSHPEQRLQQIVDQTGSNLALASTTHSALCAKVINNILEVNSILDNQLVNAGVS
PGNPAVPVSPRNTVYVLFTSGSTGTPKGIVLEHAAVCTGLNAISKRLGLPASARMLQFSAFTFDVSVGEIFTSLLSG
ACVCIPSDWARMNNITGFIQENHVNWAYLVPSYIRTIDPEEVPSLEVVALMGEPATKDVYNAWFGKVRLFNSWG
PTETVVISSSHEWKSANESPSVIGKPLGAYCWILDTRNPTQLAATGIVGELVVQGPTLLREYLAAPDKTKASIISDLP
NWIPRQALPGCDRLYKTGDLCFYNPDGNIEYFSRQDTQIKIRGHRVEAGEIEYQVSQAMTVDQVAVELVNTESGS
TLVAYLCFNLDCKPISPTATPDEIFIPPTDDMQQLISTAVAQLKTTLAHYMVPTTFIVCK

>Trichoderma _lentifirme_(18me)-mod7

VEYAIRHNNEVPELVESCLHTLVEQQKMIRPESPAVHSWDGNLTYSQLDQAANRLANHLMAEYEIKNDELIHVC
FEKSSWFVVAILAINKAGGAWVPLDPSHPAQRHQQVVSQTRARLALVSPSNISTCVDLVEHVVEVSSTTDEILNK
TELSDRGPDREISPSNAAYVLFTSGSTGTPKGLIMEHRSLCTAQTAAVKRLRLPSGVRTLQFASYVFDAAVGEIATT
LITGGCLFIPSDHDRINALPEFIRQNSINWAWITPSLIRTLKPEDLPSLRTVILVGEAVTRDIMDAWFGKVRLLNGW
GPAETCVVSTLHEWKSINESPLTIGSSVGGFCWIVDPENPHKIAPIGAVGEIVIQGPTILREYLDDPVRTSASTVKNL
PVWAPNRETKYWNRFYKSGDLAFYNEDGTIQFVSRKDTQIKIRGLRVELGEVEHHVKIAFSGVQQIAVDVIKSGIG
ASLVAYFCFTDEIRITGTRAGSDDSGPFVQMDDELQGTLTTVIGQLNISLPRYMVPTFFI

>Trichoderma _lentiforme_(18me)-mod1

LEQSLKANMETPEIVDLCLHQLIERQAKDRPNTTAISAWDLELTYLQLDCAANRLAHYLVKSCGVKDQDLVHV
CFEKSAWFFVSIIAVNKAGATWVPLDPSHPLQRQQQVVSQTNATLALASTSNVDMCSKLVANVVEVSHALGERL
AKTEASSYGPISNVSPGNAAYVLFTSGSTGTPKGLVMQHRAVCTSQTAIAKRLGMTYNVRMLQFASFVFDLSIGEI
VGPWVVGGCLCVPSEETRMNTGSLVDFINTMKITWAYLTPSFTRTLKPDDIPDLELLLFAGEAVGRDVFEAWFGK
VRLINGWGPAETCVFSTLHEWKSIDESPLTVGRPVGGHCWIVNPRDPQRLAPVGTLGEVVIQGPTILREYLADSTK
TEAAIVRNLPEWVPNRTTNHWDRFYKSGDLCRYNADGTIEFGSRKDNQVKIRGLRVELGEIEHHFRESLEGVKQ
VAVDVVNGDGGAIIVSYFCFTDETRTAGKNSETNVKGVLAPMTSELQSQLTALVGQLSVTLPRYMIPTLFI

>Trichoderma _lentiforme_(18me)-mod10

ELAQKSNGAGPEIIDDCIHHIIARQVERTPNAPAIDAWDGQFTYGELDRAANRLAHFLINDYGVKIGDIVHVCFE
KSKWYIVAILAINKAGAAWAPFDPAHPLQRLQAVASRTDAKLALASTANTSLCEQVVEYVVEVSSTSDRELLNSY
GISEKGPDVHVTPTDAAYILFTSGSTGIPKGIVMQHKALCTSQAAISEWLGLDHTVRMLQFSSFVFDVSVGEIMQS
LMNGACVCVPSDHMRLNSLDGFVRDFNVTWAYLTPSFTRTLKPKDFPNLELLLLIGEATTQDVLDTWFGLPNTR
FINAWGPAETCVFSSLYEWHSNTESPMKLGRAVGAYVWIVDADNPQRLAPTGCLGEIVVHGPSLLKQYLADPEK
TVAATVTELPEWAPQRQSMGWNRFYKTGDLGFYDHDGILHFASRKDTQVKIRGLRVELGEVEHRIQGALEGVR
QVAVDIFKTEKGANLVAYFCFSDDTKTPAQSAGLEGRDIFLAIDTELKSKLDEMLAKLNSVLPTYMIPTMFI

>Trichoderma _lentiforme_(18me)-mod11

DVERAMIVNAEIPEVIDDCMHDLFENRANLNPQAPAIAAWDGNFTYSELNRAANRLAHHLINDYGVMPNDFV
HVCFDKSAWYIVSIIAISKAGATWVPLDPFHPEQRLQSIVSQTKAKLALVSPGNAALFSTLVENVIEVASALDSMLP
AQNGDKSPIVSVTSRAVAYVLFTSGSTGTPKGVMIQHGSLCTNMIAISERLRYHSNVRILQFSAYVFDFSILEIIASLL
QGACVCVPSEHVRMNSIVDFIRDMNINWLYLTPSFLRTIHPDDVPNVELVCAGGEAIPRDVFETWVGRVRFFNG
WGPTETTVMGSLHEFKSLDESPLVIGRPVGGFCWIVDPNNPQLIAPTGTLGEIVIQGPTLLQEYLDNPQKTQEAILY
DLPEWAPRPDENNWSRFYKTGDLCFYNDNGEIEFSSRKDTQVKIRGLRVELGEIEYHIQASLKSIRQIAVDVLKTD
NGSNLVAYLCFNDDIRQLHEAGTNGPFASFDTELQNTLIGAIAICPASHLIWSTILSVPSDS

>Trichoderma _lentiforme_(18me)-mod12

DLEQAKRWNVENPEILDTCIHTLIENQALVNPDAPAVCAWDGEMNYSQLNTAANKLAHHILKAGVETGDLVH
VCFEKSLWFFVSIIAINKAGAAWVPLDPSHPEQRLCQVVSQTLAKLALSSPANAVLCSKLVEPVIEVSPSFIDDISKI
YDGVNSPAVNVPSSNAAYVLFTSGSTGTPKGLVMQHSAVCTSQTAIATRLRLTPDVRILQFAAYVFDLSIGEIVAP
LIHGACICVPSEEIRMNGLEKFIRAKNINWAYLTPSFVRTLKPEDVPSLELLLLAGEAVGRDILTTWFGKVRLINGW
GPAETCVFSTLHEWSSVDESPLTIGRPVGGCCWIVDAEDSNKLTPIGCLGEVVLQGPTLLREYLADPQRSKEAIITS
LPPWVPKRNSNAQAWGRFYKSGDLCFYNSDGTLEFYSRKDTQVKIRGLRVELGEVEHHIRKSLEGVKQVAVDVL
KSETGTNLVSYVCFNDESRPVSSDLKANDIYLPLDVNTQTRITAMVGELSVTLPRYMIPTMFI

>Trichoderma _lentiforme_(18me)-mod13

WDVQHALASTQLKPSTESCTHWLIQERIKTQPNDPAIASWDGNLTYAELGIFASRLAWMLQSLGVGPESLIPLCF
PKSTWAVVTMVAIEMAGGAFVPLDPNAPVARLRGIIEDTKSSLAVASPSCQDTLRDIGIEVLAVDEALLIELSDPA
GGIKSVVQPDDASVILFTSGSTGKPKGMVIQHNNLCSSGNAYGSDLNIGPGTRVFQFSAYTFDVGVLDCLVSLMR
GATVCIPSDHARLNDLAGAINATKANWVFLTPTVADLLSPADVPDLKVLCLGGEAISKRCADRWIIHTEVHGLY
GPAEASICAWNPAVGKIGRSTNLGRPISSAFWVVEPTDYKRLVPIGCIGELLIEGPMLARGYLNVTAEVASNWMQ
DVNWLPGDSNKRVYRTGDLVRRNADGTFDFIGRKDTQVKLHGQRVELGEIESRIHEFLPENMDAIVAVVKDEQE
HDNFAFLWYTEGEVAAQSTLHLLETVTDDARSTISDIDSSLGMVLPSYMIPSSYLLFEGKPEQ

>Trichoderma _lentiforme_(18me)-mod14

DLEFSRAANHDEPAVIDDCIHNLIEQQALINPSLEAISAWDAKFTYAELDLSANRLANYLIQSMGVRLGDFVHVC
FEKSAWYVVSILAINKAGAAWIPLDPSHPAERHQQIVGQTGSKLALTSTANFDKCVGLVARVLEVTEEFVDTLAD
QYNDSKPFTNVTPQDVAYILFTSGSTGVPKGVVIEHGALCSSQTSYSRRLGHAPGVRMLQFSSFVFDASIAEIYAPLI
SGACVCIPSWETQMNSLAGYICEENVTWALITPSLARTIDPHEVPCLEVLMLGGEAVSQDVFELWFGKLRLFNAW
GPTESCVFGSLHEWESTNESYMTIGRPLGGYCWIVDPEDPQKLAPTGTFGEIVIQGPNLLREYLADKDKTASAIVTS
LPDWAPHRHSRHYNRFYKTGDLARYNPDGTIQYYSRKDTQVKIRGLRVELGEVEYHIRQNLDGVRQVAVAVFET
DAGVNLVSFVCFNNDMLPASMTSNLTSDDIVLQLTGELKDNIASLLGLLNVVLPGYMVPTLFV

>Trichoderma _lentiforme_(18me)-mod15

AQCLKFNSEIPDIIDSCVHQLIENQAKIRPDAMAIRAWDMDLTYYEFDRAANRLANYLTASYNIKTDDLIHVCFE
KSAWFFVSILAINKAGAAWVPLDPTHPEQRLRQVVSQTRATIALTSVANRDLMSGLVESVVTVSSELDLLLSEIEE
NSQRGPATTVASDNAAYVLFTSGSTGIPKGLVMQHGSVCTSQTAIVKRLGLTPDVRMLQFAAFVFDLSIGEIVAPL
ITGACLCIPSEHTRINNLTQFIREMDINWTFLTPSFIRTINPAEVPGLELVLLAGEAVPKDVLATWFGKVRLINGWG
PAETCVFSTLHEWKSVNESPLTVGKPVGGFCWVVDPADPHRLAPTGTIGEVVIQGPTLLREYLSDPERTKSSTVYN
LPNWAPRPDAEHWNKFYKSGDLCYYNQDGTIEFATRKDTQIKIRGLRVELGEVQYHIQQALPTARQVAVDVYQ
GENGISSDASDGLFLPIDEDLQSQLASVAGQLRISLPRYMIPTMFIPCSYMPFITSTKLDRNKL

>Trichoderma _lentiforme_(18me)-mod16

DLHRATGFNSEFLEIMDTCIHHLIELRAKEAPDSPAIWAWDGELTYGQLNETANKLAHYLINEYDVQVEDLIHVC
FEKSIWYWVAIFAINKAGAVWVPLDPSHPEQRIRQVTLQTQSKLALVSNNTRSLISGIIDQVVEVSPALYEKINSSL
GANDPQIPVSSHNAAYVLFTSGSTGVPKGLVMTHGGFATSQTAIRKRMGISSSNRTLQFASHVFDLCLGESILQLIS
GACMFIPSEHTRMNGLKEFIAEHEINTLLLTPSFVRTLSPDQIPSVTLLLLAGEAVPRDILTTWFGKVRLWNGWGP
AETCLFSSLHEFQSVDESPLTIGRPVGGFCWVVDPTNPNKLAPIGTMGEVVIQGPTILREYLADVERTKATTMYEL
PAWAPYRDQPSWSRFFKSGDLASYNTDGTLEFSSRKDTQVKIRGLRVELGEVEYHVRSGLEGARQVAVDVFTTDS
GTRLITYICFSDETRTVGISQSDEDIFLPITEELQTQLAKMISQLNVTLPRYMVPSLFVPC

>Trichoderma _lentiforme_(18me)-mod17

PWDVQQAVQLNSKKPDFIHTCLHDMFAEHASNSPHHEAIYSSEGSLTYGELDHLTDIVATHLSSLNVGPETVVPF
CFEKSMWAVVAILSILKAGAAFVPLDPSHPIGRRETLVREVNARVLIASSNAIASCAGMAEHIVELSPSVISKLATSI
TPKVLPKVGPRNTAYVLFTSGSTGKPKGVVMQHGSFSSTTIGYGKAYNLSPSSRIFQFSNYIFDGSLGEIFGPLAFGG
TICIPSDDERLQSAPDFMHKARVNTAMLTPSFVRTFTPDKVPHLKTLVLGGEAASKSILEMWVDRVTLFNGYGPA
EACNYATTHMFKSSAESPRIIGSGFNGACWVVEPDNHNKLVPIGCIGELVLQGHALARGYLNDKVRTEESFVSEI
GSLPSSLLHEPKRFYLTGDLVRYNVDGELEYLGRKDSQVKLRGQRLELGEIEYTITQSLESVCHVAVDVLHRQAGD
ALIAFVSFIGDASAEWVTDDLRLNLLVSDDAMRATLGSLRESLKATLPSYMVPSIILPLQ

>Trichoderma _lentiforme_(18me)-mod18

WELQKQLEWNNKIYGPADITLHGLFSKQAARRPDHEALYSTEGNMTYSELDHLSTQLAAYLSRLGVRPETIVPFC
FEKSIWTIVAMLGILKAGGVFIPLDPSHPTSRRQALIDEVCAQFMIVSPTTAPDSQDMVKNTVELSPSLVASISAIDT
AKQPFIIPGPDNAAYVLFTSGSTGKPKGVIIEHKAISAALLRQSEACSINSDVRMLQFANYVFDGSIFEIFNALIMGA
TVCVPTEHERLHNAAAFIRGARVNYAVLTPTFIKTLSPEQVPGIKTILLAGEAPSREIIETWVD-
EVHLHNGYGPAEGCIGSTVFAYSSTSTASATTLGRSFAHGLWVVDPDNHDQLMPIGCVGELLLQGSSLARGYIND
EKKTRQSFIEEVKWLPSNIDVGERRFYKTGDLVRYVSDGSIEYLGRKDTQVKIRGQRIELGEIEYHIKRSDTAIEHAV
VDITRKDGRDSLFAFICLNSQQEAESASQEIHLTELTEELYEMFSSIVTSISSSLPSHMIPKYFIPVDHM

>Trichoderma _lentiforme_(18me)-mod2

EQSLQANNDIPEIIDACFQELVDKQAIERPNSLAVVGWDRTFTYIELVEAVNRLSNYLATNFTFKADELIPVCFEKS
SWYIVSILAINKIGAGWVPLDPSYPEQRLRQIVGQTRAKIILCSPSNAHICNSFGLSVIEISPFFYQKLTESGVTGTTGP
AIKVTPRNICYVLFTSGSTGTPKGFVMEHGAVCTSQTAISRRLGMTADVRMLQFAAFVFDLSVGEIVATLISGGSV
YIPDENTRLNDISNFIQKHKINSTILTPSFARTLKPEDVPSLELMVLAGEAVGRDVCDSWYGKLRFICGWGPAETC
VLSSLHEWQSIDESPRTIGRPVGCFCWVVDPENPKRLAPTGTVGEIFIQGPTLLREYLADSEKTRSSMVYDLPAWVP
RRESQYWSRFYKSGDLGYYNPDGTLEFSTRKDTQIKIRGLRVELGEIEYYLQQAIDEIRQVAVDVLSGDNGSNLVA
YFCFNEDTKTADARSQADDKGPFMSIDEDLQTRLIAASGELKVVLPSYMVPAFFI

>Trichoderma _lentiforme_(18me)-mod3

DLAEASNGDGPEIVDDCAHLMIERQTELTPQAHAVHAWDGTLTYSDLDRAANRLANLLVDSHGVKVGDIIHVC
FEKSLWYVVSVLAINKAGAAWVPIDPAHPFHRLQQVASQTGAKIALTSSIHRPLCSKLLDTVIEVSSNLDEQLNN
DTDIKDVKPSVAVASNDVAYLLFTSGSTGVPKGIIMEHGSLCTSQRDIAKRLGLKRSVRMLQFSSFVFDVSVGEIML

SLMHGGCVCIPSDHDRLNNLAKFIRDTEVSWAFLTPSFARTLRPTDVPSLELIVLAGEPVTQDVFDLWFGKTRLVN
GWGPAETCVLSAIHEWKSGDESPLTIGRSVGSFAWIVDAENPQRLAPVGCIGEIVMQGPTLLREYLADPAKTLSST
LTSLPNWAPRSNDRNWSRFYKTGDLGIYNPDGTIHYSGRKDTQVKIRGLRVELGEIEHHIRNSLEVIQQIAVDVLR
TEAGANLVSYICFSNETKTPGPDTDPEGTDIFLPMTESIQKELNIVLNKLNVLLPRYMIPTYFI

>Trichoderma _lentiforme_(18me)-mod4

DLDRSIYFNSETPEIINACVHELIERQAECRPDAVAISAWDAEFTYNQLNCAANRLANYIIKSYGIQPDDIIHVCFE
KSIWHFVAILAINKAGAAWAPLDPSHPEQRLRQIVDQTRASLIMTSPSNSDLCSALVENVLLVTPNLDRKLAKTT
DSEAPNVVANPDNAAYILFTSGSTGTPKGFVMDHKSVCTSQTAITKRLGHTSNVRMLQFASYVFDMSIGEIISTLIS
GGCIFVPSEDIRMNNITQYMRDNRINWAFLTPSFLRTLAPKDIPDLEILLLAGEAVPREILNTWFGKVRLINGWGP
AETCVFSTLHEWKSVDESPLTIGRPVGGFCWIVDPENPHKLAPVGTLGEVVIQGPTLLREYLADPERTAASCIKAP
KWTPQPDSQHWGRFYKSGDLCSYNADGTLEFSSRKDTQIKIRGLRVELGEVEYHIQAALQDVRQIAVDVYNDER
GTNLVAYICFTDEIRVAGINHDTNSPFFSIDQKLQNRLNALVDRNELRRHTLLLTREQLSEY

>Trichoderma _lentiforme_(18me)-mod5

IQQAIAWNTENPEIIEDCIHKLIERNAASTPNAVAIDAWDGMLTYAQLDQMANRLAHYLIKTFDIKSDDLILCLFE
KSLWYVVTVIAVNKAGAAWVPLDPSHPTQRLQQVARQTKANLLLASSLQLNLGNQLSHTVLEVGQALDDVLIQ
TEPSTQAPDVTVSTRNAAYVLFTSGSTGIPKGLVMEHGAVCTSQTAITARIGLHSGVRLLQFGAHVFDLSVGEIFCS
LIRGACLVIPSDEIRMNSLAKFIHEKNINWAIFTPSFIRTLEPTDVPHLELVILCGEPSDKKILEKWVGKVRLFNGWG
PAETCVYSSLHEWKSTAESPLTIGKPVASFIWIVDPKHPNRLAPVGTIGEAAIQGPTILREYLDDPTRTQASILELIPD
WAPPREPQQRWNRVYLTGDHCSYNPDGTIEYHGRKDTQIKIRGLRVELGEVEHQIRKLSENLVHVAVDVHKFEA
GSMLVAYLGYSEKNSDDPDAIFMPLNNNLQRDLDAMASQLGVLLPRYMVPTLFIPCSQMP

>Trichoderma _lentiforme_(18me)-mod6

DLQQAISYNVKEPGMVSVCVHELITQQALRDPHHEAIYSNEGTMTYATLDRLSSLLAQYLHELGVRPESVVPFCF
DKSSWAIVAMLAILKAGGVFLPLDPSHPRNRREALVQEVGAEIMIVSPSSSVPCEHLTSTMVELTPQLLEELSSIYD
AFQEIQPKAKPSNAAYVLFTSGSTGKPKGVLMEHSAFATSTLGHGRVYNLSPSSRVFQFSNYIFDGSLGEIFTTLSFG
GTVCVPSDDERLQGAPPFMRRARVNTAMLTPSFVRTFTPEQVPSLQLLVLGGEPSSKDLLETWCDRLRLVNGYGP
AEACNYATTHDFKATDSPRIIGRGFNSACWIVDSTDYNKLTPIGCIGELVIQGNALARGYINDADRTAASFITHID
CLPKSTIPGPHRFYLTGDLVRYNVNGDLEYLGRKDTQVKLRGQRLELGEIEYHVKRSLSDIEHVAVDVVHREAGD
ALIAFISFKEKMASSGNILLLNDDLRLALATIMEHLKMSLPGYMVPTTILPVKRMPFITS

>Trichoderma _lentiforme_(18me)-mod8

DQALKLNSRTPNAVERCFHEMVDEVASVRSDSLAIFGWDRSFTYKEMTETTNRIAQYLVAEHGVKVGDVIHVCF
EKSAWFLIATLAINKAGAAWSTLDPSHPTERYQKIISQTGSQLALASAINSHRCIGLLPKVIELTPELDAKLALNSQ
WSAKGPAVKVTPSDVAYILFTSGSTGVPKGVVIEHASLCTSQTSLSQALQFQEDVRVLQFSSYSFDAALFEIGSTFLT
GACLFVPSWDEQMNELTEYIRKHQITCALLTPTLARTIRPEDVPSLNMLLAAGEAPTRDILDIWFGKVRLLNAWG
PTECCVIACLHEWTSVDESPKVIGRPIGGSCWIVDPDDSTRMAPLGTVGEIVIQGRNLLREYLSDPVKTAAATVTG
LPQWVPKRDSLHWDRFYLTGDLGFVNESGNIEYCTRKDTQVKIRGQRLELGEIEHHIQANLEGVRQVAVDVVRS
DAGSALVAFVSFSDAKEPISLDEATVGNGIFLPLDASLQATISSLVGTLGTLMPRYMVPSAFI

>Trichoderma _lentiforme_(18me)-mod9

LANSMKQNSDIPDIVESCVHQLIERQAAAQPDALAIVSWDRDFTYRQLNEASNRLAHLLVSRYNVQPEDLIPVCF
EKSAWHFVAITAINKAGAAWVPLDPSHPELRLRQVVSQTRAQLALSSSANSKLCGILVGEVVEVNAELDNKLLA
TEASENGPIVAISPRNAAYVLFTSGSTGTPKGLVMEHGSVCTSQVAIAKRLGLNSKVRMLQFAAFVFDLSIGEIIGP
LISGACICVPSEDTRMSGIVDFINTKAVTWAYLTPSFVRTIKPSEVSQLELLLLAGEAVPRDVFTTWFGKLRLINGW
GPAETCCFSALHEWQSAEESPLTVGRPVGSFCWIVDPENPCQLAPTGILGEVVIQGPTILREYLSDKERTDTAVVKS
LPEWAPFREQANWSRLYKSGDLCKYNSDGTIEFSSRKDTQVKIRGLRVELGEVEHAVQVALRGVRQIAVDIFKGE
YGTNLVAYFSFSDETRQIQEADPSGPFQPMDENLQARLTAVVGELNIALPRYMIPTLFIPCK

>Trichoderma _lentiforme-_mod14

DVEQAIERNSYSIEPIESCVHELISIQAAKNPQQEAIYSTSGSMTYRKVDRLSNQLASHLIRLGVKSETIVPFCYEKSV
WSIIAMLGIIKAGGVFLPLDPSHPLSRRQALIDETSAQYMIVSPTTAKECKGMTKNNIELSPNFFSKKKRSSTARKFK
PIKPRPGSAMYILFTSGSTGKPKGVIIEHKAISSFLVKLCEVVQFSGNSKMLQFSSYGFDASIFEIFAVLISGGTVCVPT
ETERMQHTSTFIEEAKINTAILTPTFVKTFSPESVPSLKTLFLCGEAPSKEVVDLWCQHVSLWNAYGPTETCVIATL
QHYTDVSVPVTTIGRGFAHHCWVVNPENHHELTPIGCVGELLIQGDSLSRGYINDEEKTNNAFINHVEWLPANV
DVGKRRFYKTGDLVRYNPDGSFNYLGRKDTQVKIRGQRIELGEIEYQVKLHLPDLEHAAVDIIRHDTHESLVAFVS
FSGDKGPSEVARVQLVSHNDELQELFSQLVTNLTTVLPSYMVPKYFIAVESMP

>Trichoderma _lentiforme-mod_10

YELAIASNNVLPEIVDSTVHELIDRRARASPDAPAISSWDAKFTYSELISASNKLAYHLIHAHEIKPDDFVHVCFEK
SAWYIVSIIAINKAGAAWVPLDSSQPEERLFQIVRQTKAQYALVSPNNAALCTKLVKHVIEVTVDLDKHLSLSVPD
TYVPCVASSSRSAAYILFTSGSTGTPKGVVIEHKSVCTSQVAVADRLGITPSVRILHFAAHVFDMTIGEIIAPLISGAC
ICIPSNRDRMENIVNFIKEQRVNWIFLTPSFARTIQPEDIPNVEVLFLAGEAVPQDLFLTWVGKVRFFNGWGPTETC
FISTLQEWTSAEESPLVLGRPLGGFCWIVEPDNPQRLAPIGTIGEVLIQGPTLLREYLGDPQRTQASLIPRPTWAPLP
DSQHWSRCYRSGDLCFYDKDGNIVFSGRRDTQVKIRGFRVELGDIEYHIRDKLDATCEAAVDVLKTTTGASLVAF
VSFNTQAQQPSTGNDDSSDGIFLHLDSGTQEIFGTLLLHLKALLPPYMVPTTFVP

>Trichoderma _lentiforme-mod1

DLQRAIECNSEEPDIISSCLHQIIEHHALKRPDAMAVHASDLTLTYSQLNLAADRLSSYLMDNCAVQARDLVHVC
FEKSAWFFVSILAINKAGAAWVPLDPSHPLQRHKKVLMQTKAKLALASPSNMPLCADLVENVVEVSATLDEML
MRRRQGNKNPRMVSPSDACYVLFTSGSTGTPKGFVMQHQALCTSQTAVAKRLGMTSDIKVLQFASYVFDLSIGEI
VGPWIAGACICVPSEEVRMNGLAEYIRTMDINWVYLTPSFSRTLIPDDIPGVELMLLAGEAVSRDVLETWFGKLRL
INAWGPAETCVFSTLHEFGSLDDSPLTIGRPVGGFCWVVDPENPHRLAPFGTIGEGVIQGPTLLREYLADPAKTKS
ATVKPLPSWAPNRSSDQWNRFYKSGDLCKYNSDGTIAFVTRKDTQIKIRGLRVELGEVEHQIKACLDGVHQVIVD
KIDNEAGASLVAFFCFNNETKIVNTAGGDPTMFLPITNQLKSQIVGMIGHLSVNLPRYMVPSLFIPC

>Trichoderma _lentiforme-mod11

FYHKVNGEALPEILDTCLHKLIELQAEACPDAMAIQGWDKTFTYYELNRAANRLAHHLVAEIGVKPHDIVHVCF
SKSAWYVVAILAINKAGAAWGPIDPSHPLQRHQQIVGQTKARVALASPDNVERCSELVPSVVAVSSSLDSNLSEK
NYDSRRGPDVTVTALHAAYVLFTSGSTAVGKRFGFTPKVRILQFTTYVFDFSISEIIAAMLFGACVCIPSEETRMNRL
KDFIQESGINWLFLTPSFLQTLKPDEVPNVELVAVGGEPLPRALFEEWVDKVRLFNCWGPSETCVMSAMHEYKSA
DESNMTIGKPVGGFCWIVDPEDPQRLAPAGAVGELIVQGPTLLREYLNDPAKTHASILCTRPDWALCPDTEHWN
RFYKSGDLCSYNPDGTIQFHSRKDTQVKIRGLRVELGEIEYRIKEALHGALQVAVDVFKSDTASLLAAYFCFSDAT
AVISGTDVNDVFMPLTEEVQHHIAAMVGEIKILLPEYMIPTLFIQCR

>Trichoderma _lentiforme-mod12

MQQSHKLNSDVPEIINSCFHHLVEDQATYRPNAMAIRSWDGDFTYAELNQAANRLANHLVQTYDIETNELIHV
CFEKSAWYFISILAINKVGAAWVPIDPSHPVQRLREVVNQTKAKIALCSPEYIDLCFSLVESVVQVAPELDSQLLQL
VNSQKGPANRVSPDNIAYVLFTSGSTGTPKGLVMQHKSVCTSQIAIARRLGLTPEVRMLQFAAFVFDLSIGEIIGPL
ITGACLCIPSAHSRVNELPKFIQDTNVNWAYLTPSFVRTLKPTDVPSLELLLLAGEAVPRDVLTAWFGKVRLINGW
GPAETCVFSTLHEWKSIDESSLTIGRPVGGFCWVVDAENPDRLAPFGTIGEVVIQSPTILQEYLADPERTNASTMPS
TEWAPRPDGTNWNRFFKSGDLCRYNADGTLEFSTRKDTQIKIRGLRVELSEVEYHIQQALTGVQQVAVDLFKGD
SGSNLVAYICFNSETRTAGIQVDKVETPFTSPDESLRNQLVALAGELALALPRYMIPTLFIPC

>Trichoderma _lentiforme-mod13

LLSPWDLHHAVESNRLNPSSETCAHWLIQETIQSRPNDIAIASWDAELTYSQVGTFASRLAVKLQESGVGPETLVL
LCFPKSAWAVIAMVAIEMAGGAFVPLDPAAPAARLKGIIDDTHAILAIASPSAGTVLEGLSVEIMLVDQEVLSALP
DPTHQVVSNVQPQHASVILFTSGSTGKPKGMVIQHNNLCSSSDAYGDKLDIGPGTRVFQFSAYTFDVGILDCLVSL
MRGACLCIPSDHDRMNNLAGAMRSLKANWVFLTPTVADLLSPTDVPDLKVVCLGGEAISKKCADRWVNHVEL
HGLYGPAEASICAWNPTVGKSGRSTNLGRPISSAFWVVEPSDIRQLVPVGCIGELLIEGPMLARGYLNVNAEVAA
NWIDDVDWLPVEKKRVYRTGDLVRRNADGTFDYMGRKDTQIKLHGQRVELGEIESQIHHFLPDNMAAMVDIL
KADDENAHDILLAFLWYTEEDTAQSQSLSLMQSLPEKTQRFIRHLDSSLAEALPSYMIPSSYLIFEGKPE

>Trichoderma _lentiforme-mod2

QRSMKWNDHKVVIEEHCMHDVISRRTESCPNDEALYSSEGSLTYAALERLSDLVAYQLLQYNVQPETIVPFCMEK
SIWVVVAMVGILKAGGALMPLDPSHPESRRLALIQEVNAKVIITSPSTALSCEGMAQHTVQVSSSLLTHAPKTTKS
YRELSNYKKPEPHNAAYVLYTSGSTGKPKGLVMPHSAACTSLLRHPEKFSINKSTRTFQFSAYVFDVCIEEIFVSLFV
GATICVPTETERVGNTSRFMTEARVTWTCLTPSFIGTLDPDTLPTLQTLCIGGEAPTRDILSKWHGRAEIINAYGPA
EACVDVAGHVFKSRDDSPTNIGRPFAHKLWIVEPDNHNRLTPIGCIGELVIEGHAIARGYINNEEKTKESFVDDLE
WLSSATPSDRPRVYKSGDLARFNPDGTIEFFGRRDTQVKIRGQRLELGEIEYNIRAKLADVQHTVVDLIEREAGKM
IIAFITFKNYLCDTMTDIADPYGNFIKDVKLTSSFQDLMLELRRVLPSYMIPSIIFPLR

>Trichoderma _lentiforme-mod3

IEFARAANPNAPHVIDTCIHTLIEERTKISPKSAAVCSWDADYTYSELDDNANRLANYLMNVLKVKVGDLVMVC
FDKSAWYIVAILAINKIGAVWVPIDPSHPTERHQNIVKQTRSTLALCTPSNSLKCSQLVAEVLEVNAEFISKLRGSE
TYAGKPDVKVSPDDVSYIIFTSGSTGVPKGVVIQHRAICSSQVSLSQRLGMHDGVRMLQFSSFVFDASVFEILCPLV
SGACVCIPSWDMQLNSLPAFIRDTNVTWAILTPSLANVISPQDVPCLEFLALGGEAPSKEVFNIWFGKVRLFNAW
GPTEAAIIAATQEVQSTDLSHLTIGRPICGNCWIVNPEDPTKLAPVGTVGEIVIQGPNLLREYLADPEKTAAATVTP
LPRWTPNRELWNRFYRTGDLAVYNSDGTIKYCSRKDGQIKIRGLRIELGEIEHHIRKSLDTTGQVAVEALKSEAGA
NLVAFICQNSDTIPASMANNMTSEDIVLPLTDELKSSLGALRSSLISSLPGYMVPAFFIPC

>Trichoderma _lentiforme-mod4

QKAISFNPEMPEVVEKCFHQLFESQALETPEATAICAWDRSLTYAELDSTANRLAHYLMAQHAVKLDELIHVCF
DKSSWFFVSILAINKAGAAWVPLDPSHPPERLRQIVTQTKAQVVLTSESHAALCSGLVPLVVEVSDSLDRQLLRND
GYSSSSPVTKVSPNNAVYVLFTSGSTGIPKGLVMEHRSVCSSQRAIIKRLGLHSNVRMLQFAAFVFDLSIGEIIAPLIS
GACICVPSEQARMNDIAGFIRETGVNWAYLTPSFIRVLKPEDVPTLELLLLCGEVTPRDVFNTWVGKVRFISGWGP
AETCVFSTLHEWKSNTESPLNVGKPVGAFCWVVDPERPDQLAPIGTVGEVMLQGPTLLREYLDDPERTRLSVITSL
PNWALQRAPQWTRFYKSGDLAMYNPDGTIEFCSRRDTQVKIRGLRVELSEVEHRIRESLEGIRQVAVDVLTSDGG
SNLVSYLCFSEEIRSSSDAQANLDDIFLPLTAELQPLLAAMIGQLKVVLPNYMIPTLFIMC

>Trichoderma _lentiforme-mod5

VGDWDIKHAVDSQLSITSTQSCTHWLIQDAIRAYPDSPAVASWDGELTYAELGTLVSRLSTKLQQLGVGPEVLVP
VCFPKSIWAVVTMIAVEMAGGAFVPLDPKAPQSRLKSILDDIKATIALTSPCCESTMKDLDVSTVAIDGPFLDTLPE
ATVPSQHSGSPSDAAVVLFTSGSTGKPKGMVIQHDALCSSAAAYGHDLQIGPGTRVFQFSAYTFDVGVLDTLVTL
MRGGCLCVPSDHDRLNDLSGAINRTKANWVFLTPTVADLLNPADVPTLRVVCLGGEAISQKTADRWKDYADL
HGLYGPAEASICAWNSTVGRSGKSTNLGRPLSSAFWVVEPSDIHNLVPVGCIGELLIQGPMLARGYLNVCAEQA

	ANWIEDFSADWLPHGFPNRAYRTGDLVRRNADGTYDYIGRKDTQVKLHGQRIELGEIESQIQKALPAEMTAIVD<br>VVKSHVLAFLWYTHETAPNKQSSLKLINTLSEAQKVLISDIDMTLSIALPSYMIPSSYLIFDGKPDHTT
>*Trichoderma _lentiforme-mod6*	LEKAIAVNHEIPEFMDTCLHTLIEIQADQRPNAIAISASDGEFTYSQLNEAANRLAHHLIKTYSVEVEDLIHVCFEK<br>SIWHVISVIAINKVGAAWVPLDPSHPEQRLQQIIDQTGARLALASTTHSALCAKVIKNVLEVNSIIDDQLVDAGVS<br>SSNPAVTVSPRNTVYVLFTSGSTGTPKGIVLEHAAVCTGLNAISKRLGLPATARMLQFSAFTFDVSVGEIFTSLISGA<br>CVCIPSDWARMNNITGFIQENHVNWAYLVPSYIRTIDPEEVPSLEVVALMGEPATKDVYNAWFGKVRLFNSWGP<br>TETVVISSSHEWKSANESPSVIGKPLGTYCWILDTRNPTQLAATGIVGELVVQGPTLLREYLAAPDKTKASIISDLPN<br>WIPRQALPGCDRLYKTGDLCFYNPDGNIEYFSRQDTQIKIRGHRVEAGEIEYQVSQAMTVDQVAVELANTESGST<br>LVAYLCFNLDCKPISPTATPDEIFIPLTDDMQQLISTAVAQLKTTLAHYMVPTTFIVCK
>*Trichoderma _lentiforme-mod7*	EHDFAASANGIPPVAIKQCIHSLIEEEAKKHPESYAIQAWDADFTYQQLDICANRLANYLISSLHVKIGDIVHVCF<br>DKSAWYIVAILAINKAGATWSPLDPTHPVRRYQQTISQTGSSLILTSPTNASKCATLADSILEISSGPYETLVKDVTE<br>RRPDVAVAPEDAAYILFTSGTTGVPKGVVIEHQSLCTSQTALAQRVGFTNDTRMLQFSSFVFDASVLEIIAPLVMG<br>GCVCMPSWDEQMNDLAGFIARASVNAVFATPTVARSLRPEEIPGVERLVVGGEAAASDILETWIGKLRLFNAWG<br>PTEICVAGSVHEWKDMDESPMTIGRPILGNWFIVDPNNPMRMVPTGCVGEIVYQGPTVFREYLANPSQTQEVIVS<br>SLPPWAPNRNMRDWSRFYRTGDLGRYNPDGTMEYLGRKDTQVKIRGLRIELHEIEHQIFAHLPRVRQVAVDVM<br>KREETSHLVAYYCISNGTLPSHQAVDDAGKGVFLPLRVELMPQIQDLIAHLKGVLPPYMVPSTFI
>*Trichoderma _lentiforme-mod8*	VEQSMKFNPEVPEIMDTCVHTLIEAQAHREPNALAISAWDGDLTYRQLNESANRLAHYLVKNYNIQPDTLIHVC<br>FEKSVWHFVAIIAINKAGAAWVPLDPSYPEQRLRQVISQTRSTLVLTSSRNAKLCSNLISDVLQIDVELDKRLILTED<br>GHKGPTVAVTYRHAAYVLFTSGSTGKPKGLVMEHGAVCTSQTAIAKRLNLASDVRMLQFAAFVFDLSIGEIIGPLI<br>SGACLYIPSEDTRMNGLKQYVNKTGVNWAYLTPAFVRTFVPEDMPGLELLLLAGEAVPREVLSTWFGKVRLVNG<br>WGPAETCCFSTLHEWSSVDESPLVVGRPVGGFCWIVNQDDPQKLTPTGTLGEIVIQGPTILREYLADVERTKDSTV<br>YALPKWAPRPDSEHWNRFYKSGDLGYYNPDGTIVFSSRKDTQIKIRGLRVELGEVEHYIHETLEGVCQVVVDVYQ<br>KEGRSNLVAYICFNEEMKNPSADEEFTAAALFASITDDMRQRIAVMLGELRITLPQYMIPTLFI
>*Trichoderma _lentiforme-mod9*	SWDLQQALDASRLRPATESCTHWLIQDKIKEQPEETAITSWDGDLTYVQLGVHATCLAAKLQDLGVGPESLVPIC<br>FHKSKWAVVSMVAVQMAGGAFIPLDPSAPPARLQGILQDTGAALAISDPSSQATLQSLGVEVITVDNETISKLPSS<br>VALQCEVQPANASVVLFTSGSTGKPKGMIIQHNSLCSSSDAYGSDLNIGLGTRVFQFSAYTFDVGVLDCLVSLIRG<br>ACICIPSEHDRLNNLATAITASQATWVFLTPTVADLLHPVDVPTLKTVCLGGEAISKKCAERWVNHVDLHGLYG<br>PAEASICAWNSMVGKSGRSTNLGHPISSAFWVVDVNDPKSLVPLGCIGELLIEGPMLARGYLNVSADVAANWM<br>EGVDWLPGSKQPRRIYRTGDLVRRNADGTFDYIGRKDTQVKLHGQRVELGEIEAWVNEFLPDNMAAIVDVAKD<br>NDGPDSLLAFLWYTEEASNSPARLMDVVSDEARAAILHLDTSLSTILPPYMIPSSYLLFGGKPEQT
>*Trichoderma virens_Aib_mod12*	YSQLNSAANQLAHHILKIGIKADDLVHVCFEKSVWFFVSIIAINKVGAAWVPLDPSHPEQRLRQVVGQTLAKFAL<br>SSPTNAALCNKLVHNVIEVSPSLIDELSKFCDGFNSPAINVPSSNAAYVLFTSGSTGTPKGLVMQHGAVCTSQTAI<br>AKRLSLTPDVRILQFAAYVFDLSIGEIVAPLIHGACVCVPSEETRMNGLKEFIRDARINWAYLTPSFVRTLRPEDVPS<br>LQLLLLAGEAVGRDILDTWFGKVRLINGWGPAETCVFSTLHEWSSIDESPLTIGRPVGGYCWIVEAEDSNKLTPIG<br>CLGEVVLQGPTLLREYLADPQRSKETIITELPPWAPKQVSDAHLWSRFYKSGDLCFYNPNGTLEFYSRKDTQVKI

Table S2. Putative BGCS found in the genome of *T. endophyticum* MMSRG85.

Region	Type	Most similar known	cluster	Similarity
Region 1.1	Terpene	-	-	-
Region 1.2	Fungal-RiPP-Like	-	-	-
Region 1.3	Fungal-RiPP-like, NRPS	choline	NRP	100%
Region 1.4	Terpene	-	-	-
Region 1.5	Terpene	-	-	-
Region 1.6	Fungal-RiPP-like, NRPS-Like	-	-	-
Region 1.7	NRPS-Like	-	-	-
Region 1.8	NRPS	-	-	-
Region 2.1	T1PKS	-	-	-
Region 2.2	T1PKS	clavaric acid	Polyketide	100%
Region 2.3	NRPS, indole	-	-	-
Region 3.1	T1PKS	-	-	-
Region 3.2	T1PKS	harziphilone/t22azaphilone/isoharziphilone-1/isoharziphilone-2/compound 4/compound 1	Polyketide	70%
Region 3.3	NRPS	-	-	-
Region 3.4	T1PKS	decumbenone a/calbistrin A/calbistrin C/decumbenone B/decumbenone c/dioic acid moiety	Polyketide	15%

Region 3.5	T1PKS	-	-	-
Region 3.6	Terpene	squalestatin S1	Terpene	40%
Region 3.7	Terpene	-	-	-
Region 3.8	T1PKS	-	-	-
Region 3.9	T1PKS	-	-	-
Region 3.10	Fungal-RiPP-like,	-	-	-
Region 4.1	NRPS	-	-	-
Region 4.2	NRPS, T1PKS	-	-	-
Region 4.3	NRPS, T1PKS	-	-	-
Region 4.4	Terpene	trichobrasilenol/xylarenic acid B/brasilane A/brasilane F/brasilane E/brasilane D	Terpene	60%
Region 4.5	NRPS	-	-	-
Region 4.6	NRPS, like	-	-	-
Region 4.7	T1PKS	-	-	-
Region 4.8	T1PKS, NRPS	lucilactaene	Polyketide	38%
Region 4.9	T1PKS	-	-	-
Region 4.10	T1PKS, NRPS	-	-	-
Region 5.1	T1PKS	-	-	-
Region 5.2	T1PKS	-	-	-
Region 5.3	TIPKS	1,3,6,8-tetrahydroxynaphthalene	Polyketide	100%
Region 8.1	NRPS-like	-	-	-
Region 8.2	T1PKS, NRPS, betalactone	harzianopyridine	NRP+ Polyketide: Iterative Typi I polyketide	60%
Region 8.3	NRPS	-	- I	-
Region 9.1	Fungal-RiPP-like	-	-	-
Region 10.1	NRPS	-	-	-
Region 11.1	T1PKS	depudecin	Polyketide: Iterative	33%

Region 11.2	T1PKS	tricholignan A	Typi I polyketide Polyketide: Iterative	88%
Region 12.1	T1PKS	trichoxide	Typi I polyketide Polyketide	58%
Region 12.2	T1PKS	-	-	-
Region 12.3	NRPS-like	peramine/intermediate 1/intermediate 2	NRP	100%
Region 13.1	T1PKS	YWA1	Polyketide	100%
Region 13.2	NRPS-like, T1PKS	dichlorodiaporthin	Polyketide	66%
Region 13.3	Terpene	karaiol	Terpene	100%
Region 15.1	NRPS	-	-	-
Region 17.1	NRPS	metachelin C/metachelin A/metachelin A-CE/metachelin B/dimerumic acid 11-mannoside/dimerumic acid	NRP	62%
Region 17.2	NRPS-like	-	-	-
Region 17.3	T1PKS, NRPS	-	-	-
Region 18.1	T1PKS, NRPS	Phyllostictine A e B	NRP + Polyketide	40%
Region 19.1	T1PKS, NRPS	-	-	-

Figure S1. Synteny analysis between BGC 8.3 of *Trichoderma endophyticum* strain MMSRG85 and BGC related to the NRPS2 gene containing an NRPS that performs the biosynthesis of 14mer and 11mer peptaibols in *Trichoderma virens* Tv29-8.

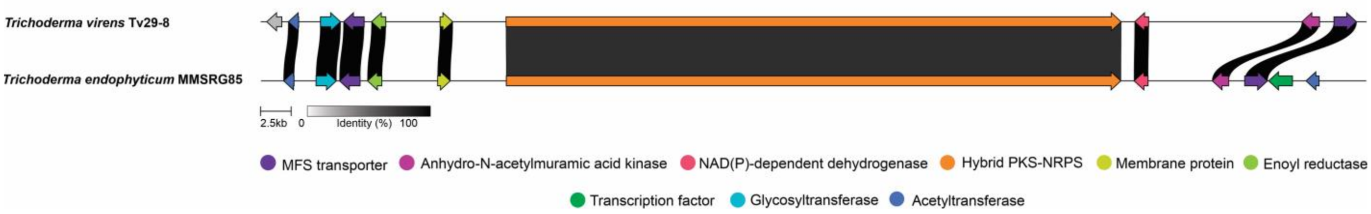

Figure S2: Phylogeny of 14-res NRPS adenylation modules.

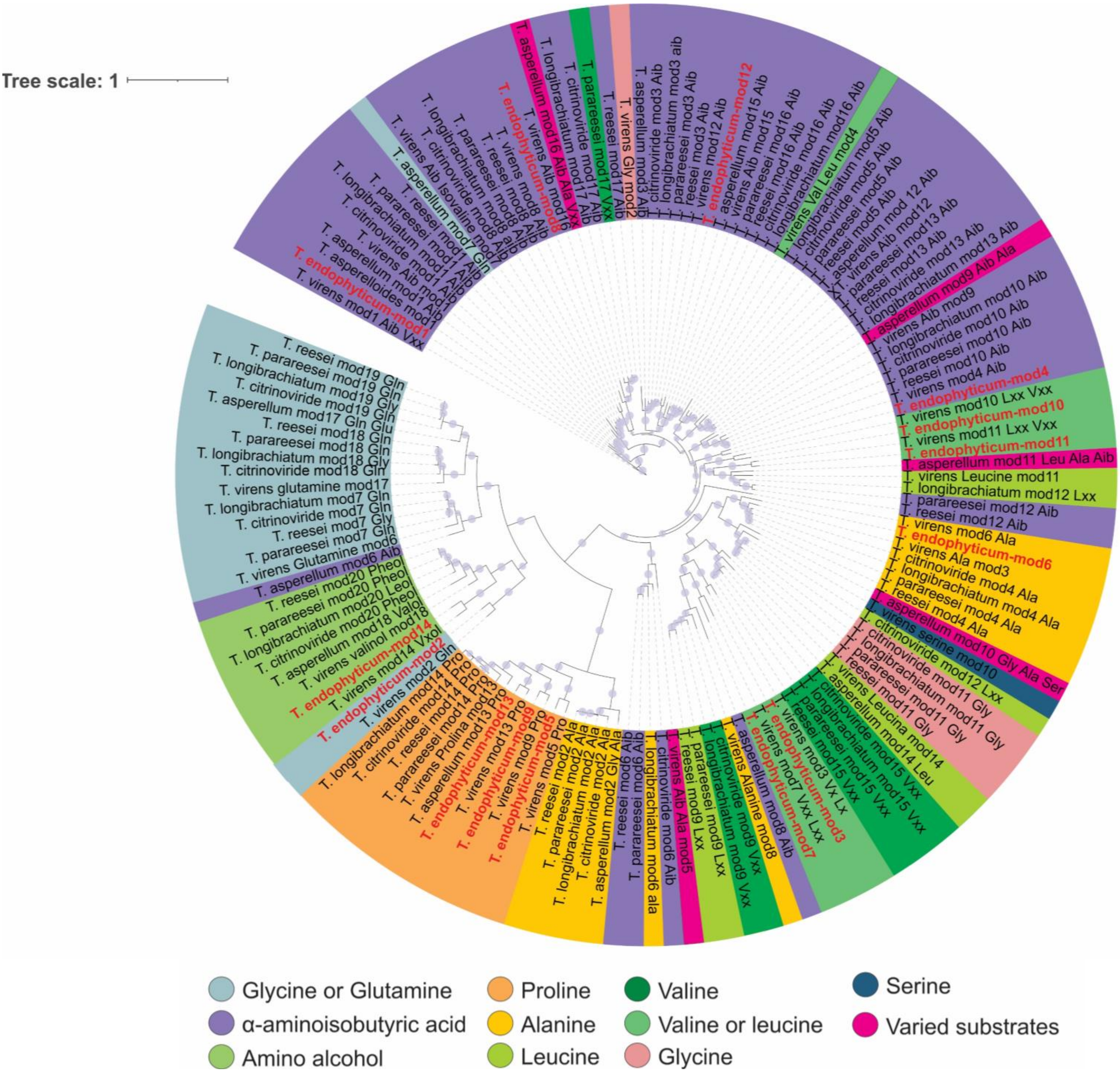

Table S3: LC-MS data of peptaibols annotated in *Trichoderma endophyticum* extract MMSRG85.

Compound	*m/z* [M+H]⁺	Retention time	Molecular formula	Error (ppm)	Íon MS/MS	Sequence
1	1394.8370	37.4	$C_{63}H_{111}N_{17}O_{18}$	-0.07	284.1604, 355.1956, 426.2391, 554.3048, 639.3458, 752.4357, 837.4838, 908.5229, 993.5716, 1078.6187, 1149.6505, 1277.7352, 1394,8387	Ac-Aib-Ala-Aib-Ala-Ala-Gln-Aib-Lxx-Aib-Ala-Aib-Aib-Ala-Gln-Lxxol
2	1394.8322	39.5	$C_{63}H_{111}N_{17}O_{18}$	-3.51	284.1553, 355.1978, 440.2517, 568.3123, 653.3643, 752.4338, 837.4845, 908.5159, 993.5721, 1078.5278, 1149.6594, 1277,7419, 1394.8379	Ac-Aib-Ala-Aib-Ala-Aib-Gln-Aib-Vxx-Aib-Ala-Aib-Aib-Ala-Gln-Lxxol
3	1175.7746	40.4	$C_{58}H_{102}N_{12}O_{13}$	-1.78	355.1983, 468.2845, 553.3363, 961.6092, 1175.4093	Ac-Aib-Gln-Lxx-Vxx-Aib-Pro-Lxx-Lxx-Aib-Pro-Lxxol
4	1444.9157	40.4	$C_{70}H_{121}N_{15}O_{17}$	0.96	369.2154, 454.2676, 822.4721, 1230.7485, 1444.8807	Ac-Aib-Gln-Lxx-Aib-Aib-Ser-Vxx-Pro-Aib-Pro-Lxx-Lxx-Pro-Lxxol
5	1408.8505	40.5	$C_{64}H_{113}N_{17}O_{18}$	1.56	284.1484, 355.2011, 440.2510, 568.3143, 653.3626, 752.4332, 837.4845, 908.5200, 993.5721, 993.5726, 1078.6262, 1163.6783, 1291.7062, 1408.8513	Ac-Aib-Ala-Aib-Ala-Aib-Gln-Aib-Vxx-Aib-Ala-Aib-Aib-Aib-Gln-Lxxol
6	1442.8390	41.1	$C_{67}H_{111}N_{17}O_{18}$	6.72	284.1661, 355.2003, 440.2494, 568.3050, 653.3651, 752.4323, 837.4857, 908.5265, 993.5726, 1078.6259, 1163.6814, 1291,4200, 1442.8397	Ac-Aib-Ala-Aib-Ala-Aib-Gln-Aib-Vxx-Aib-Ala-Aib-Aib-Aib-Gln-Pheol
7	1408.8424	41.6	$C_{64}H_{113}N_{17}O_{18}$	1.06	284.1597, 355.2026, 440.2509, 568.3172, 667.3791, 766.4458, 851.4984, 922.5381, 1007.5884,	Ac-Aib-Ala-Aib-Ala-Aib-Gln-Vxx-Vxx-Aib-Ala-Aib-Aib-Ala-Gln-Lxxol

					1092.6406, 1163.6837, 1291.7368 1408.8523	
8	1422.8657	41.7	**$C_{65}H_{115}N_{17}O_{18}$**	1.89	284.1740, 355.1954, 440.2512, 568.3141, 653.3627, 766.4474, 851.4987, 922.5355, 1007.5898, 1092.6430, 1177.6992, 1305. 7424, 1422.8676	Ac-Aib-Ala-Aib-Ala-Aib-Gln-Aib-Lxx-Aib-Ala-Aib-Aib-Aib-Gln-Lxxol
9	1458.9296	41.8	**$C_{71}H_{123}N_{15}O_{17}$**	0.20	369.2148, 454.2680, 836.4913, 1244.7631, 1458.8755	Ac-Aib-Gln-Lxx-Aib-Pro-Lxx-Aib-Ser-Lxx-Lxx-Pro-Aib-Pro-Lxxol
10	1456.8501	42.3	**$C_{68}H_{113}N_{17}O_{18}$**	1.78	284.1656, 355.1958, 440.2491, 568.3138, 656.3638, 766.4485, 922.5378, 1007.5888, 1092.6419, 1177.6948, 1306.7673, 1456.8544	Ac-Aib-Ala-Aib-Ala-Aib-Gln-Aib-Lxx-Aib-Ala-Aib-Aib-Aib-Gln-Pheol
11	1409.8345	42.6	**$C_{64}H_{112}N_{16}O_{19}$**	1.56	284.1545, 355.1992, 440.2523, 568.3125, 653.3638, 752.4288, 908.5219, 993.5745, 1078.6253, 1163.6845, 1292.7164, 1305.7491, 1409.8410	Ac-Aib-Ala-Aib-Ala-Aib-Gln-Aib-Vxx-Aib-Ala-Aib-Aib-Aib-Glu-Lxxol
12	1422.8665	43.3	**$C_{65}H_{115}N_{17}O_{18}$**	1.33	284.1616, 355.1993, 454.2678, 582.3273, 667,3808 780.4649, 865.5158, 936.5531, 1021.6070, 1106.6585, 1177.6998,1422.8688	Ac-Aib-Ala-Aib-Ala-Vxx-Gln-Aib-Lxx-Aib-Ala-Aib-Aib-Ala-Gln-Lxxol
13	1428. 9224	43.4	**$C_{70}H_{121}N_{15}O_{16}$**	2.09	369.2143, 454.2683, 806.4772, 1214.7550, 1428.9136	Ac-Aib-Gln-Lxx-Aib-Pro-Ala-Vxx-Aib-Pro-Lxx-Lxx-Aib-Pro-Lxxol
14	1436.8805	43.7	**$C_{66}H_{117}N_{17}O_{18}$**	2.43	284.1582, 355.1988, 454.2674, 582.3278, 681.3946, 794.4769, 879.5296, 950.5671, 1035.6188, 1120.6722, 1191.7089, 1319.7460 1436.8801	Ac-Aib-Ala-Aib-Ala-Vxx-Gln-Vxx-Lxx-Aib-Ala-Aib-Aib-Ala-Gln-Lxxol
15	1423.8642	44.0	**$C_{65}H_{114}N_{16}O_{19}$**	8.28	284.1576, 355.1982, 440.2513,	Ac-Aib-Ala-Aib-Ala-Aib-Gln-Vxx-Vxx-Aib-Ala-Aib-Aib-Aib-Glu-Lxxol

					568.3123, 667.3783, 766.4446, 851.4976, 922.5364, 1007.5905, 1092.6452, 1177.6919, 1306.7379, 1423.8536	
16	1175. 7683	44.0	**$C_{58}H_{102}N_{12}O_{13}$**	7.14	369.2131, 468.2825, 553.3329, 961.6094, 1175.7225	Ac-Aib-Gln-Vxx-Lxx-Aib-Pro-Lxx-Lxx-Aib-Pro-Lxxol
17	1470.8589	44.2	**$C_{69}H_{115}N_{17}O_{18}$**	2.37	284.1722, 355.1994, 440.2502, 568.3014, 667.3768, 780.4610, 865.5139, 936.5531, 1021.6047, 1106.6617, 1191.7090, 1470.8703	Ac-Aib-Ala-Aib-Ala-Vxx-Gln-Vxx-Vxx-Aib-Ala-Aib-Aib-Aib-Gln-Pheol
18	1456.8505	44.7	**$C_{68}H_{113}N_{17}O_{18}$**	-1.51	284.1617, 355.1963, 454.2669, 582.3243, 681.3923, 780.4607, 865.5146, 936.5494, 1021.6053, 1106.6567, 1177.6776, 1319.7197, 1456.8458	Ac-Aib-Ala-Aib-Ala-Vxx-Gln-Vxx-Vxx-Aib-Ala-Aib-Aib-Ala-Gln-Pheol
19	1423.8525	44.8	**$C_{65}H_{114}N_{16}O_{19}$**	0.07	284.1668, 355.1995,454.2680, 582.3255, 681.3951, 780.4641, 865.5164, 936.5510, 1021.6060, 1106.6563, 1177.6946, 1423.8664	Ac-Aib-Ala-Aib-Ala-Vxx-Gln-Vxx-Vxx-Aib-Ala-Aib-Aib-Ala-Glu-Lxxol
20	1470.8673	45.5	**$C_{69}H_{115}N_{17}O_{18}$**	0.74	284.1606, 355.1975, 454.2676, 582.3241, 681.3934, 780.4607, 865.5146, 936.5503, 1021.6009, 1106.6558, 1191.7141, 1319.7568, 1470.8667	Ac-Aib-Ala-Aib-Ala-Aib-Gln-Vxx-Lxx-Aib-Ala-Aib-Aib-Aib-Gln-Pheol
21	1189.7795	45.5	**$C_{61}H_{110}N_{14}O_{16}$**	8.74	369.2137, 482.2979, 567.3479, 779.4644, 975.6276, 1189.7397	Ac-Aib-Gln-Lxx-Lxx-Aib-Vxx-Lxx-Pro-Vxx-Pro-Lxxol
~~23~~ 22	1436.8827	45.6	**$C_{66}H_{117}N_{17}O_{18}$**	0.90	284.1643, 355.1993, 440.2498, 568.3101, 667.3788, 780.4624, 865.5143, 936.5512, 1021.6029, 106.6537, 1191.7119,	Ac-Aib-Ala-Aib-Ala-Aib-Gln-Vxx-Lxx-Aib-Ala-Aib-Aib-Aib-Gln-Lxxol

					1319.7707, 1436.8820	
~~22~~23	1450.8959	46.5	$C_{67}H_{119}N_{17}O_{18}$	-1.51	284.1587, 355.1978, 454.2669, 582.3289, 681.3947, 794.4773, 950.5690, 1035.6197, 1120.6743, 1205.7256, 1333.7759, 1450.8943	Ac-Aib-Ala-Aib-Ala-Vxx-Gln-Vxx-Lxx-Aib-Ala-Aib-Aib-Aib-Gln-Lxxol
24	1437.8672	46.6	$C_{66}H_{116}N_{16}O_{19}$	0.55	284.1626, 355.1987, 454.2655, 582.3260, 667.3785, 780.4620, 865.5145, 936.5504, 1021.6044,1106.6561, 1191.7109, 1320.7566, 1437.8666	Ac-Aib-Ala-Aib-Ala-Vxx-Gln-Aib-Lxx-Aib-Ala-Aib-Aib-Aib-Glu-Lxxol
25	1484.8862	46.9	$C_{70}H_{117}N_{17}O_{18}$	1.48	284.1626, 355.1955,454.2649, 582.3268, 681.3925, 794.4753, 879.5292, 950.5676, 1035.6190, 1120.6728, 1205.7273, 1333.7802, 1484.8852	Ac-Aib-Ala-Aib-Ala-Vxx-Gln-Vxx-Lxx-Aib-Ala-Aib-Aib-Aib-Gln-Pheol
26	1471.8369	46.9	$C_{69}H_{114}N_{16}O_{19}$	3.05	284.1522, 355.1979, 454.2668, 582.3385, 667.3755, 780.4692, 865.5148, 936.5466, 1021.6040, 1106.6511, 1191.7112, 1320.7664, 1471.8489	Ac-Aib-Ala-Aib-Ala-Vxx-Gln-Aib-Lxx-Aib-Ala-Aib-Aib-Aib-Glu-Pheol
27	1485.8639	46.9	$C_{70}H_{116}N_{16}O_{19}$	-2.75	284.1630, 355.1991, 454.2704, 582.3242, 681.3919, 794.4776, 879.5294, 950.5666, 1035.6229, 1120.6690, 1205.7367, 1334.7902, 1485.8585	Ac-Aib-Ala-Aib-Ala-Vxx-Gln-Vxx-Vxx-Aib-Ala-Aib-Aib-Aib-Gln-Pheol

Figure S3: Total ion chromatogram of the annotated compounds. Numbers indicate compounds annotated in ascending order of retention time (RT).

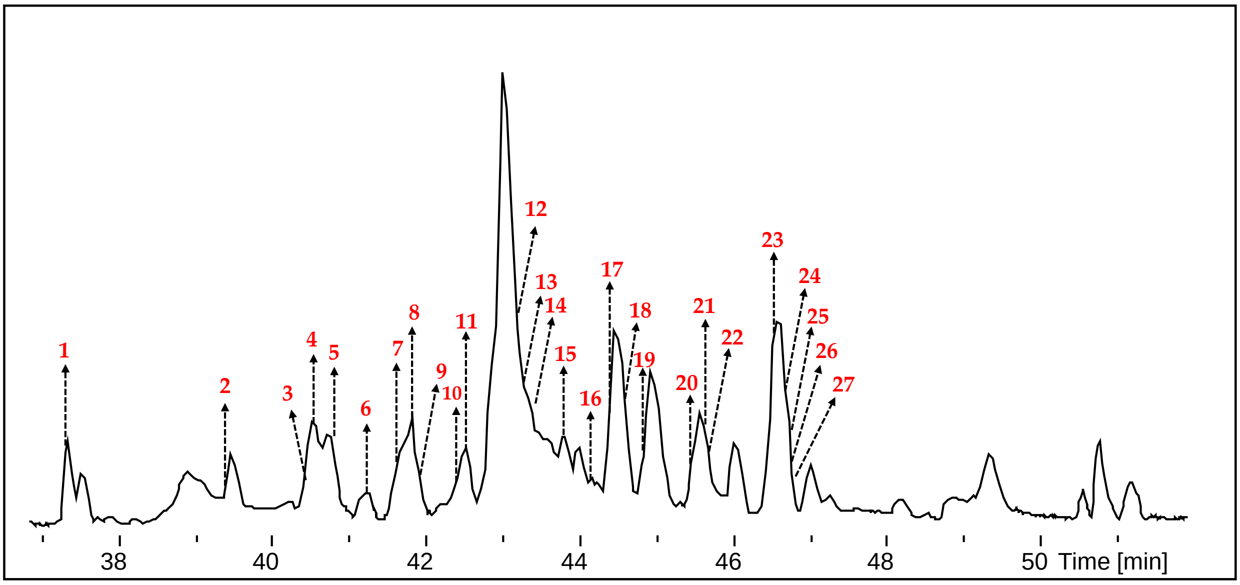

Figure S4. Mirror match of peptaibol harzianin HC XIII (black color spectrum), annotated by means of similarity with the MS/MS spectrum present in the GNPS spectral library (green color spectrum).

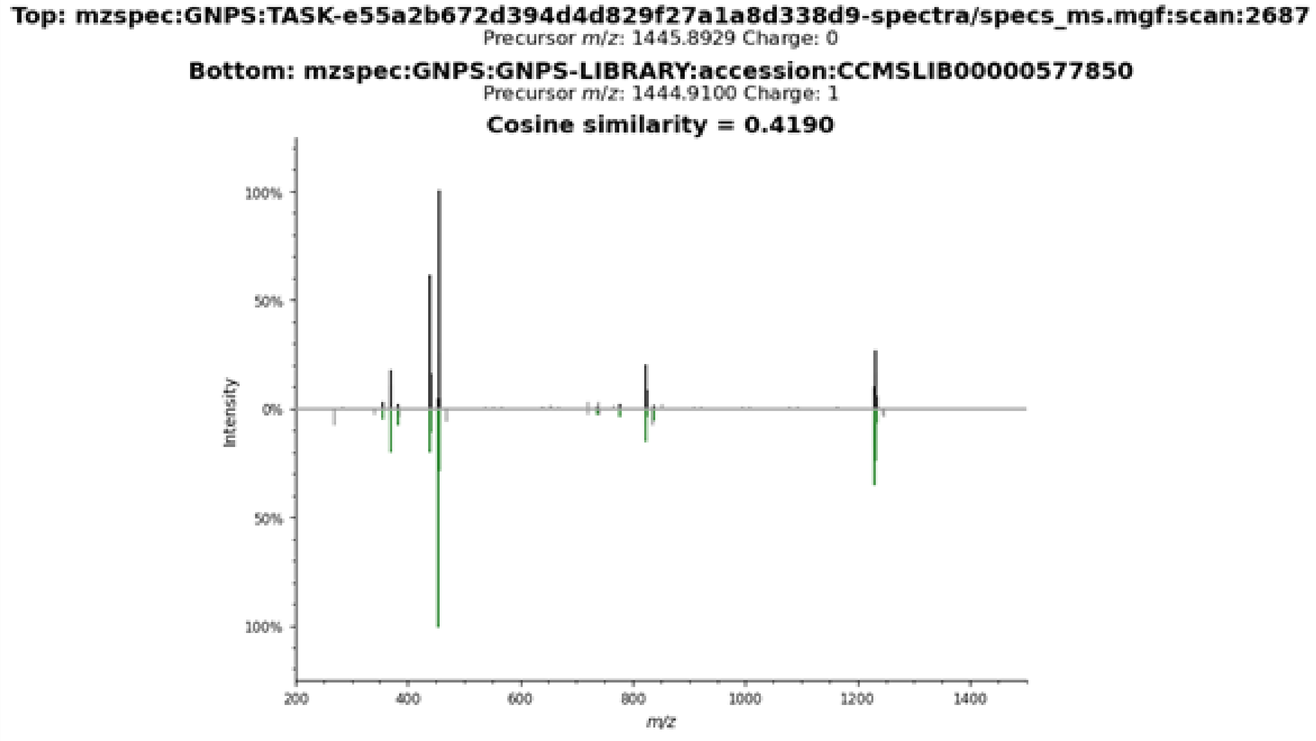

Figure S5: MS (**A**) and MS/MS (**B**) spectra of compound 4.

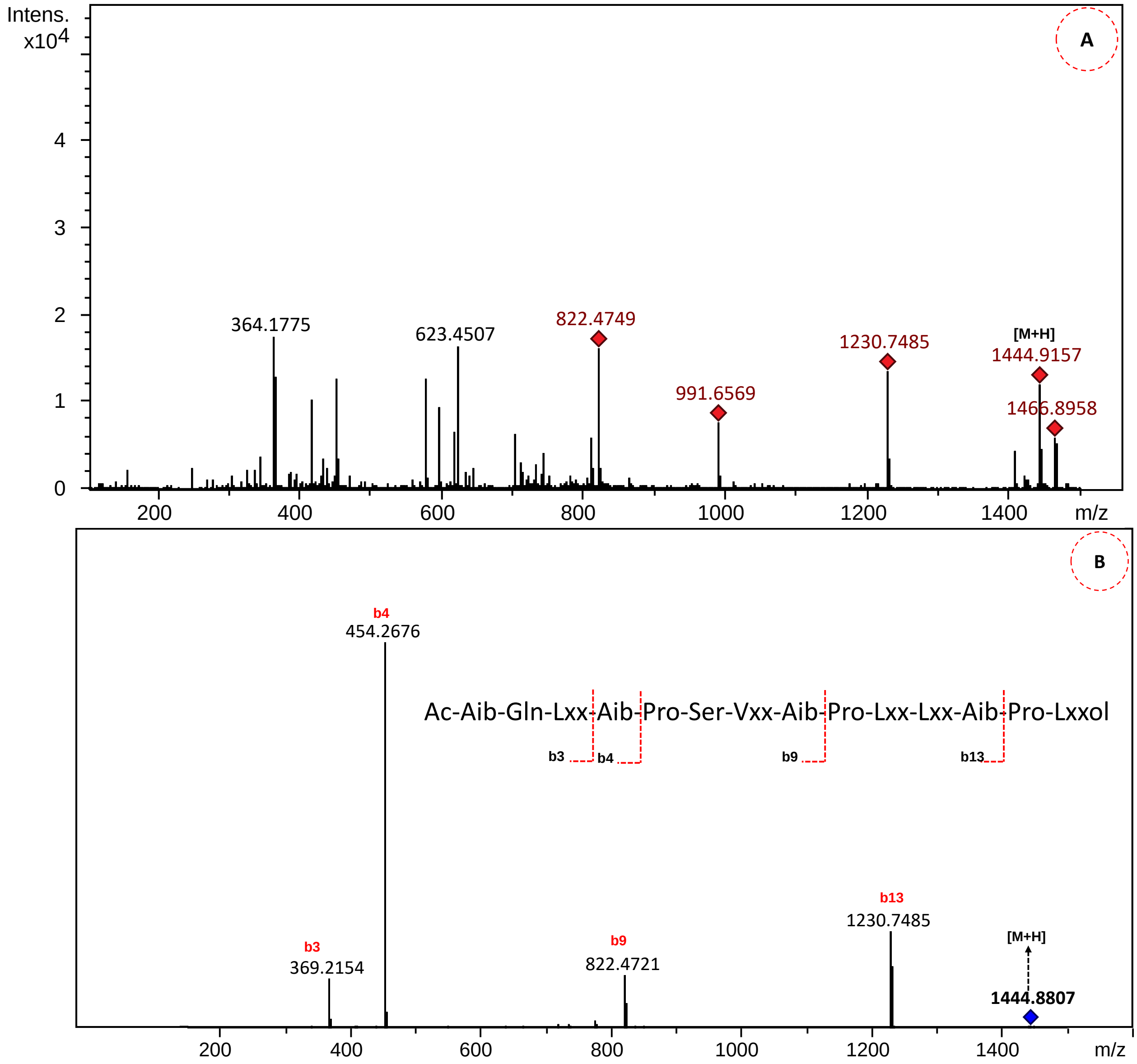

Figure S6: MS (**A**) and MS/MS (**B**) spectra of compound 13.

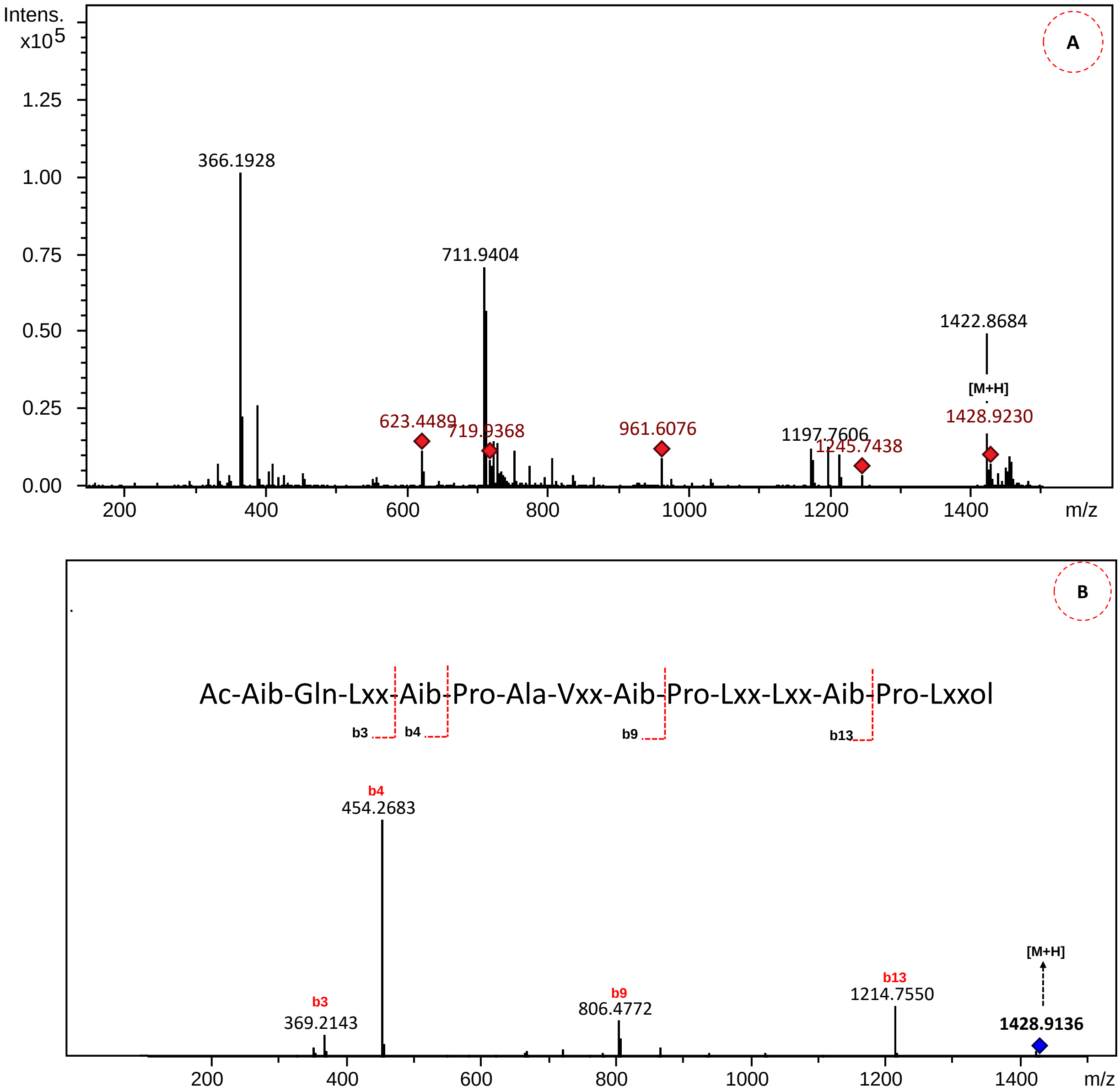

Figure S7: MS (**A**) and MS/MS (**B**) spectra of compound 9.

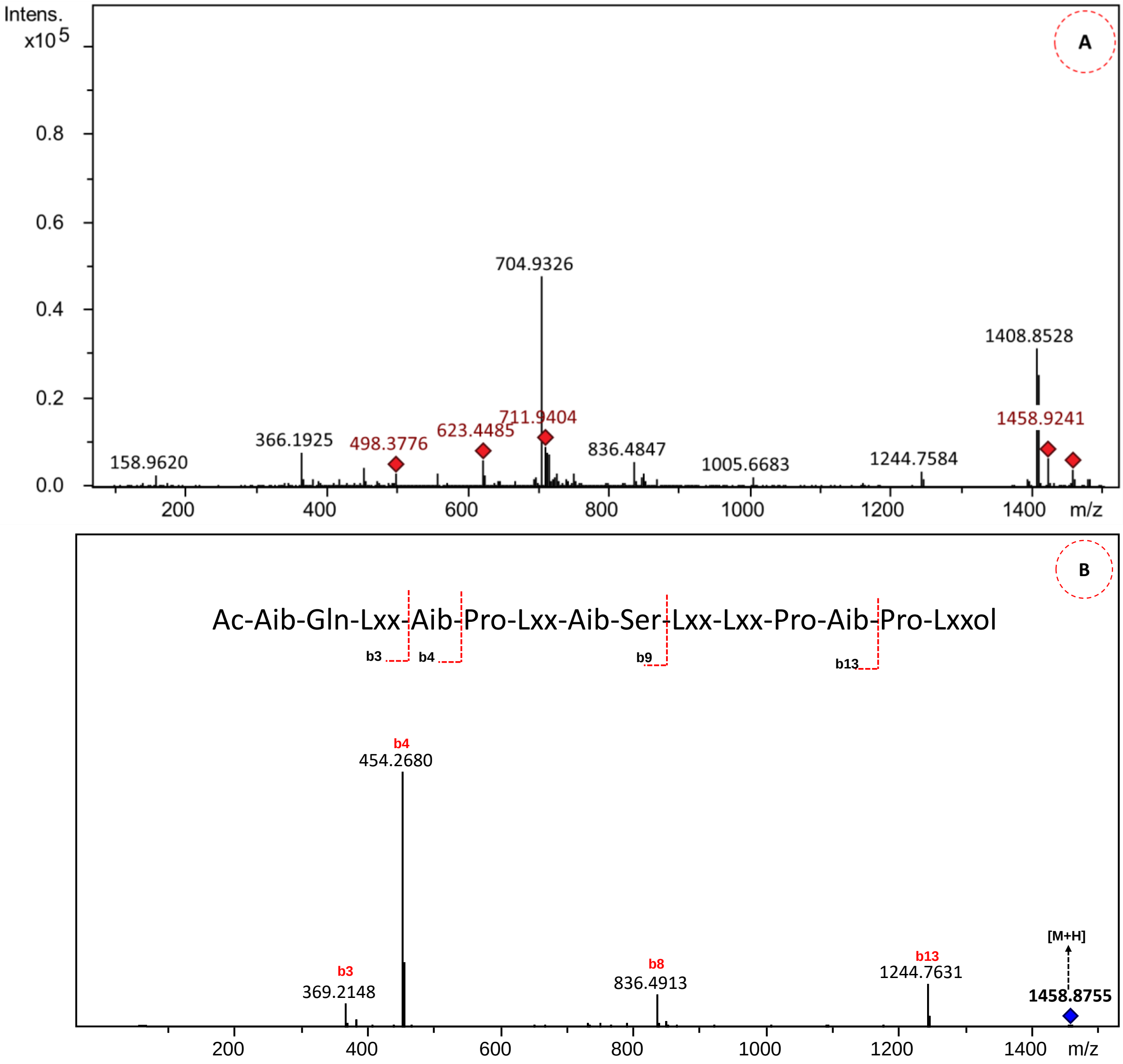

Figure S8: MS (**A**) and MS/MS (**B**) spectra of compound 3.

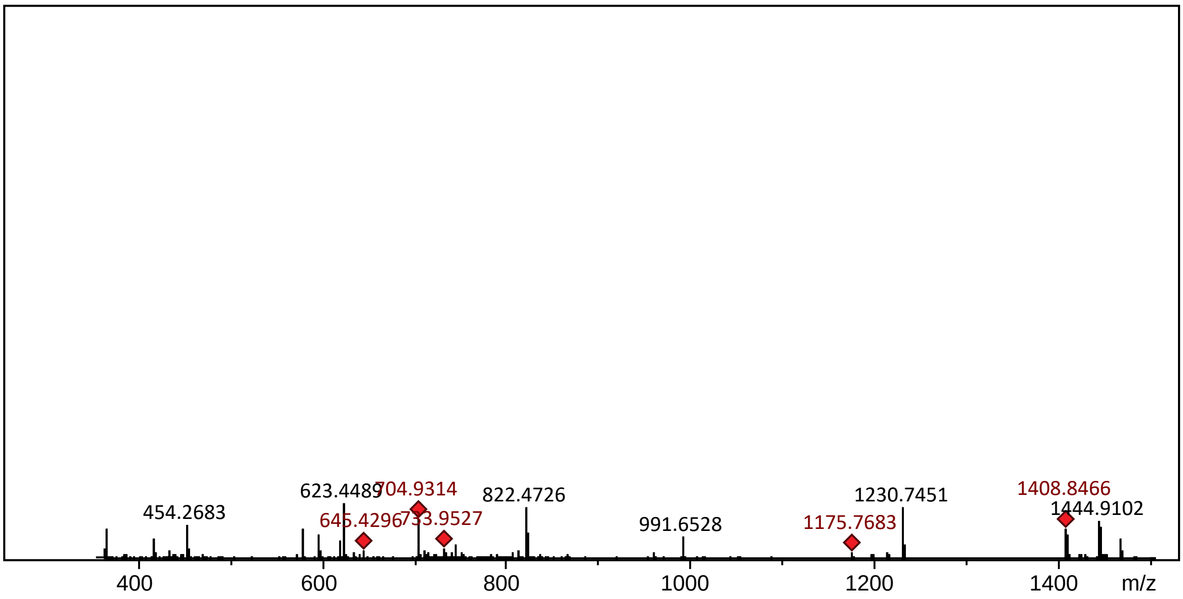

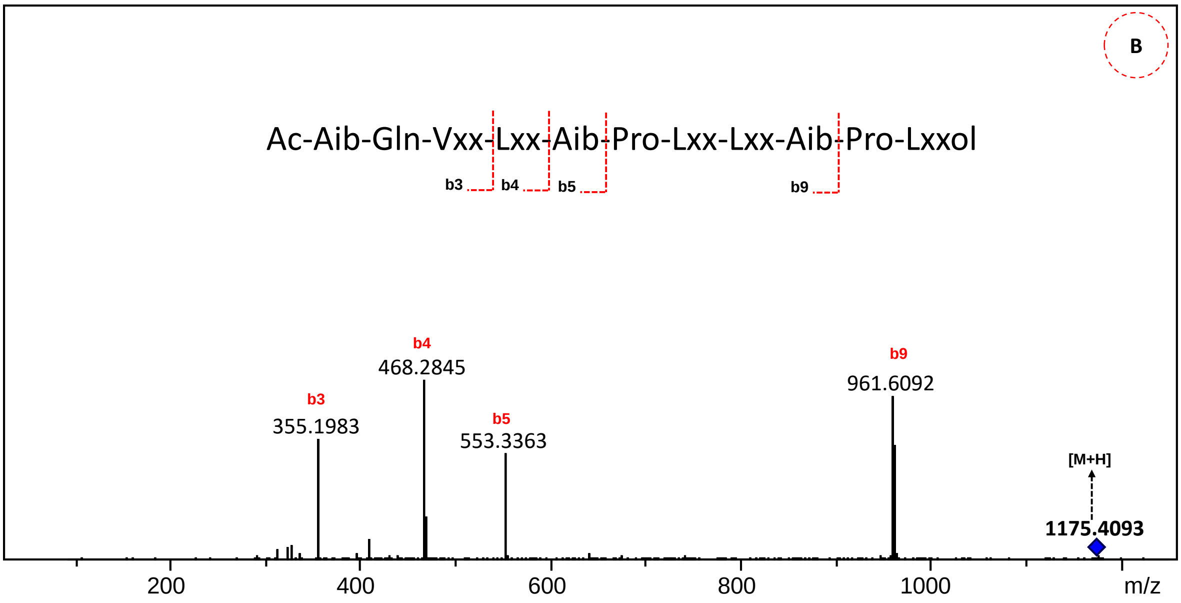

Figure S9: MS (**A**) and MS/MS (**B**) spectra of compound 16.

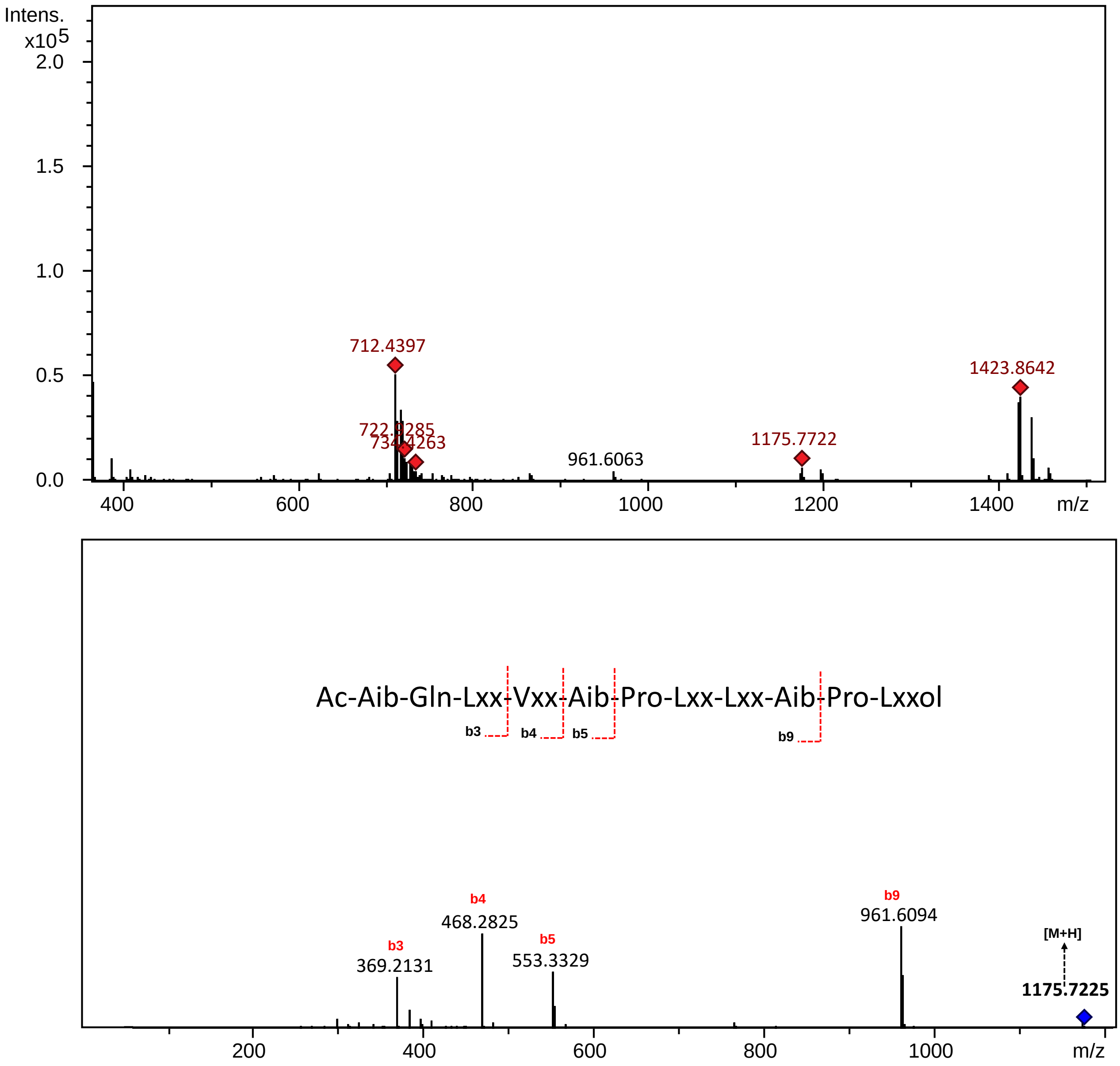

Figure S10: MS (**A**) and MS/MS (**B**) spectra of compound 21.

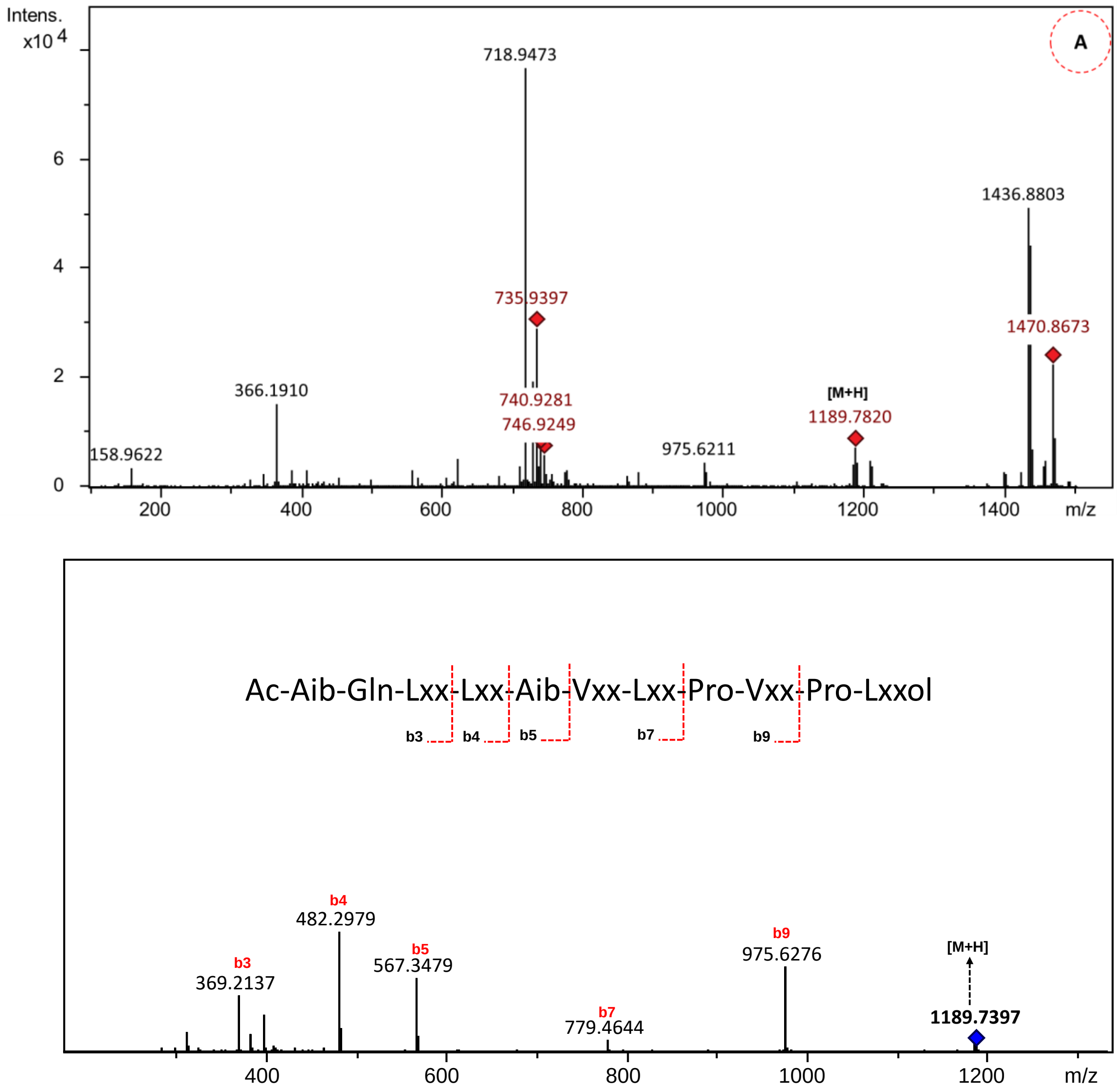

Figure S11: MS (**A**) and MS/MS (**B**) spectra of **endophytin A1.**

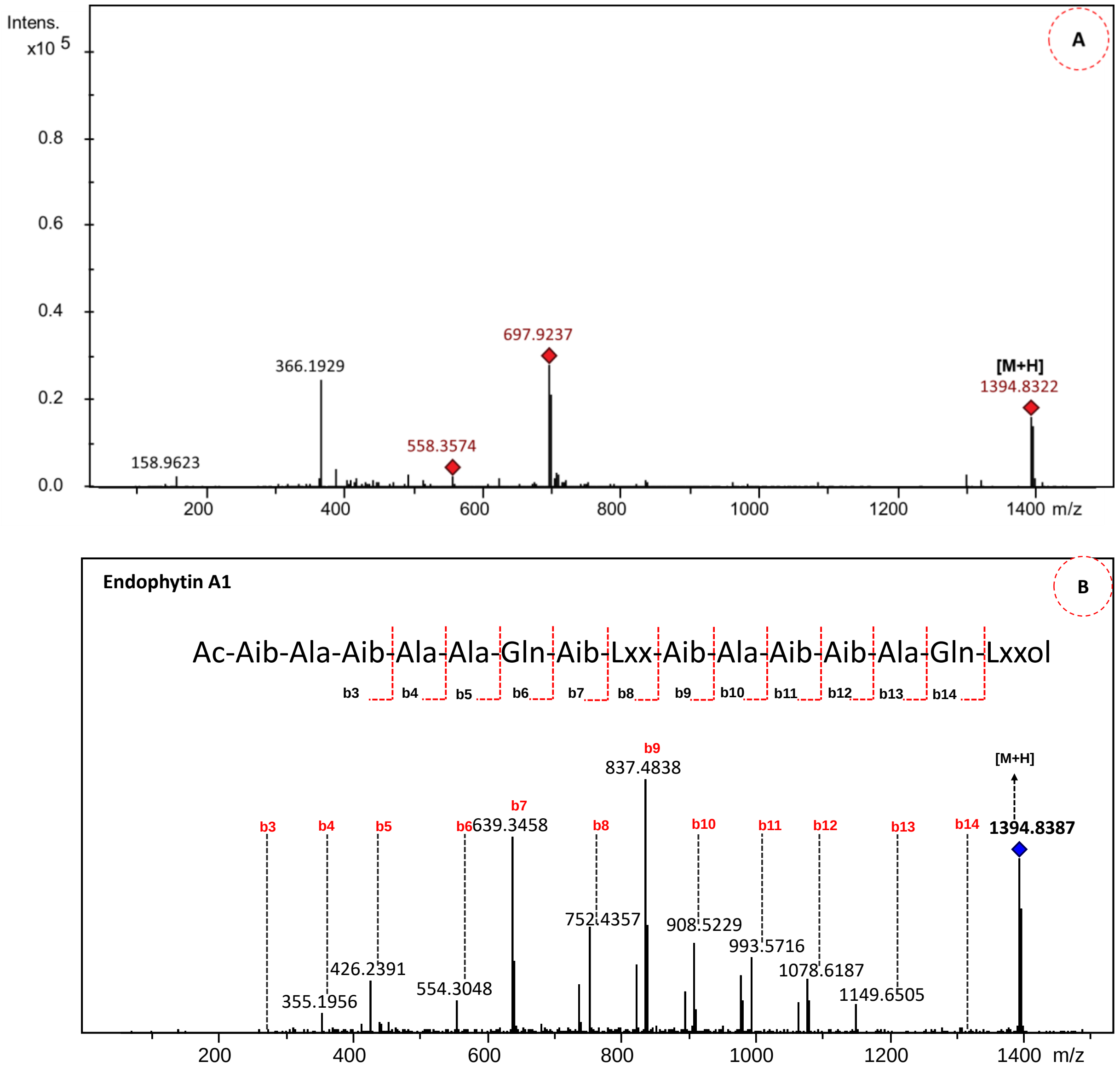

Figure S12: MS (**A**) and MS/MS (**B**) spectra of **endophytin A3.**

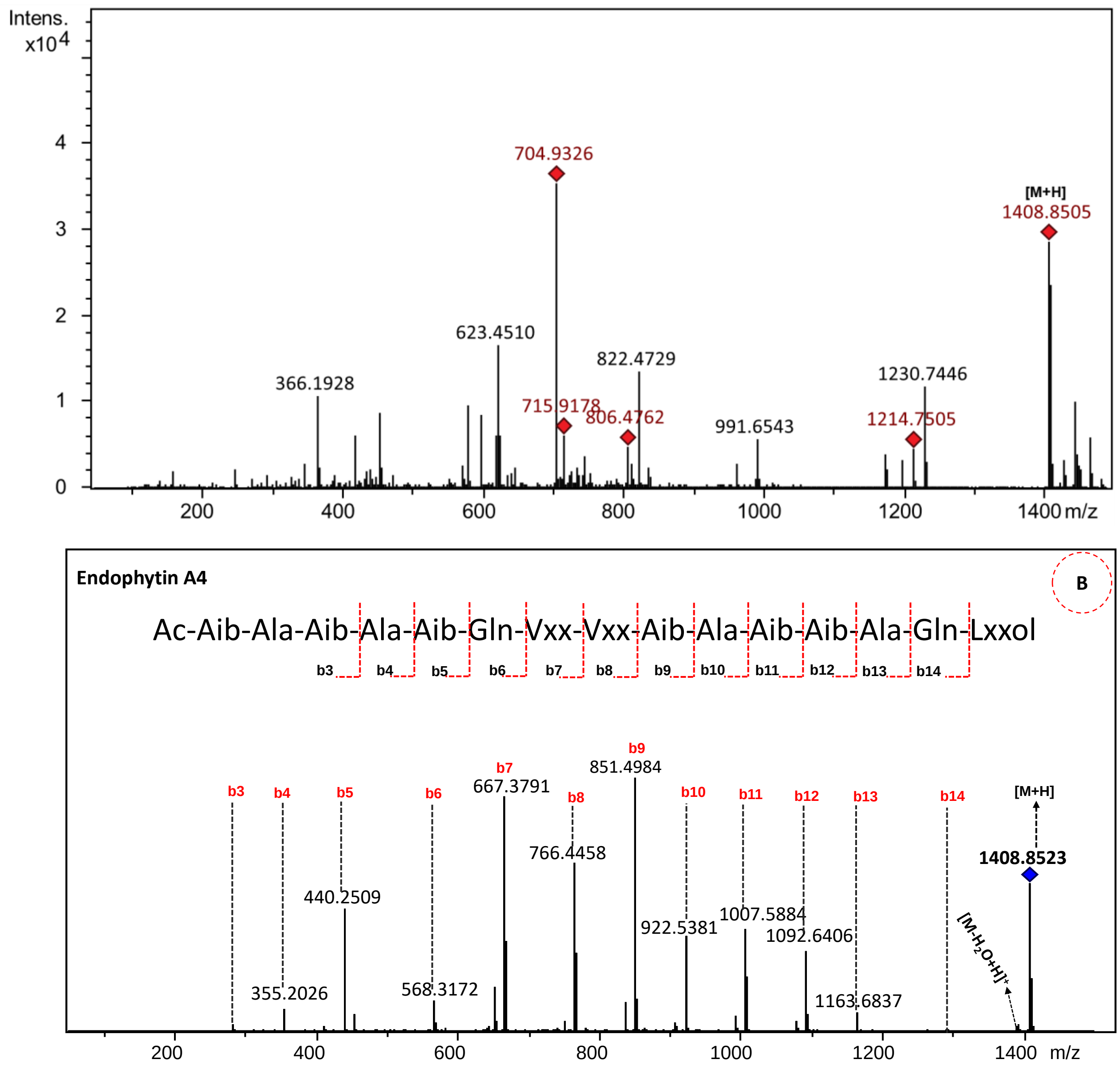

Figure S13: MS (**A**) and MS/MS (**B**) spectra of **endophytin A4.**

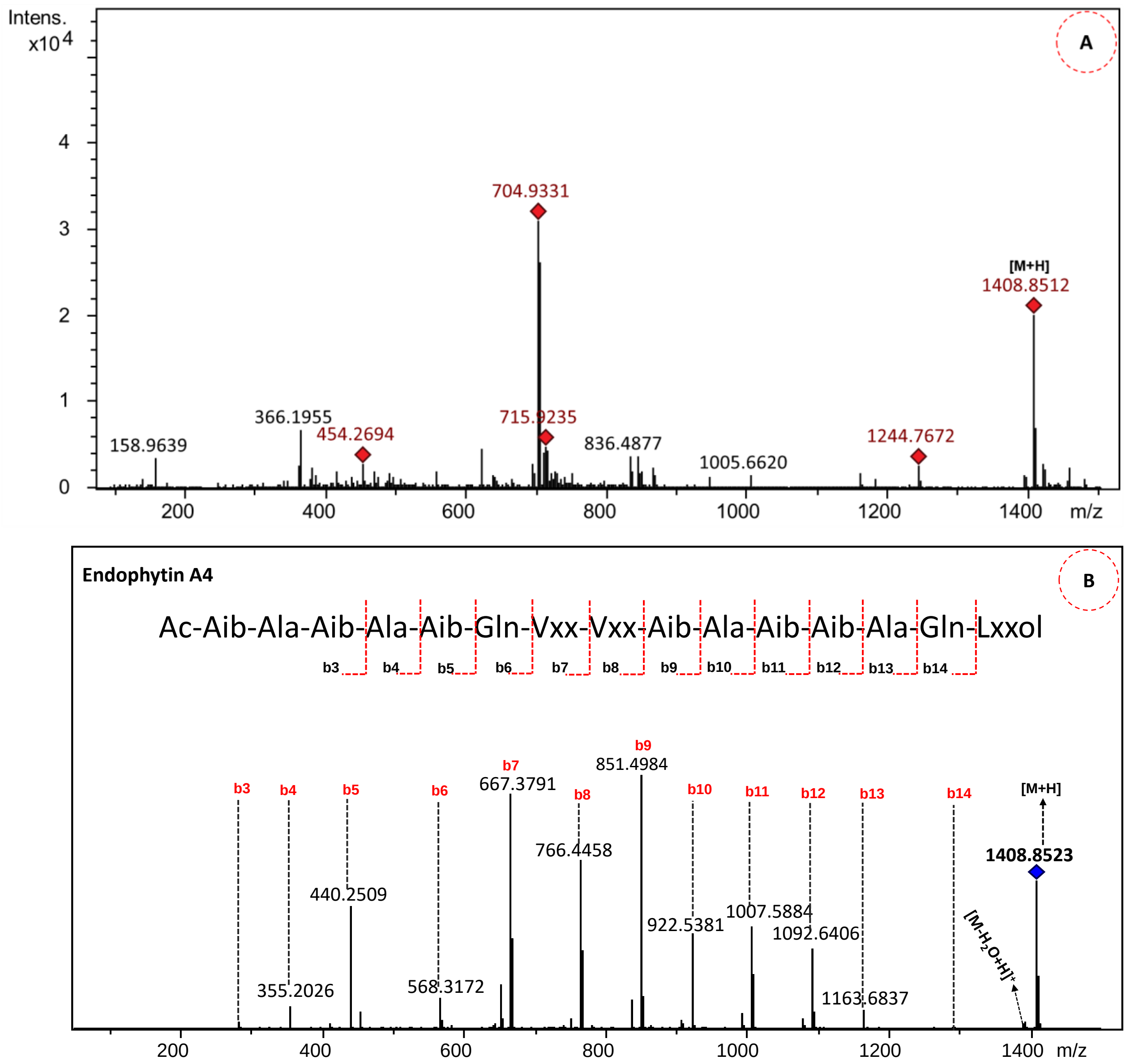

Figure S14: MS (**A**) and MS/MS (**B**) spectra of **endophytin A5.**

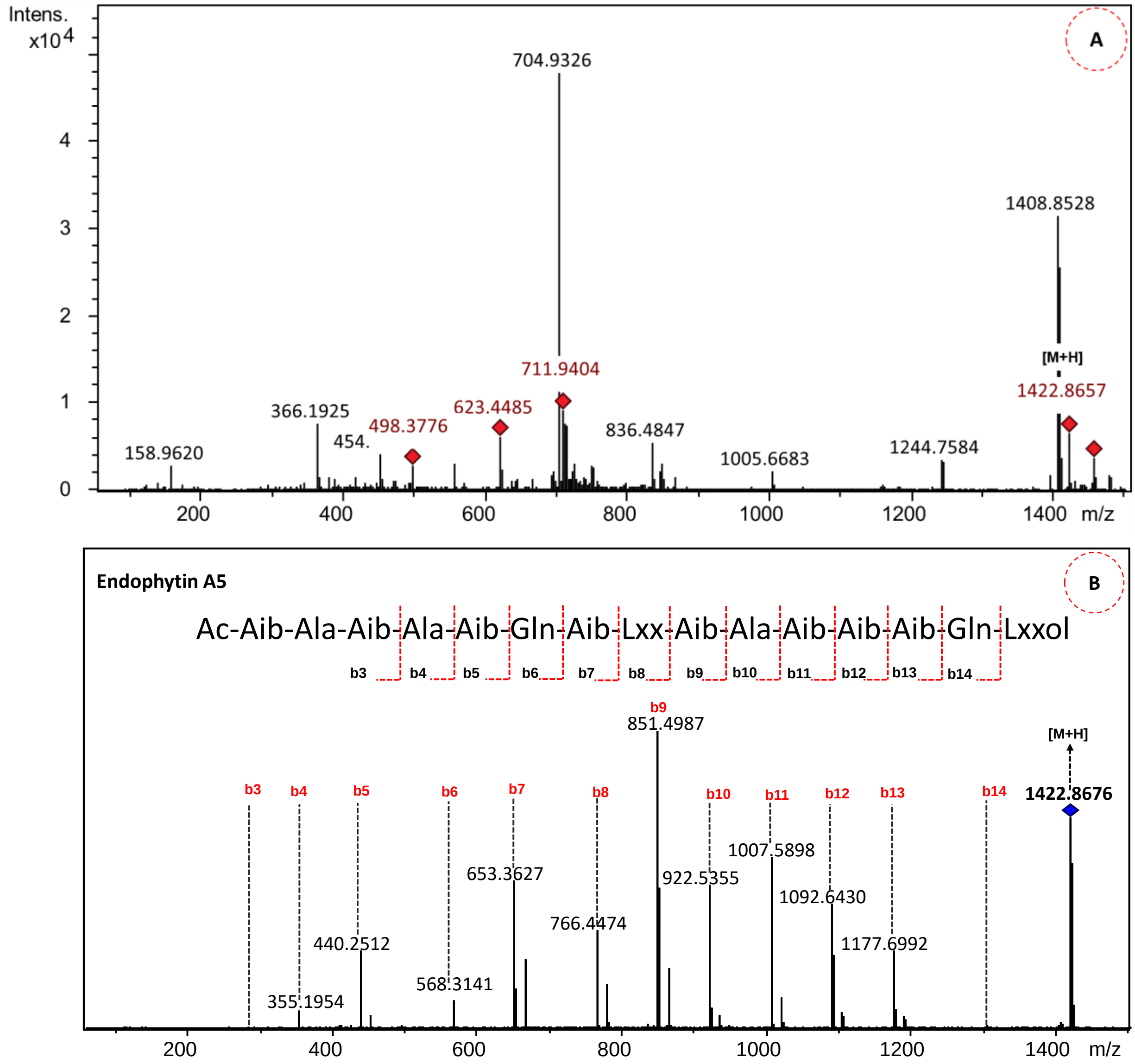

Figure S15: MS (**A**) and MS/MS (**B**) spectra of **endophytin A6.**

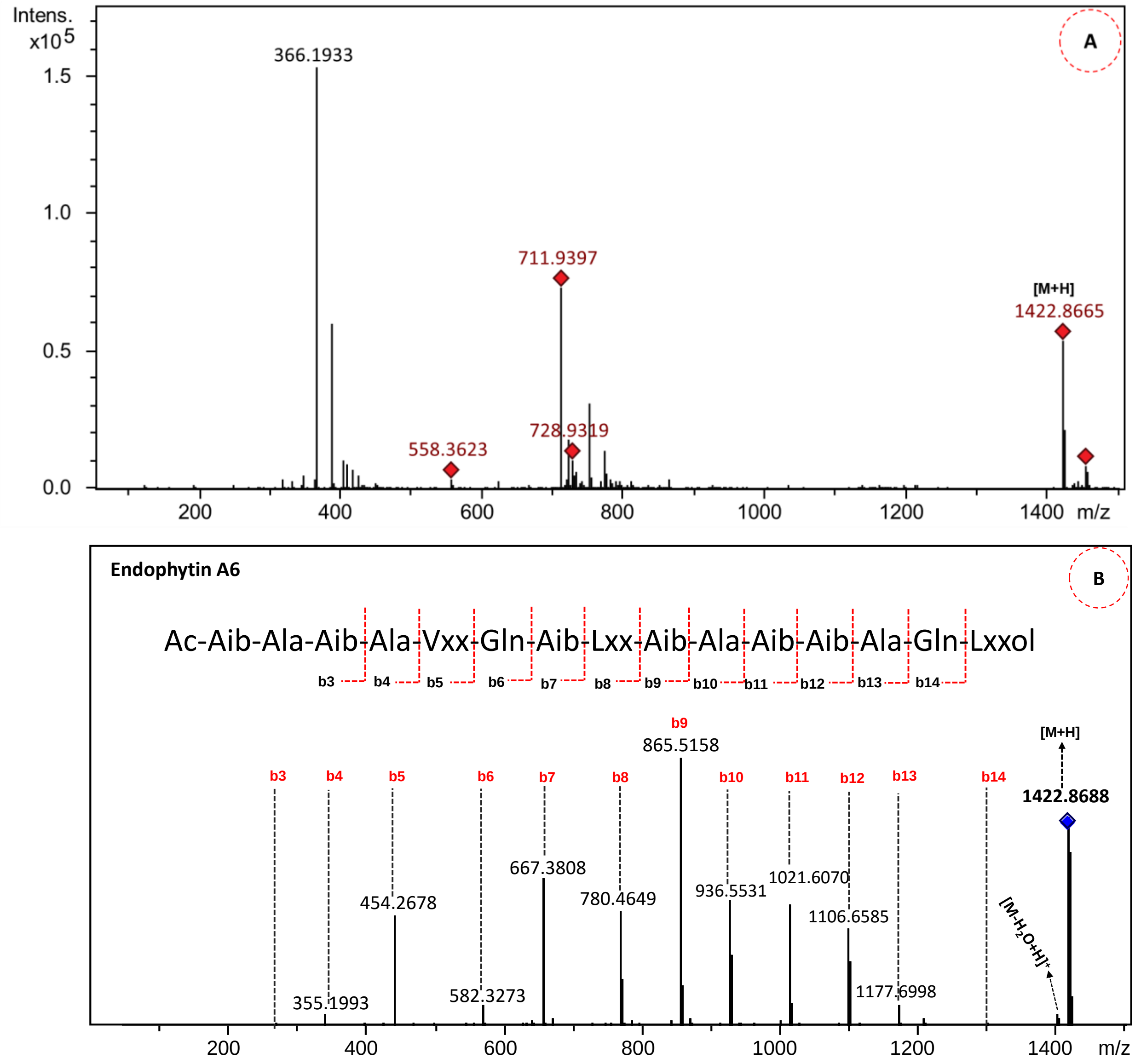

Figure S16: MS (**A**) and MS/MS (**B**) spectra of **endophytin A7.**

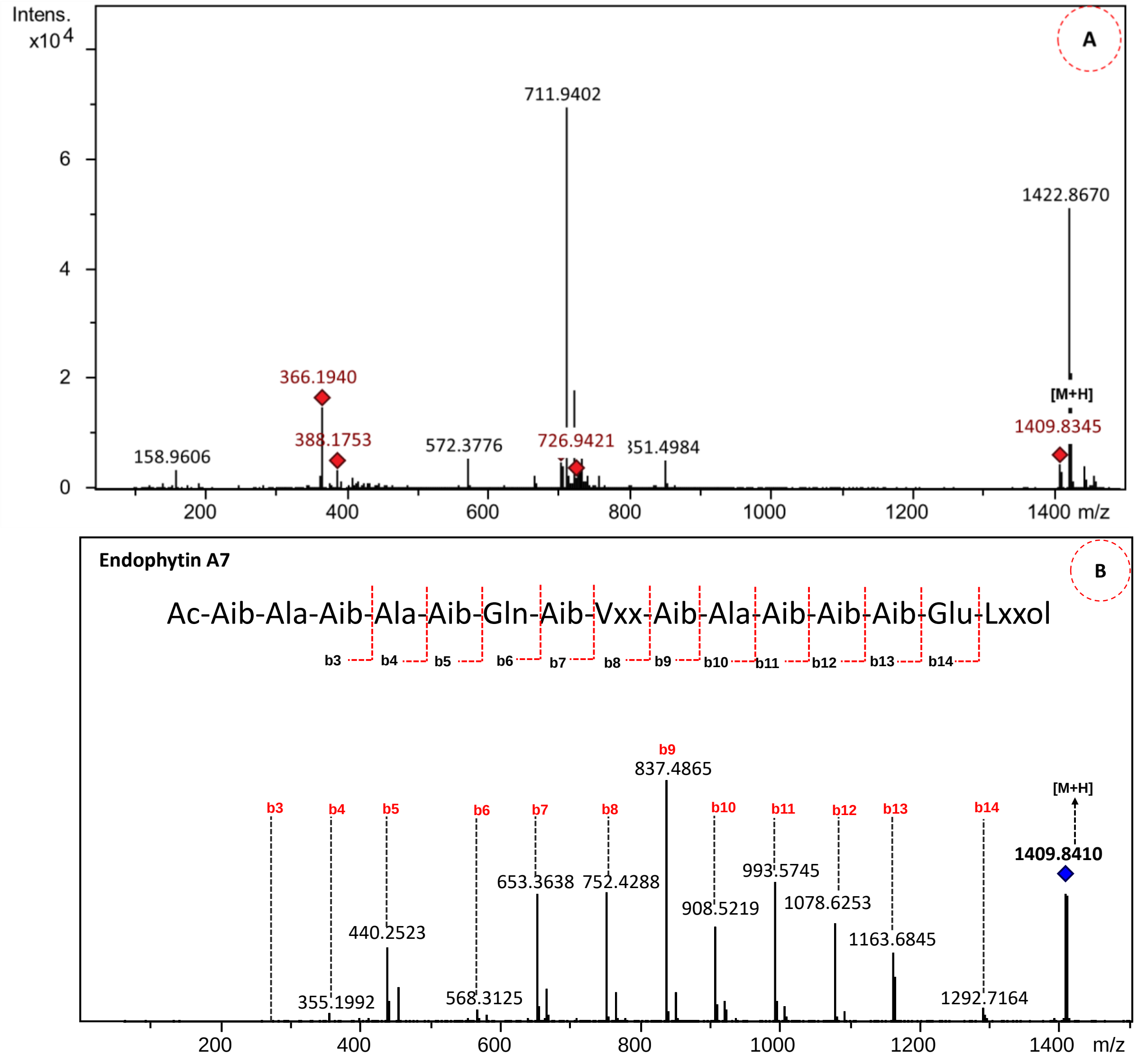

Figure S17: MS (**A**) and MS/MS (**B**) spectra of **endophytin A8.**

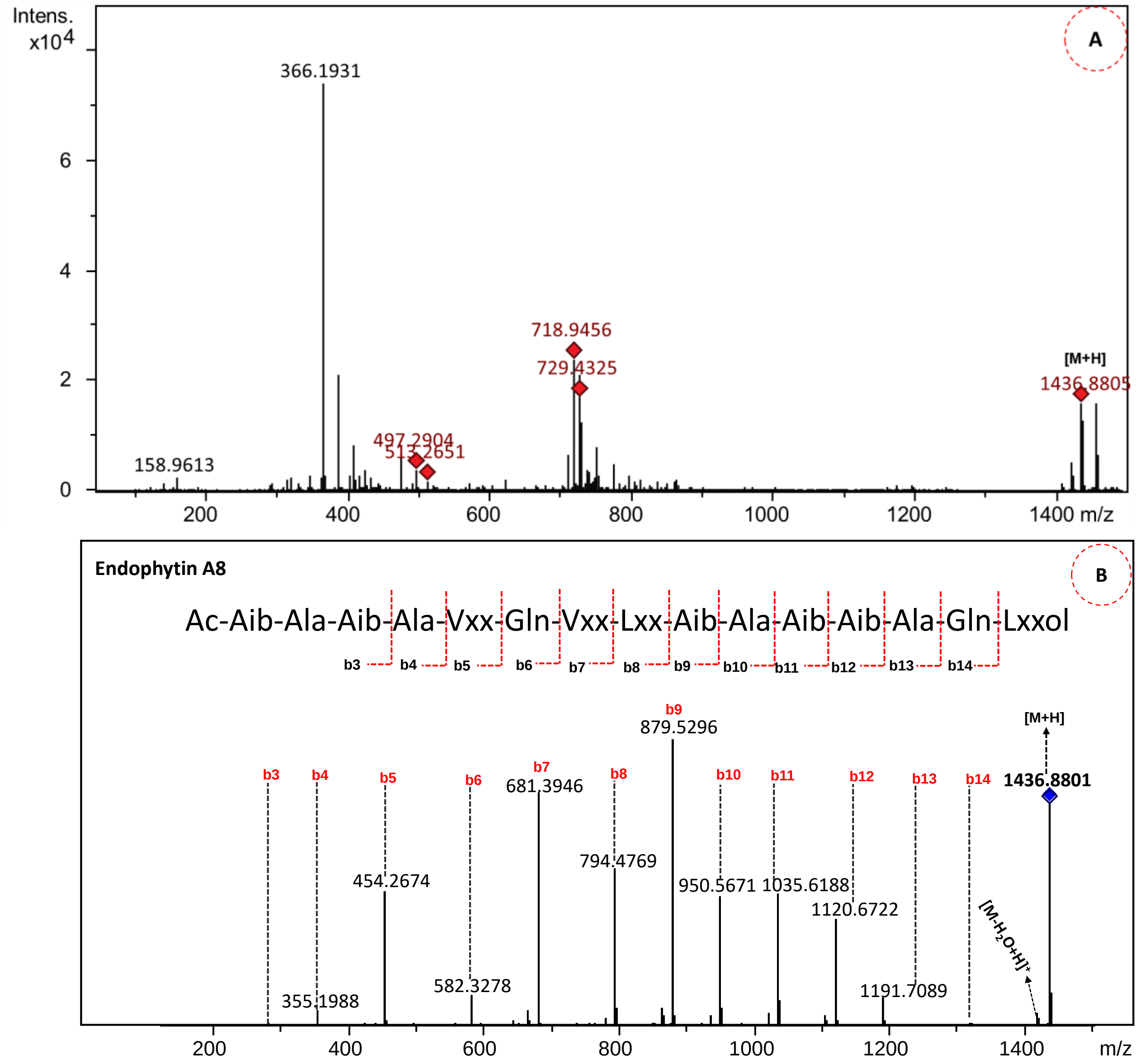

Figure S18: MS (**A**) and MS/MS (**B**) spectra of **endophytin A9.**

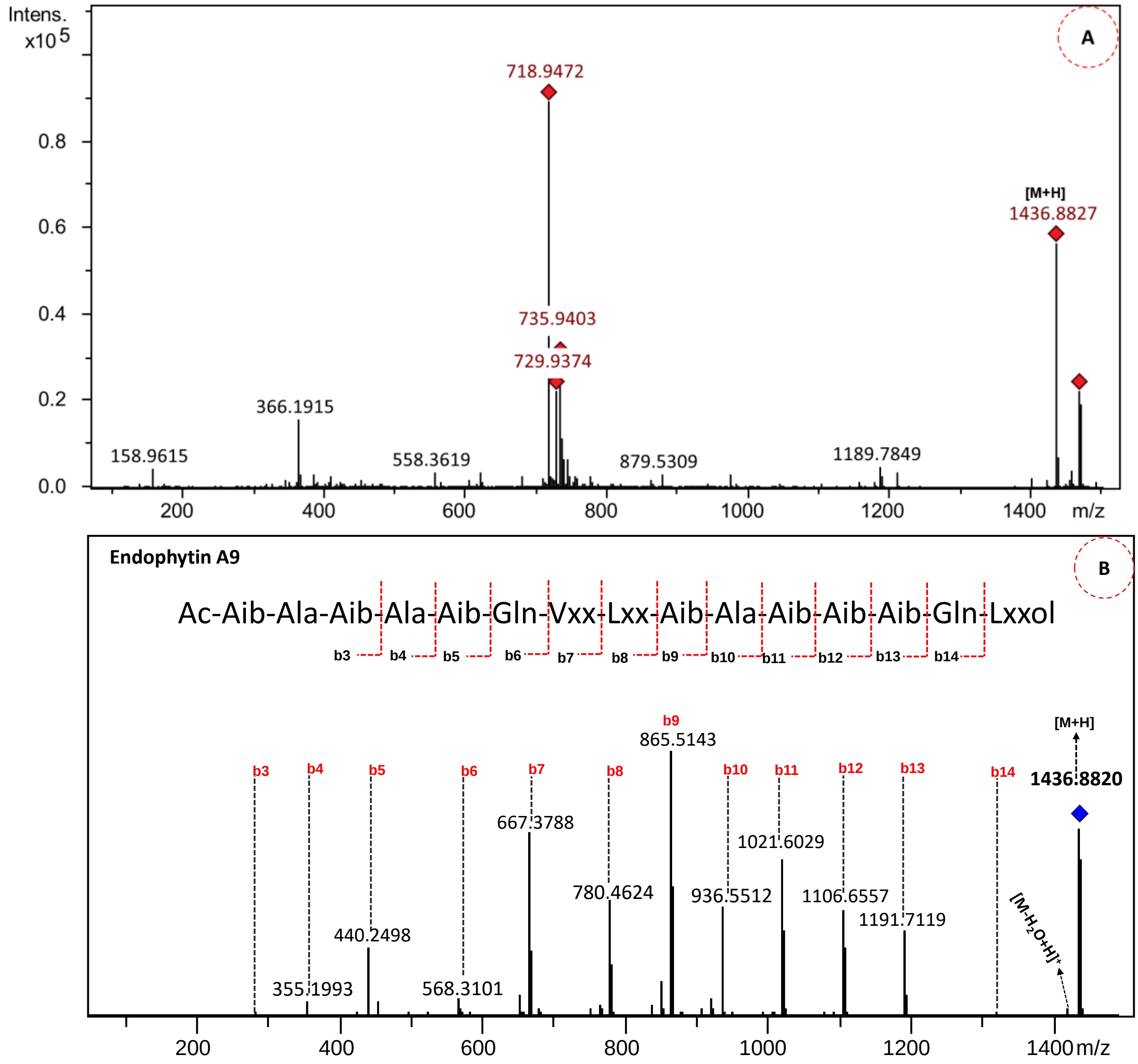

Figure S19: MS (**A**) and MS/MS (**B**) spectra of **endophytin A10.**

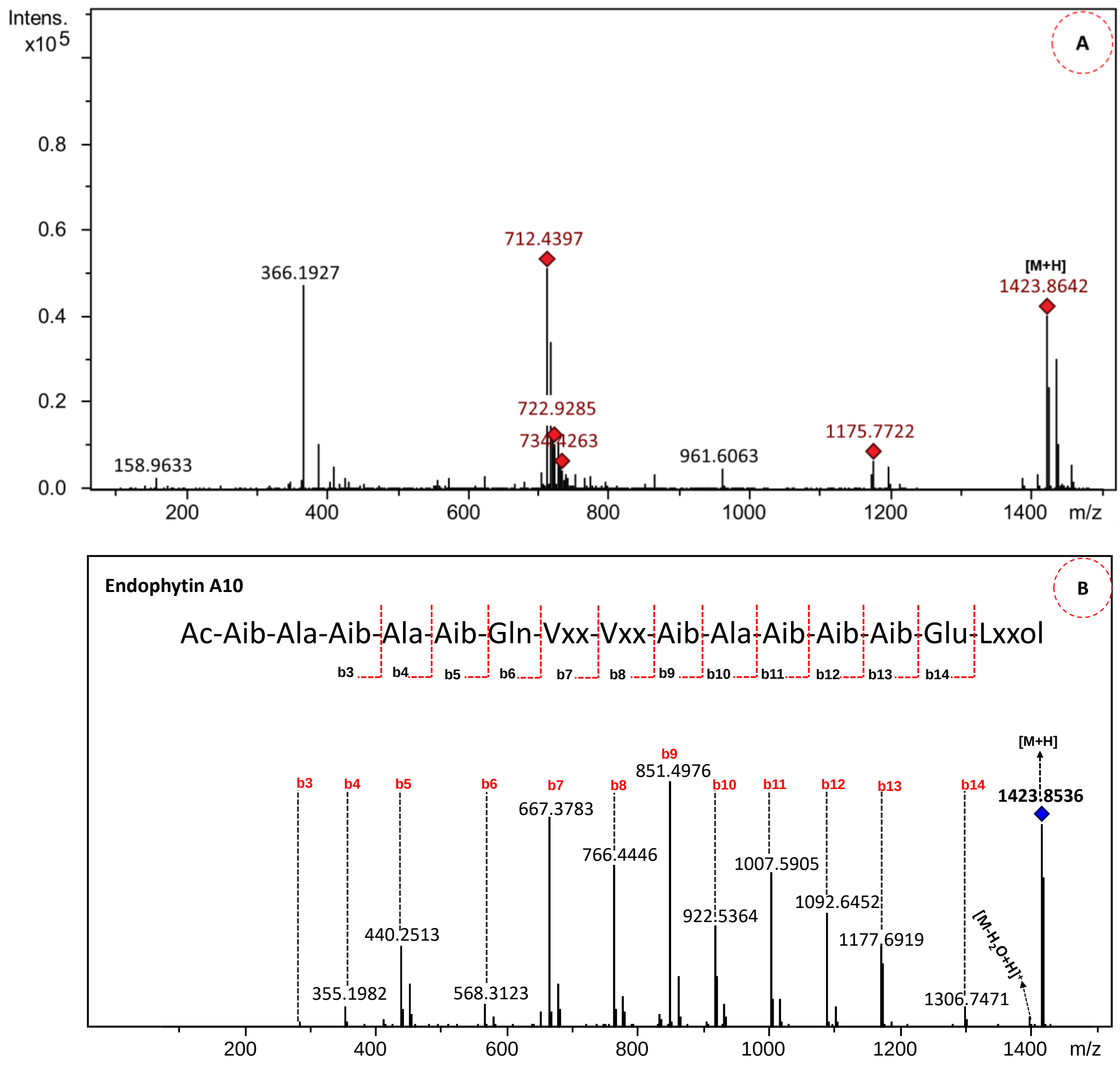

Figure S20: MS (**A**) and MS/MS (**B**) spectra of **endophytin A11.**

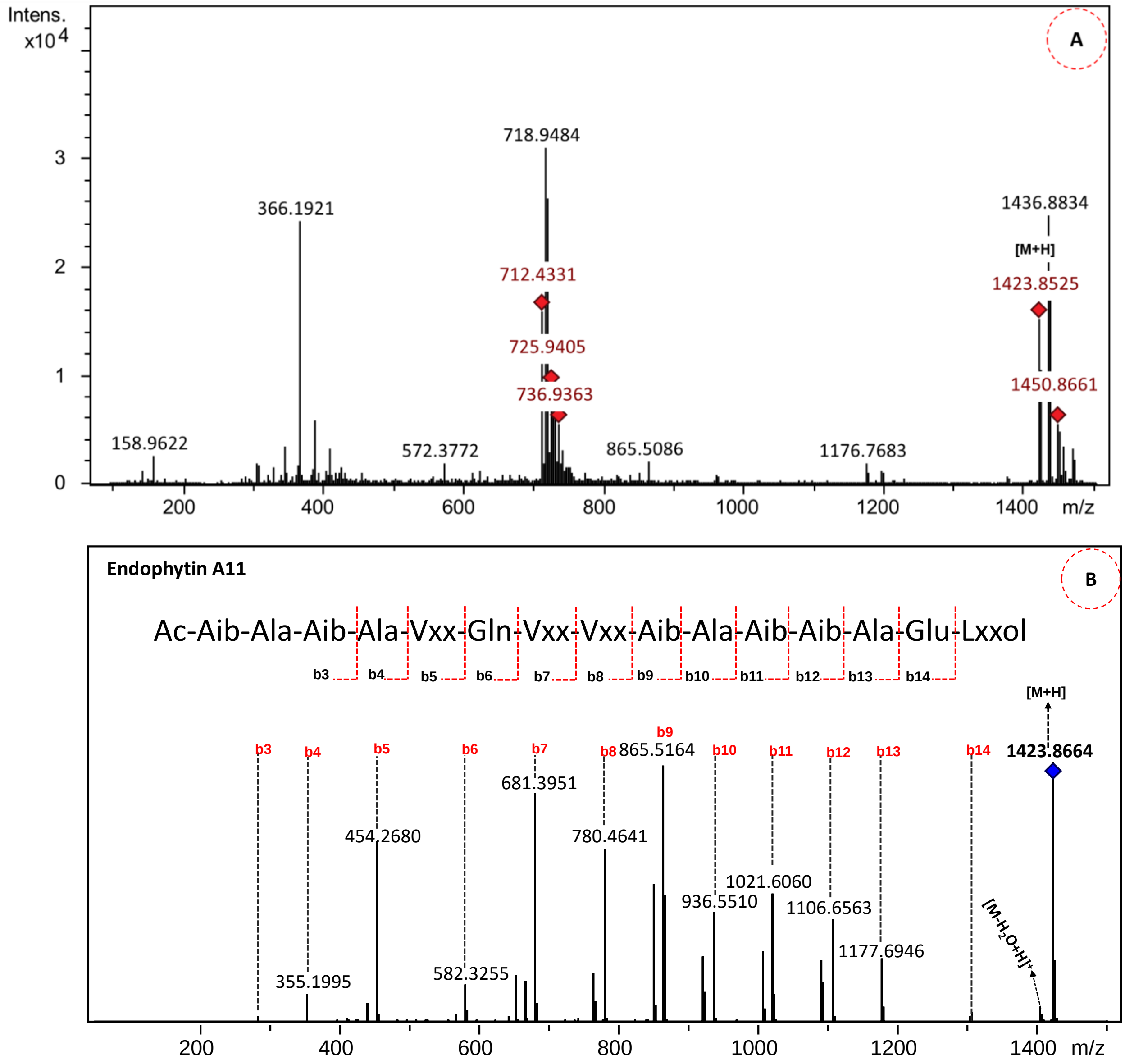

Figure S21: MS (**A**) and MS/MS (**B**) spectra of **endophytin A12.**

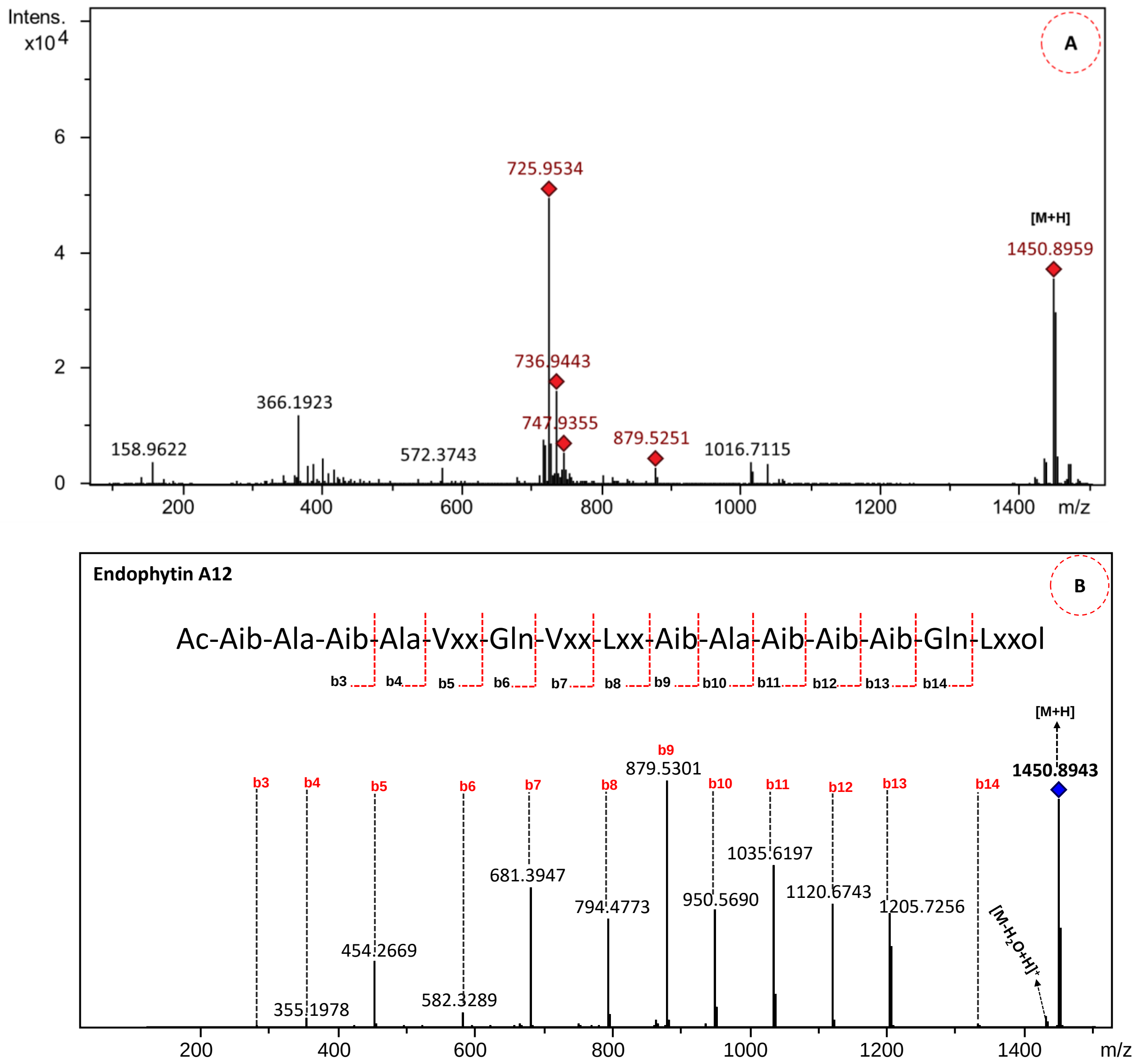

Figure S22: MS (**A**) and MS/MS (**B**) spectra of **endophytin A13.**

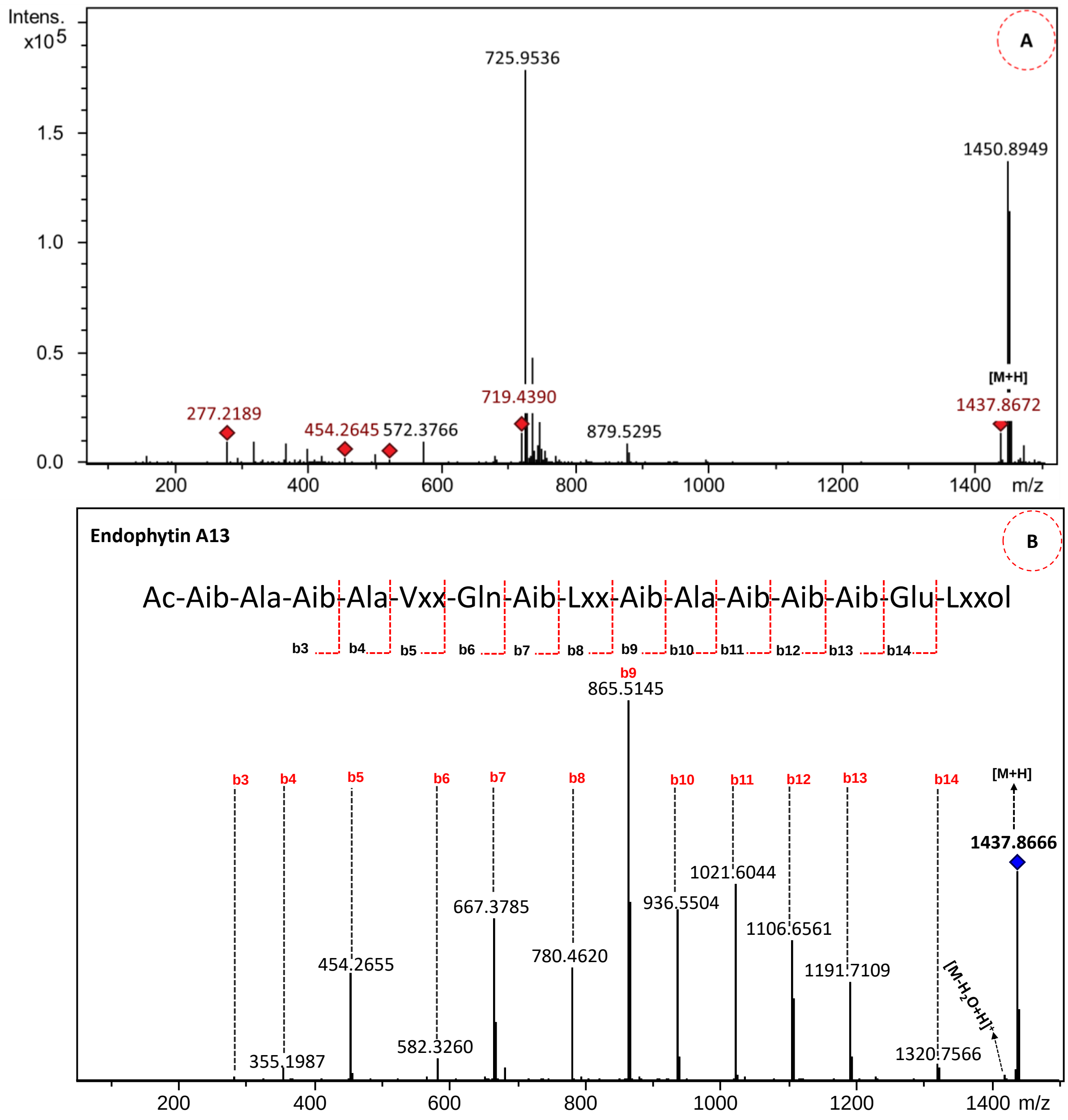

Figure S23: MS (**A**) and MS/MS (**B**) spectra of **endophytin B1.**

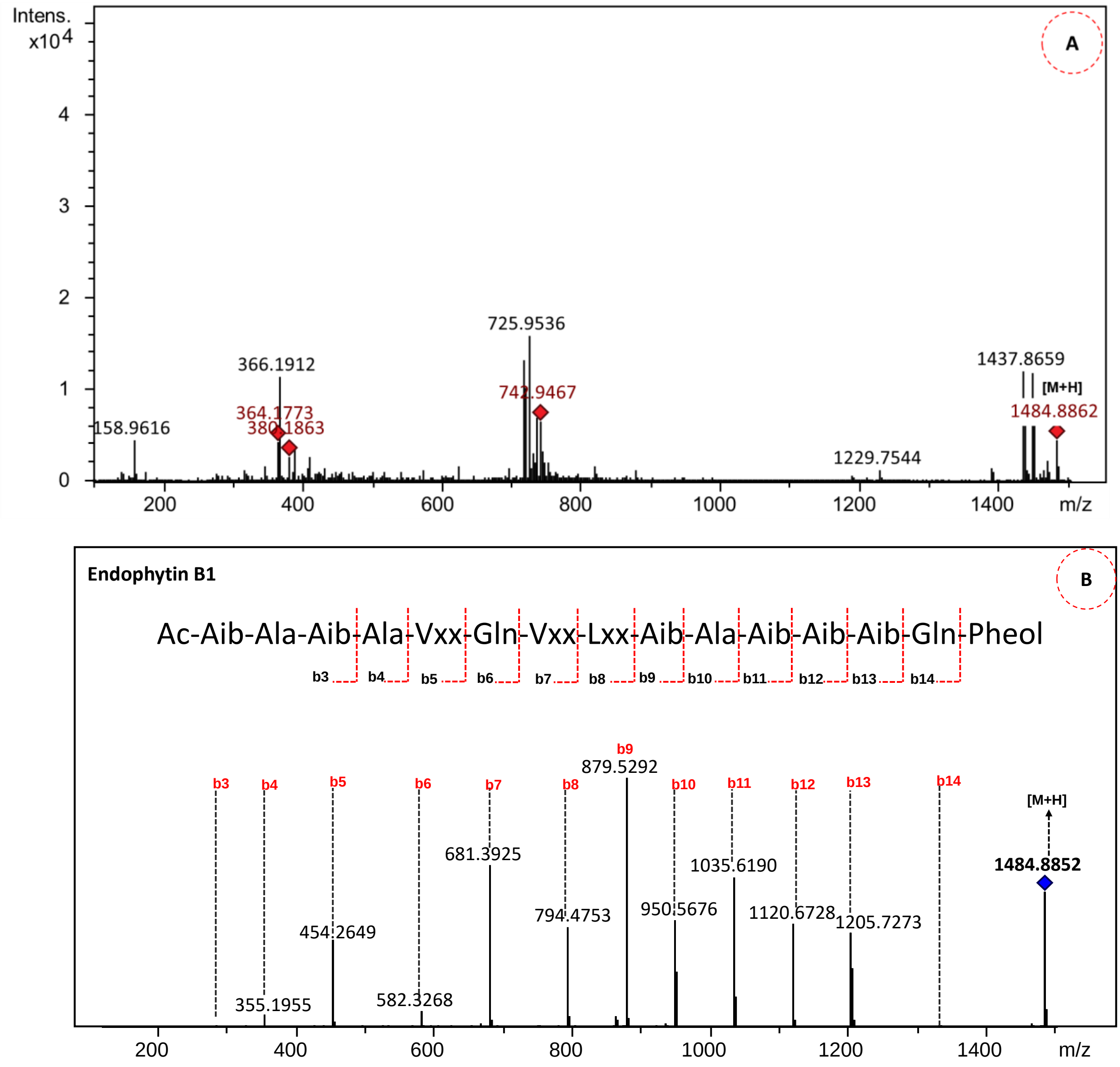

Figure S24: MS (**A**) and MS/MS (**B**) spectra of **endophytin B2.**

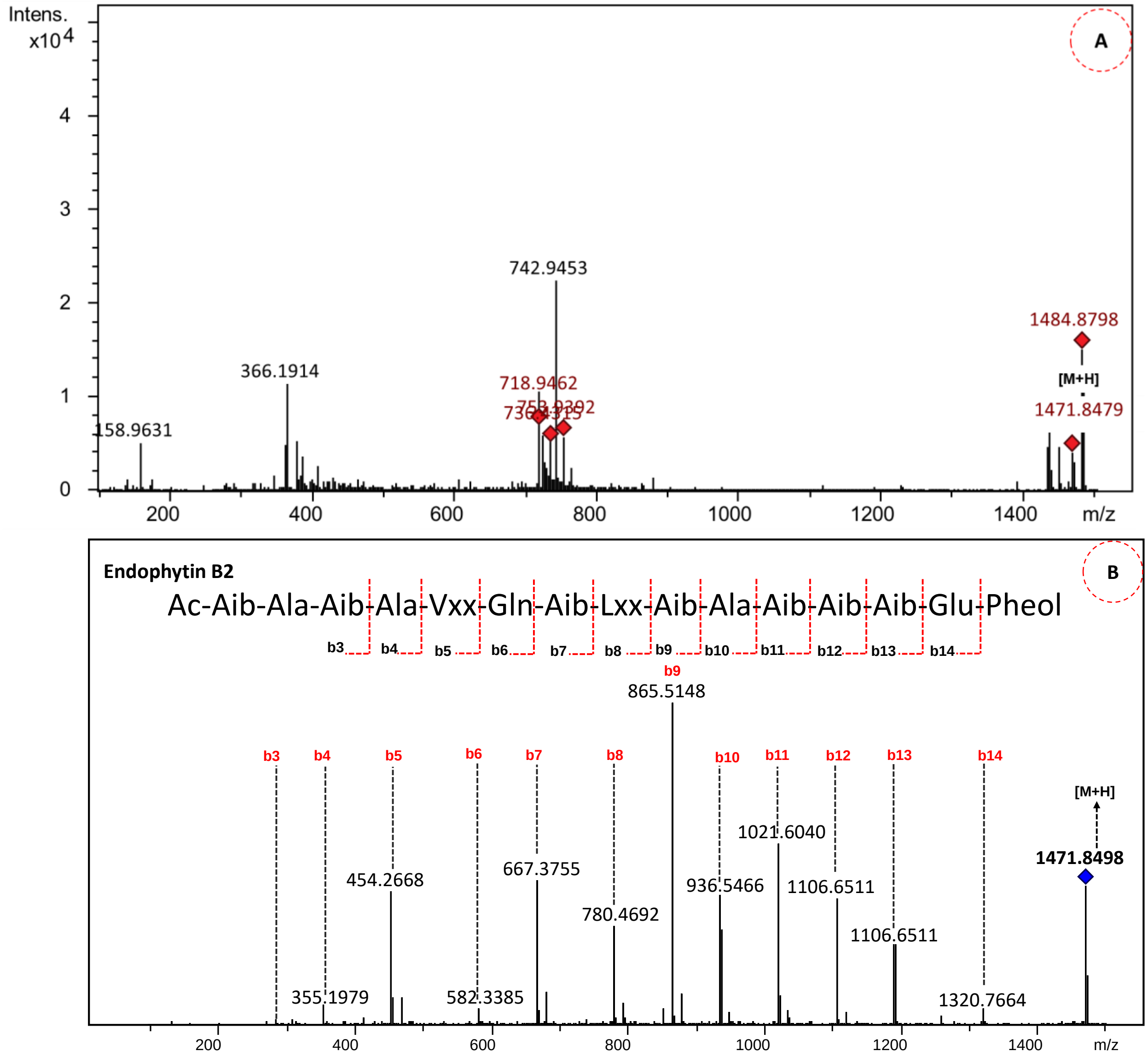

Figure S25: MS (**A**) and MS/MS (**B**) spectra of **endophytin B3.**

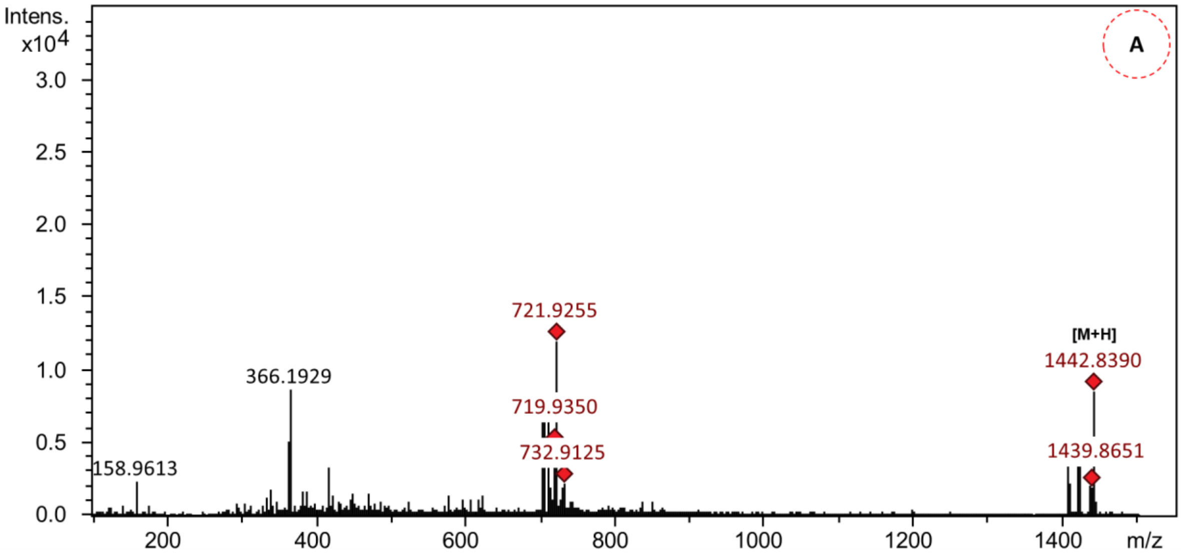

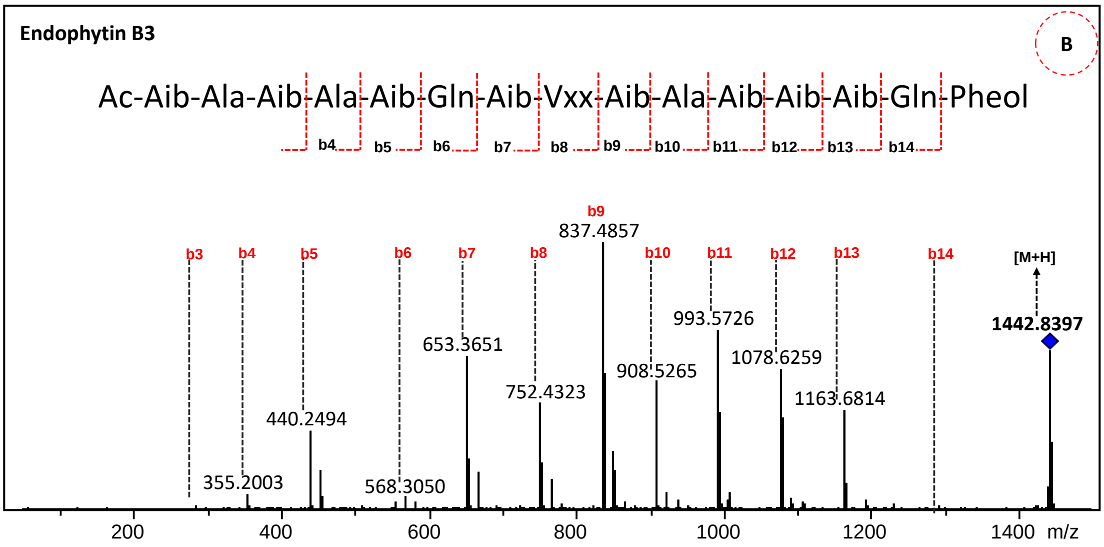

Figure S26: MS (**A**) and MS/MS (**B**) spectra of **endophytin B4.**

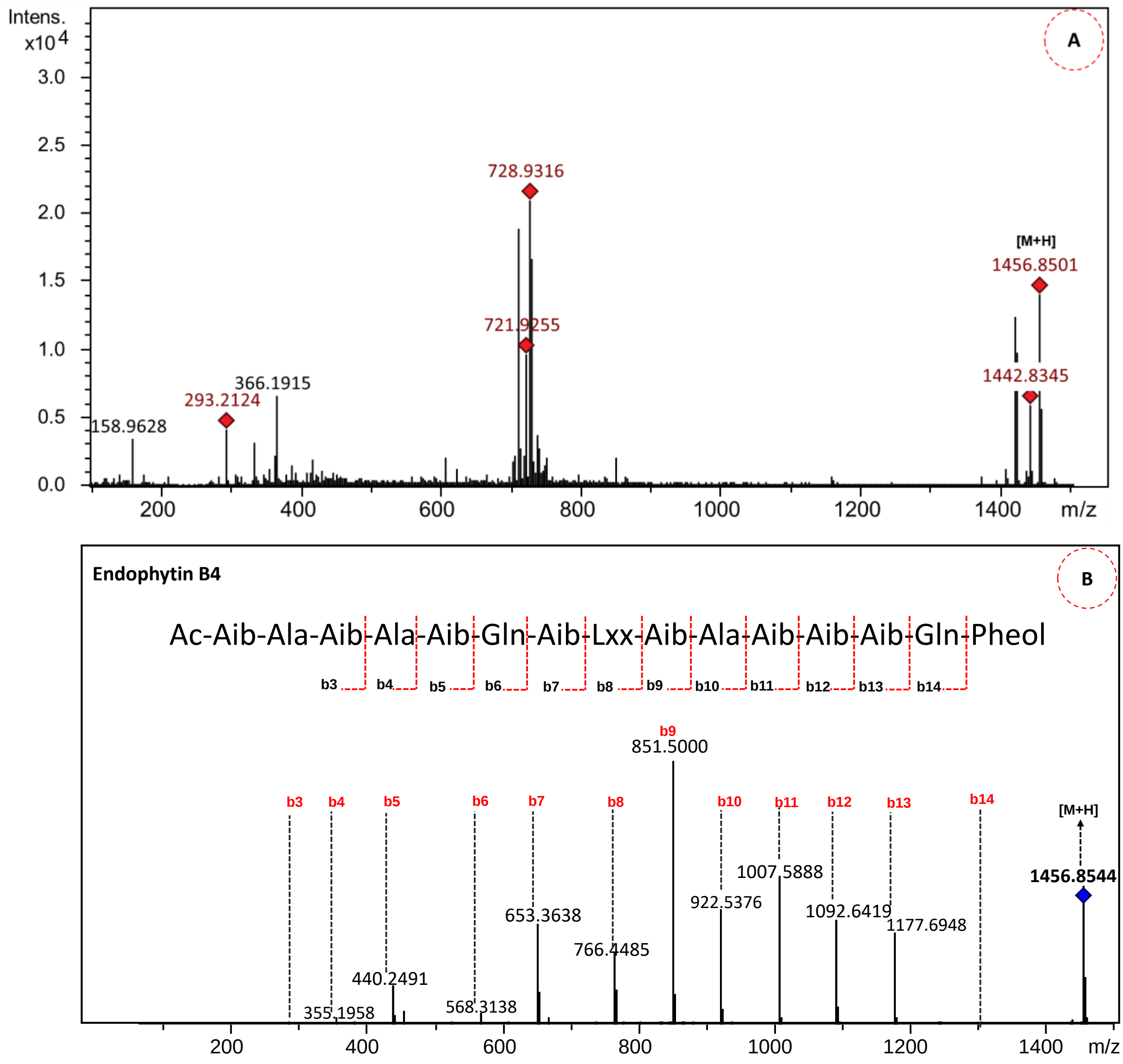

Figure S27: MS (**A**) and MS/MS (**B**) spectra of **endophytin B5.**

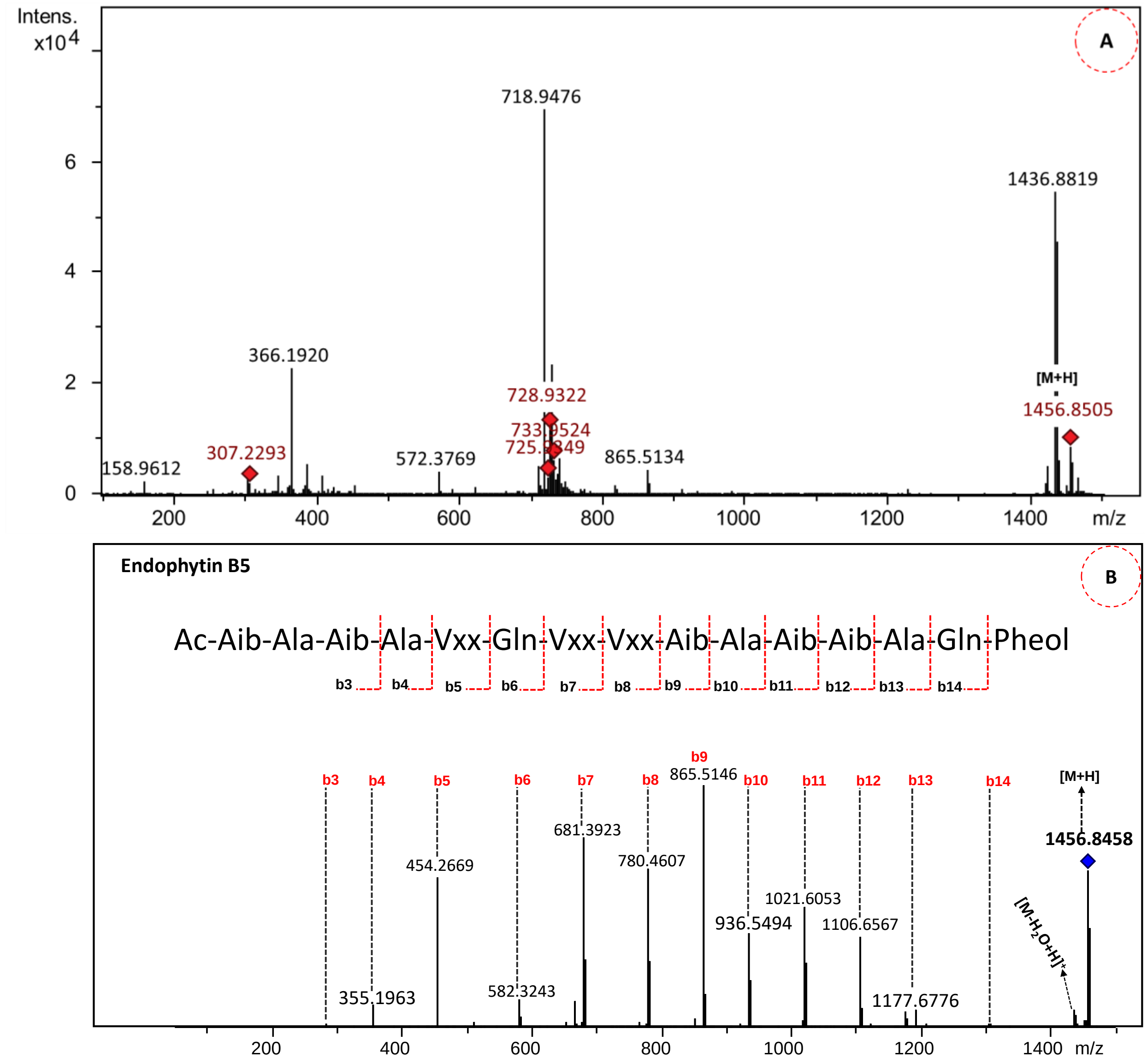

Figure S28: MS (**A**) and MS/MS (**B**) spectra of **endophytin B6.**

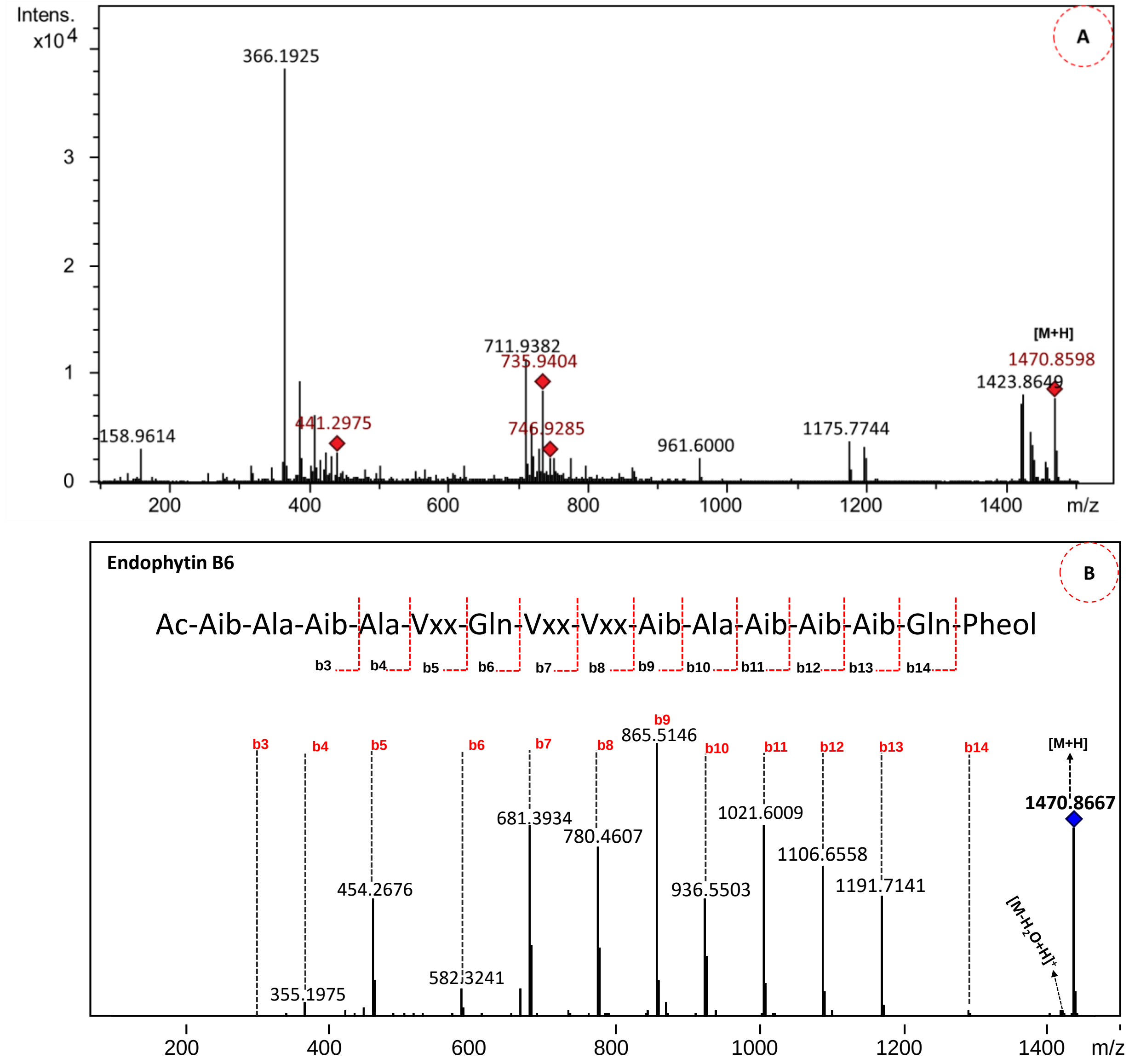

Figure S29: MS (**A**) and MS/MS (**B**) spectra of **endophytin B7.**

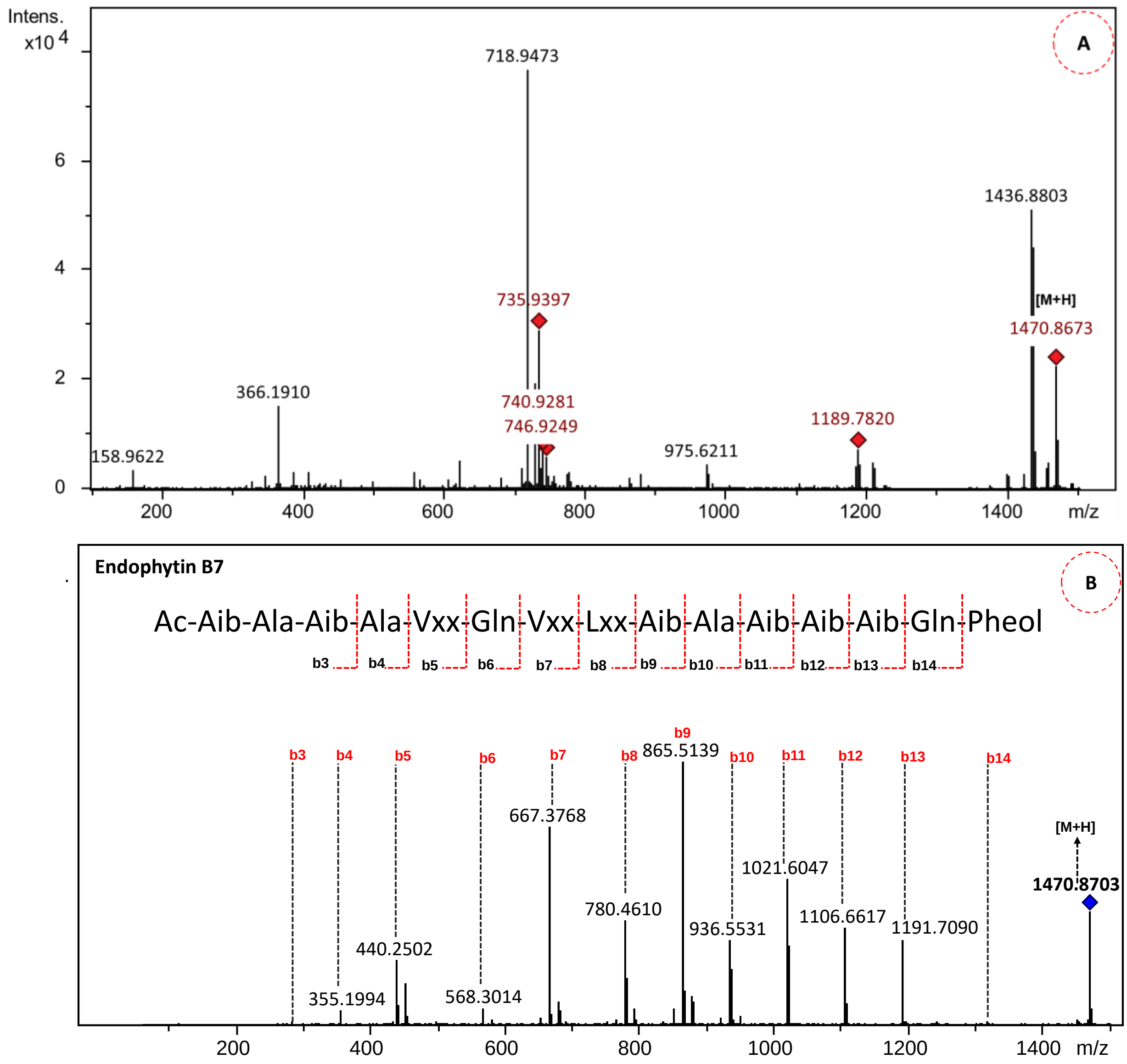

Figure S30: MS (**A**) and MS/MS (**B**) spectra of **endophytin B8.**

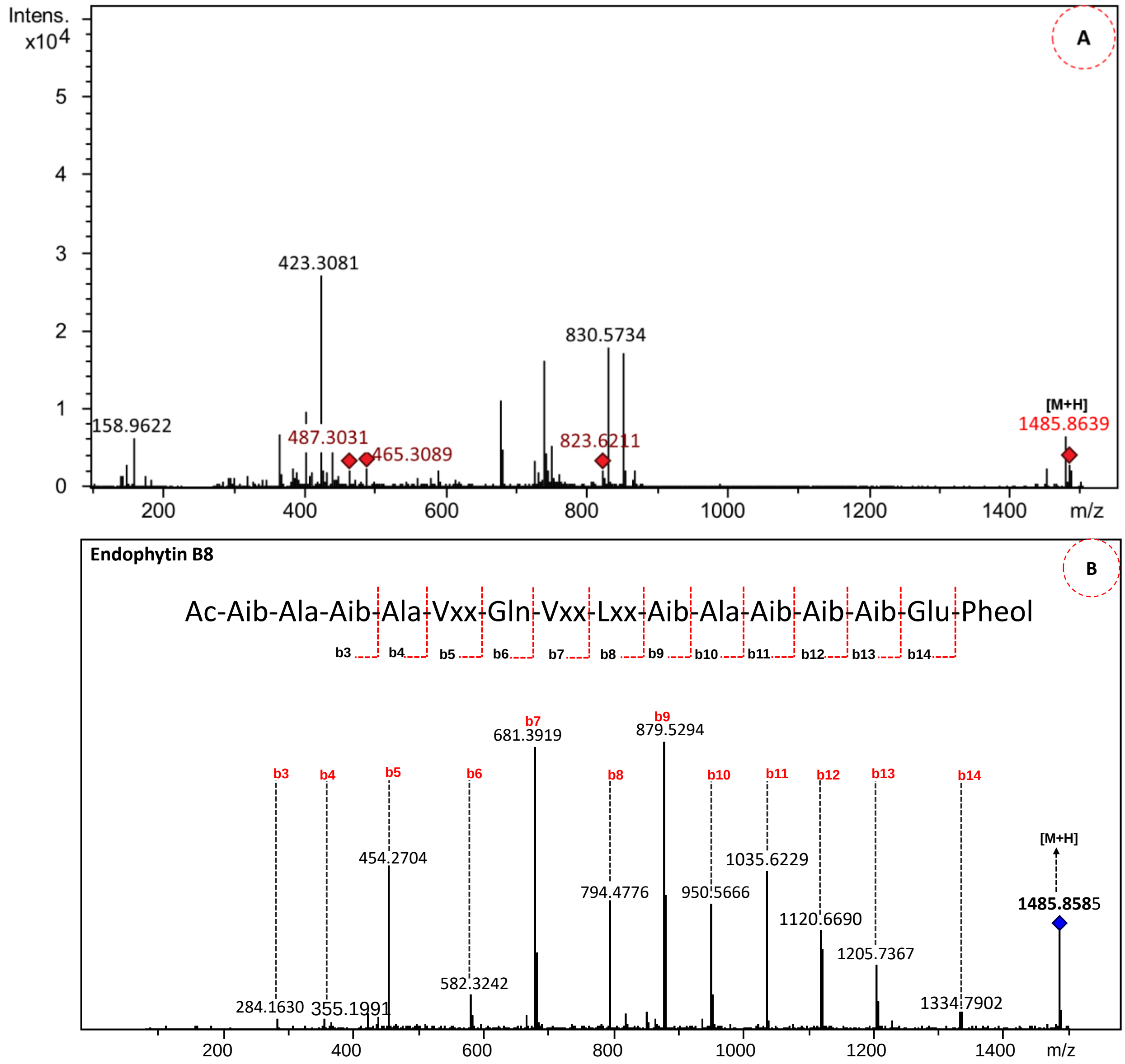

Supplement: Supplementary file 1 [file metabolites-13-00221-s001.zip › metabolites-2162124-supplementary.pdf]
